# Resource allocation under uncertainty and equity constraints in Parkinson’s disease care: An econometrically grounded machine learning framework

**DOI:** 10.1371/journal.pone.0360944

**Published:** 2026-11-03

**Authors:** Shekoofeh Sadat Momahhed, Atefehsadat Haghighathoseini

**Affiliations:** 1 PhD in Health Economics, National Centre for Health Insurance Research, Tehran, Iran; 2 Health Services Research (concentration: Knowledge Discovery & Health Informatics) Department of Health Administration, Policy, and Informatics (HAPI) George Mason University Fairfax, Fairfax, Virginia, United States of America; Covenant University, NIGERIA

## Abstract

**Objective:**

To apply an integrated causal-inference and welfare-optimization framework to Iranian Parkinson’s disease (PD) inpatient insurance claims, estimating how resource-allocation decisions relate to recovery, discharge risk, cost and financial burden; testing whether these relationships vary across patient subgroups; and assessing whether budget-neutral reallocation could improve efficiency and equity simultaneously.

**Setting:**

Six provinces of Iran, using de-identified administrative inpatient claims obtained from the Iran Health Insurance Organization for admissions with a primary or secondary diagnosis of PD during the 2023–2025 observation period.

**Design:**

Retrospective claims-based cohort study. Transaction-level records (n = 21,121) were aggregated into 1,813 hospitalization episodes and analyzed through a six-stage pipeline: gradient-boosted predictive models; double machine learning and augmented inverse-probability weighting for average treatment effects, restricted to an admission-time-only, pre-treatment severity adjustment; causal forests for subgroup heterogeneity; concentration-index decomposition for socioeconomic inequality; constrained integer-linear-programming optimization for reallocation scenarios; and bootstrap, E-value and design-effect sensitivity analyses.

**Results:**

Predictive models showed acceptable-to-strong discrimination (recovery, AUC-ROC 0.709; unsuccessful-discharge proxy, AUC-ROC 0.870) and explained 81.3% of variance in total cost. Prolonged length of stay, treated as an exploratory, within-episode exposure rather than a causal one because it is realized during the hospitalization itself, was associated with a 6.4-percentage-point lower discharge proxy and 200% higher cost (E-value limit 1.93–4.04). Higher insurance-coverage generosity, the study’s pre-treatment, policy-assignable exposure, was associated with an approximately 40% reduction in cost (E-value limit 2.19) and no significant change in the discharge proxy; effects were consistent across age, severity, hospital sector, rurality and socioeconomic rank, with an attenuated estimate in a small private-hospital subgroup. Recovery was concentrated among higher-socioeconomic episodes, while cost, out-of-pocket burden and length of stay were concentrated among lower-socioeconomic episodes; age and hospital ownership, not disease severity (which contributed only −0.8% to +6.2% of the observed gradients), were the leading correlates. A coverage-based, budget-neutral reallocation scenario reached 35.8% of the cohort within the observed budget. Ninety-eight percent of significant associations remained robust to plausible within-patient clustering, the principal limitation arising from the absence of a patient identifier in these claims data.

**Conclusion:**

Insurance-coverage generosity showed the strongest evidence of a causal, cost-reducing effect, while socioeconomic inequity in PD hospitalization outcomes was driven primarily by administrative rather than clinical-severity factors. The reallocation scenarios illustrate model-based, budget-neutral efficiency-equity gains and should be read as decision-support for phased piloting, not as empirically demonstrated policy effects. Patient-level claims linkage and validated clinical severity measures are needed to extend this framework toward genuine longitudinal outcomes.

## Introduction

Parkinson’s disease (PD) is the second most prevalent neurodegenerative disorder worldwide and one of the fastest-growing neurological conditions in terms of absolute burden [1,2]. Global Burden of Disease estimates through 2021 document a sustained rise in age-standardized prevalence and incidence, with disability-adjusted life years intensifying disproportionately in low- and middle-income countries as mortality accelerates and population ageing compounds the epidemiological transition [1–3]. This trajectory has been characterized as an emerging “Parkinson pandemic,” driven jointly by demographic ageing and possible environmental exposures [4], a framing echoed by recent commentary describing PD as among the fastest-growing neurological conditions confronting health systems worldwide [5]. Advanced PD generates markedly higher direct and indirect costs than early-stage disease across high-income settings [6]; Medicare data confirm that motor symptoms and treatment-related adverse events independently predict greater resource use and cost [7], late-stage parkinsonism imposes the heaviest burden across European systems through formal care, hospitalization, and informal caregiving [8], and Canadian estimates place the aggregate annual burden in the billions of dollars [9].

Iran exemplifies these pressures in an accelerating form. The Iranian Parkinson’s Disease Registry reports a standardized prevalence of 223 per 100,000 across six provinces, with 70% of patients classified as late-onset PD [10], and national trend data show that Iran’s age-standardized prevalence and incidence rose sharply between 1990 and 2021, with the highest burden concentrated in East Azarbayejan province and closely tied to social development indicators [11]. These pressures are already financially acute: a substantial share of Iranian households experience catastrophic health expenditure, with a meaningful proportion impoverished by health-related costs [12]. Iran’s health insurance system remains fragmented, with equity concerns and coverage gaps for diagnostic imaging, outpatient neurology, rehabilitation, and advanced interventions such as deep brain stimulation [13]. Family caregivers absorb much of the resulting burden absent adequate long-term care [14], and although the 2014 Health Transformation Plan expanded inpatient access, chronic neurodegenerative conditions remain inadequately prioritized within a fragmented, poorly coordinated system [15].

Despite this well-documented burden, a critical knowledge gap persists regarding how insurance-level resource allocation decisions causally shape PD outcomes. Health technology assessment and cost-effectiveness analyses have confirmed the value of specific PD interventions such as deep brain stimulation [16], and personalized, nurse-led care frameworks have been proposed to better match services to need [17]; yet both rely largely on data and assumptions from high-income settings with limited transferability to LMIC contexts. Emerging evidence indicates that PD clinical care varies substantially across centers, often reflecting institutional patterns rather than individualized need, with services such as physical and mental-health therapy frequently underused until after adverse outcomes occur [18] – underscoring the need for “precision public health” approaches to optimizing the distribution of specialist care, diagnostics, and essential drugs [19]. Causal machine learning offers a promising route toward such precision: DML has been used to isolate the causal effect of anti-dementia drug policy on economic burden in related neurodegenerative populations [20], and causal-inference methods are increasingly applied within PD itself to assess treatment effects on survival and to build causally informed feature-selection pipelines for severity monitoring [21,22]. Critically, real-world insurance claims data available in Iran have not been leveraged to answer causal or equity questions system-wide, leaving policymakers without the evidence needed to guide resource reallocation.

This study addresses that gap by applying an integrated causal-inference and welfare-optimization framework to Iranian PD insurance claims data, structured across five linked stages: gradient-boosted outcome prediction; Double Machine Learning to estimate average treatment effects of length-of-stay, coverage generosity, and service intensity [23]; Causal Forests, within the generalized random forest framework, to characterize treatment-effect heterogeneity across subgroups [24,25]; concentration-index decomposition to quantify socioeconomic inequity; and constrained integer-linear-programming optimization to identify budget-neutral reallocations, with uncertainty propagated throughout via bootstrap inference and Rosenbaum-bounds sensitivity analysis. We ask three questions: how do resource-allocation decisions causally affect recovery, readmission, cost, and financial burden; do these effects vary across patient subgroups; and can budget-neutral reallocation simultaneously improve efficiency and equity?

As the literature review below details, each of these methods – DML, Causal Forests, concentration-index decomposition, and constrained optimization – is individually well established, and each has previously been applied, separately, to neurodegenerative or chronic-disease populations elsewhere. The contribution of this study is therefore not novelty at the level of individual techniques, but their integration into a single, policy-oriented analytical pipeline, applied to Iranian administrative PD insurance data. Methodologically, the study offers a replicable causal-equity-optimization framework for LMIC health systems facing comparable pressures. Empirically, it generates causal and equity evidence for a national PD population in a setting where prior analyses have been almost exclusively descriptive or predictive. Practically, it translates these estimates into budget-neutral reallocation scenarios directly relevant to insurer and public-policy decision-making in Iran and comparable middle-income contexts.

## Literature review

A growing body of work has begun applying causal machine learning to chronic and neurodegenerative disease policy questions. DML addresses confounding by indication – the tendency for more severely affected patients to receive more intensive treatment – by using flexible machine-learning models to jointly estimate treatment assignment and outcome equations, isolating an orthogonalized causal effect [23]. DML was first applied in adjacent dementia research to quantify the causal effect of anti-dementia drug use on economic burden [20], and its use within PD has since expanded to causal survival analysis of dopaminergic therapy and to causal graph-based feature selection for remote severity monitoring, prioritizing features with a demonstrated causal link to progression over those merely associated with it [21,22]. Complementing average-effect estimation, Causal Forests within the generalized random forest framework recover conditional average treatment effects and characterize subgroup heterogeneity without requiring pre-specified interaction terms [24,25].

Beyond effect estimation, a parallel literature addresses how estimated effects should be translated into allocation decisions. Bi-level contextual-bandit frameworks optimize subgroup-level budget allocation under delayed feedback, explicitly modelling the lag between resource investment and observable outcomes in chronic disease management [26]. Fair reinforcement-learning frameworks embed counterfactual and group-level equity metrics directly into the reward function, so scarce resources such as diagnostic equipment or hospital beds are distributed without reproducing historical bias [27], while frameworks combining treatment-effect learning with online allocation reconcile treating patients now with continuing to learn which interventions work best [28]. In PD specifically, a cost-aware multimodal monitoring architecture assigns patients to budget-tiered assessment pathways – reserving expensive comprehensive evaluations for cases where diagnostic return justifies the expense – illustrating how constrained optimization can be embedded into clinical monitoring workflows [29]. Markov decision processes have similarly derived optimal monitoring policies under budget and adherence uncertainty [30], and two-stage conformal-prediction frameworks now provide calibrated uncertainty intervals around medication-need forecasts to guide which patients warrant closer monitoring [31].

Insurance structure is a recurrent determinant of PD care quality internationally. In South Africa, patients in the private sector report substantially higher quality of life and treatment access than public-sector users, who rely predominantly on basic medication under considerable resource constraints [32]; in Brazil’s public Sistema Único de Saúde, incomplete capture of medication use in administrative claims constrains system-level analysis despite frequent PD-related outpatient and inpatient utilization [33]. Insurance type and related administrative variables have also been shown to meaningfully predict discharge destination in nationwide cohort studies, supporting their use in multidisciplinary discharge planning and post-acute resource allocation [34]. Policy-level insurance interventions can shift outcomes at scale: the introduction of long-term care insurance in rural China has been shown, using difference-in-differences designs, to reduce medical-expenditure burden and improve subjective well-being among vulnerable older adults [35].

Equity constraints persist even where formal coverage is comparatively broad. Spatial-accessibility analyses using enhanced floating-catchment-area methods in Ontario, Canada find that geography, socioeconomic status, and demographic characteristics continue to shape utilization of PD services despite near-universal insurance [9]. In the United States, social determinants of health – including income and connectivity – are independently associated with PD prevalence and access, although observational designs limit causal interpretation of these associations [36]. Access to advanced, device-aided PD therapies remains constrained by health-system capacity and specialized surgical infrastructure, and social-return-on-investment analyses suggest that conventional cost-effectiveness frameworks systematically understate the value of expanding such access by omitting family and caregiver spillovers [37]. These international patterns closely mirror Iranian evidence on catastrophic and impoverishing health expenditure among households facing chronic illness [38], and on the concentration-index and horizontal-inequity decomposition methods – the Erreygers-corrected concentration index and Wagstaff-type decomposition – used to formally attribute socioeconomic gradients in health and payments to specific structural drivers [39–41].

Taken together, this literature establishes three points relevant to the present study. First, causal machine-learning methods – DML, Causal Forests, and their fairness- and budget-aware extensions – are individually well developed and have already been applied, separately, to neurodegenerative and chronic-disease policy questions outside Iran [20–31]. Second, formal equity-measurement techniques capable of decomposing socioeconomic gradients in health and payments are mature and have been applied extensively to catastrophic-expenditure and horizontal-inequity questions internationally and within Iran’s broader health system [38–41]. Third, and most consequentially, no identified study has combined causal effect estimation, heterogeneity analysis, formal equity decomposition, and constrained welfare optimization within a single analytical pipeline applied to real-world PD insurance claims, and none has done so for Iran, where administrative claims data – despite their availability – remain underutilized for causal or equity-focused policy analysis [10,11,32,33]. Existing PD-focused machine-learning applications in the Iranian and broader LMIC context remain largely descriptive or predictive rather than causal, and formal equity analyses of PD specifically, as distinct from chronic disease generally, remain scarce. It is this combination of methodological integration and contextual application to an underexamined, high-burden LMIC setting that the present study is designed to address.

## Methods

### Source of data

This study used de-identified administrative health insurance claims data obtained from the Iranian Health Insurance Organization (IHIO). The dataset comprised inpatient admissions for patients with a primary or secondary diagnosis of Parkinson’s disease across six provinces of Iran. The underlying claims covered admissions from March 6, 2023 through March 18, 2025, with the final included claim in the analytic dataset dated March 20, 2025 (the later of that episode’s admission or discharge date). Dates in the original administrative records were recorded according to the Jalali (Persian) calendar and were consistently converted to the Gregorian calendar for data processing, analysis, and reporting. The complete de-identified dataset used for the present analysis was received from IHIO on February 24, 2026 — after the close of the observation period, consistent with the claims-run-out time typically needed for administrative data to finalize. The observation period was defined according to the dates of the underlying inpatient claims and was therefore independent of the date on which the dataset was received.

The raw dataset comprised 21,121 claim transaction records, each representing a single billable service line submitted to the insurer within a hospitalization episode. Variables captured at the transaction level included patient demographics (age, sex), geographic identifiers (province), institutional characteristics (hospital ownership type), clinical information (admitting and discharging physician specialty, service group, discharge status), admission and discharge dates, and a comprehensive set of financial fields: total service cost, total amount requested from the insurer, total amount paid by the insurer, and the patient’s out-of-pocket share. Although individual patient identifiers were unavailable due to data anonymization protocols, each record contained sufficient administrative detail to reconstruct discrete hospitalization episodes, as described in the following section.

### Data preprocessing and sample refinement

The raw transaction records underwent a multi-stage preprocessing pipeline to produce a clean, analytically suitable episode-level dataset. In the first stage, all column names were standardized to resolve inconsistencies arising from trailing whitespace, capitalization differences, and minor label variations across data extracts. Date fields for admission and discharge were then parsed and validated, and length of stay (LOS) was computed directly from the difference between these dates to serve as the ground-truth measure; where this derivation yielded implausible or missing values, the recorded LOS field was used as a fallback. Temporal attributes including admission year, month, day of week, and a weekend admission flag were derived from the parsed dates to support subsequent stratification.

Missing values were addressed through targeted imputation: categorical fields such as insurance fund, gender, service group, and discharge status were imputed using the within-sample mode, financial fields with missing values were assigned zero on the basis that their absence reflects no payment activity rather than data loss, and age was imputed at the sample median. All numeric fields were then coerced to appropriate data types, and a gender harmonization procedure resolved heterogeneous encodings—spanning English, Persian, and abbreviated forms—into a binary Male/Female variable. Records missing critical operational fields (LOS, total cost, or admission/discharge dates) were removed from the dataset.

Outlier treatment was applied using an IQR-based fencing procedure with a conservative 3 × multiplier, chosen to preserve clinically legitimate extreme values common in neurological inpatient care while censoring implausible data artefacts. Variables treated included LOS, age, total cost, total requested, total paid, and insured share; values falling outside computed fences were winsorized to the fence boundaries rather than deleted. Same-day discharges (LOS = 0) were flagged for sensitivity analyses. A set of logical consistency checks was then applied: records where discharge preceded admission were removed, insured share values exceeding total cost were capped at total cost, total paid values exceeding total requested by more than a 1% tolerance were capped at the requested amount, and residual negative financial values were set to zero. Exact duplicate rows were subsequently identified and removed at the transaction level.

The final and most consequential transformation was the aggregation of transaction-level records into hospitalization episodes. Because a single inpatient admission typically generates multiple claim lines—reflecting distinct service categories billed separately to the insurer—analyzing transactions as independent observations would violate the assumption of unit independence and artificially inflate the effective sample size. An episode was defined by the unique combination of province, sex, age, insurance fund, admission date, and discharge date. Within each episode, financial fields were summed to represent the total episode-level expenditure, LOS was taken as the maximum across constituent transactions, and administrative and clinical attributes were carried forward from the first transaction of each episode. Four analytically central derived variables were constructed at the episode level: coverage ratio (total paid divided by total requested, bounded between 0 and 1), patient out-of-pocket burden (the insured share), patient out-of-pocket ratio (insured share as a proportion of total cost), and cost per inpatient day (total cost divided by LOS). This aggregation reduced the dataset from 21,121 transaction records to 1,813 unique hospitalization episodes, which constitute the unit of analysis for all subsequent modeling.

### Ethics approval and consent to participate

This study used secondary, non-identified administrative healthcare claims data obtained from the Iran Health Insurance Organization (IHIO). No direct contact, intervention, or interaction with human participants was involved. All data were fully anonymized prior to access and analysis, and the research team had no means of identifying individual patients.

Although the study did not involve human subjects in the conventional experimental sense, ethical approval was obtained due to the sensitive nature of healthcare administrative data. The study protocol was reviewed and approved by the School of Public Health and Allied Medicine at Tehran University of Medical Sciences under the ethical approval code IR.TUMS.NIHR.REC.1403.015.

All analyses were conducted in accordance with relevant institutional guidelines and regulations, and the principles of the Declaration of Helsinki were strictly followed throughout the study.

Given the retrospective nature of the study and the use of anonymized administrative data, the requirement for informed consent was waived by the Tehran University of Medical Sciences National Institute for Health Research Ethics Committee.

### Variables

#### Initial (raw) features.

The initial data for this study is based on raw inpatient insurance claims linked to admissions for Parkinson’s disease (PD). An electronic record serves as a comprehensive compilation of the entire hospitalization experience, encompassing detailed information across demographic, clinical, financial, administrative, and provider sections. This extensive, episode-oriented model has provided a solid foundation for accurately tracking expenses and has guided a framework for fairly distributing resources in the context of inpatient PD care in Iran.

An overview of the raw features included in the inpatient Parkinson claims dataset is provided in Table 1.

**Table 1:** Summary of raw features from inpatient Parkinson claims dataset.

Feature Name	Description
Gender	Patient’s sex (male/female)
Admission Date	Date of hospital admission
Discharge Date	Date of hospital discharge
Age	Age of the patient at the time of admission
Total Cost	Total medical cost incurred for the episode
Insured Person’s Share	Out-of-pocket payment made by the insured individual
Total Claimed Shares	Total amount claimed by the healthcare provider
Total Paid Shares	Total amount reimbursed by the insurer
Length of Stay	Duration of inpatient care in days
Discharge Status	Patient’s clinical outcome upon discharge (e.g., recovered, deceased)
Insurance Fund	Type of insurance coverage/fund category
Treating Physician Specialty	Medical specialty of the attending physician
Ownership	Ownership type of the treating hospital (e.g., academic, private, public)
Service Group	Type of medical service (e.g., surgery, internal medicine)
Diagnosis Type	Primary diagnosis code or label (e.g., acute appendicitis)
State	Geographic location (province) of the treating facility
Medical Record Type	Internal classification of the medical record (e.g., global, inpatient)

#### Feature engineering.

Following episode-level aggregation, a structured feature engineering pipeline was applied to the clean dataset to construct the analytical variables required across all five modeling steps. This pipeline addressed four distinct needs: the derivation of a clinically grounded severity proxy to control for case-mix in causal models; the operationalization of three binary treatment variables representing the resource allocation decisions of primary policy interest; the construction of outcome variables suited to both predictive and causal estimation; and the generation of socioeconomic, demographic, and interaction covariates to support equity measurement and welfare optimization. To address multicollinearity, variables exhibiting perfect or near-perfect collinearity were excluded prior to modeling: patient_oop was dropped due to perfect collinearity with insured_share, while total_requested and total_paid were excluded on the basis of extreme variance inflation factors (VIF > 300), retaining total_cost, coverage_ratio, and patient_oop_ratio as non-redundant financial representations. The full set of engineered features, their derivation, and their analytical roles are described in Table 2.

**Table 2:** Engineered features for Parkinson’s disease inpatient claims data.

Feature Name	Derived From	Description	Policy & Outcome Relevance	Why Useful for Modeling PD Care
adverse_discharge	discharge_status	Binary flag: 1 if discharge status contains death, deterioration, or against-medical-advice keywords.	Identifies episodes with the worst clinical outcomes; anchors the severity-risk model.	PD patients are at elevated risk of in-hospital deterioration; capturing adverse discharge separates routine stays from high-acuity episodes [42,43].
recovery_flag (Y1)	discharge_status	Binary flag: 1 if discharge status contains recovery, improvement, or routine discharge keywords.	Primary binary outcome for causal and predictive modeling.	Directly measures whether an episode resulted in clinical improvement — the central therapeutic goal in PD inpatient care [44].
severity_score	case_type, physician_specialty, service_group, gender (admission-time covariates only)	Cross-fitted, out-of-fold predicted risk of adverse discharge from a logistic regression trained on baseline (admission-time) covariates only, using K-fold stratified cross-fitting; percentile-ranked to [0,1]. Age is excluded from this model.	Controls for baseline case-mix in causal models without conditioning on variables measured during or after the episode (length of stay, cost per day, discharge outcome).	An admission-time-only, cross-fitted risk score is the standard approach to case-mix and risk adjustment in the causal-inference and clinical-outcomes literature, and avoids attenuating estimated treatment effects through downstream adjustment [45,46].
severity_tier	severity_score	Categorical variable (Low / Medium / High) derived by tercile cut of severity_score.	Enables subgroup stratification in descriptive analyses and heterogeneous effect estimation.	Supports identification of which severity tier benefits most from intensive resource allocation [47].
T1_prolonged_los	los_days, service_group	Binary: 1 if an episode’s length of stay exceeds a chosen quantile (default: median) of the within-service-group distribution. The threshold is a named, configurable parameter and is varied across a range of quantiles (0.40–0.75) in a robustness sweep at the causal-estimation stage.	Treatment variable 1; represents the decision to authorize an extended inpatient stay. Because length of stay is jointly determined by clinical severity, complications, and physician discretion rather than assigned independently of the patient, effect estimates are reported as adjusted associations under a stated identifying assumption, together with sensitivity bounds for unmeasured confounding.	Service-group stratification and the threshold robustness sweep ensure the estimated association is not an artifact of a specific cutoff; sensitivity analysis quantifies how much unmeasured confounding would be required to overturn the result [48,49].
T2_high_coverage	coverage_ratio	Binary: 1 if an episode’s coverage ratio (paid/requested) exceeds the dataset median.	Treatment variable 2; captures insurer generosity in reimbursement relative to billed claims.	Coverage generosity directly affects both insurer expenditure and patient out-of-pocket burden; heterogeneous effects across socioeconomic groups are the central equity question [25,26].
T3_high_intensity	n_transactions	Binary: 1 if the number of billed transaction lines per episode is at or above the 75th percentile of the distribution (high episode complexity).	Treatment variable 3; proxies service intensity via the volume of distinct billed care processes per episode.	Because service-group labels in this dataset are uniformly neurological, billing-transaction volume provides the most valid available proxy for differential service intensity within PD admissions [40,50].
Y2_unsuccessful_discharge_proxy	adverse_discharge, recovery_flag, severity_score	Binary: 1 if the episode ended in an adverse discharge (death, deterioration, or against-medical-advice), or if the patient did not recover and their baseline severity_score exceeded the cohort median; adverse-discharge episodes are included as positive cases rather than excluded. This is a constructed administrative proxy for a failed or premature inpatient episode, not an observed rehospitalization.	Outcome variable 2; proxies an unsuccessful or premature discharge in the absence of longitudinal follow-up data linking patients across admissions.	In the absence of follow-up data, this composite proxy captures clinically plausible signals of a failed discharge episode by combining the most severe administrative outcome (adverse discharge) with non-recovery among higher-baseline-severity patients; results are reported as changes in the risk of unsuccessful discharge rather than as readmission throughout the manuscript [46,51].
Y3_log_total_cost	total_cost	Natural log transformation of total episode cost: log(1 + total_cost).	Outcome variable 3; continuous measure of insurer and system expenditure per episode.	Total cost is right-skewed in insurance claims data; log transformation stabilizes variance and satisfies GBM and DML distributional assumptions [52,53].
episode_id	province, sex, age, insurance_fund, admission_date, discharge_date	Composite key uniquely identifying a hospitalization episode from transaction-level records, formed by grouping transactions sharing province, sex, age, insurance fund, and overlapping admission/discharge dates.	Defines the unit of analysis (N = 1,813 episodes) for every downstream model.	Necessary because the raw data are transaction-level rather than patient-level; in the absence of a patient-level identifier, episode uniqueness cannot be independently verified, and a small number of episodes may combine transactions from more than one patient sharing these attributes.
rural_flag	insurance_fund	Binary: 1 if the insurance fund label contains rural, NGO, or equivalent keywords.	Core equity covariate; distinguishes rural/underserved populations for Concentration Index decomposition.	Rural PD patients in Iran face compounded disadvantages in specialist access and out-of-pocket burden; this flag is essential for horizontal inequity estimation.
ses_rank	insurance_fund	Ordinal SES proxy (1–5) derived by mapping insurance fund type to a socioeconomic hierarchy.	Ranking variable for Concentration Index construction; governs wealth-based inequity measurement.	Insurance fund type is the most reliable SES proxy in Iranian claims data, reflecting formal employment status, public-sector affiliation, and coverage tier.
private_hospital	ownership	Binary: 1 if the admitting hospital is privately owned.	Controls for supply-side heterogeneity in cost and quality; relevant for equity analysis across ownership types.	Private hospitals in Iran bill at higher tariffs and serve different patient populations; omitting this covariate would confound both cost and outcome models.
gender_enc	gender	Binary encoding: 1 = Male, 0 = Female, after harmonizing all gender label variants.	Demographic confounder and equity stratifier in causal and equity models.	Sex differences in PD prevalence, progression, and care-seeking behavior necessitate explicit control and heterogeneity analysis.
age_sq	age	Squared age term added alongside linear age.	Captures non-linear age effects on cost, length of stay, and recovery characteristic of elderly PD populations.	The relationship between age and clinical outcomes in PD is curvilinear; including age squared allows the model to capture accelerating risk in the oldest age groups.
admission_season	admission_month	Categorical variable (0 = Winter, 1 = Spring, 2 = Summer, 3 = Autumn) derived from admission month.	Controls for seasonal variation in hospitalization rates and case mix.	PD-related hospitalizations for falls, pneumonia, and urinary tract infections exhibit seasonal patterns that could confound temporal comparisons.
age_x_severity	age (mean-centered), severity_score	Product interaction: (age minus sample mean age) x severity_score. Age is mean-centered before multiplication and is excluded from the severity_score model itself, so age, severity_score, and their interaction each carry non-redundant information.	Captures whether older patients disproportionately accumulate high-severity episodes — relevant for targeting welfare optimization.	The combined burden of advanced age and high disease severity is a plausible driver of catastrophic expenditure in PD; the interaction quantifies this compounding effect while avoiding collinearity with the age and severity main effects [54].
rural_x_coverage	rural_flag, coverage_ratio	Product interaction: rural_flag x coverage_ratio.	Tests whether coverage generosity has differential effects on rural versus urban patients — a key equity heterogeneity hypothesis.	If rural patients receive systematically lower coverage ratios, the interaction term reveals whether higher coverage yields larger recovery gains in this disadvantaged group [24].
female_x_rural	gender_enc, rural_flag	Product interaction: (1 – gender_enc) x rural_flag; equals 1 for rural female patients.	Identifies the doubly-disadvantaged subgroup (rural women) for targeted welfare allocation analysis.	Rural women in Iran face compounded barriers to PD care access, caregiver support, and insurance coverage; this term enables precise subgroup identification [55].
expected_los_prolonged	los_days, T1_prolonged_los, service_group	Median length of stay among prolonged-stay episodes within each service group; used as the counterfactual length of stay under T1 = 1.	Provides the bed-day cost of the T1 treatment for budget-constraint calibration in welfare optimization.	Welfare optimization requires projecting aggregate bed-day consumption under a proposed reallocation; service-group-specific medians keep projections clinically plausible [56,57].
expected_cost_high_cov	total_cost, T2_high_coverage	Median total cost among high-coverage episodes; counterfactual cost under T2 = 1.	Provides the expenditure estimate for the T2 treatment for the budget-neutral constraint in welfare optimization.	Analogous to expected_los_prolonged for the coverage-generosity treatment; calibrates the financial cost of extending high coverage to currently low-coverage patients [58].
private_hospital_low_n_flag / ses_rank_low_n_flag / rural_flag_low_n_flag	private_hospital, ses_rank, rural_flag	Binary flags: 1 if the stratum an episode belongs to (private-hospital status, a given SES tier, or rural status) contains fewer than 150 episodes in the full sample.	Downstream reporting: subgroup estimates falling below this threshold are presented with explicit wide-interval caveats rather than the same degree of confidence as large-sample estimates.	Makes estimation precision transparent rather than implicit in confidence-interval width alone, preventing small strata from being over-interpreted alongside larger, more stable subgroups.

For the Descriptive Epidemiology of Parkinson’s Disease Episodes, the Variance Inflation Factor (VIF) Analysis assessing multicollinearity among the initial variables, and the Correlation Matrix Heatmap of key cost and initial variables (Pearson coefficients), please refer to Supplementary A.

Table 2. Engineered features: derivation, description, policy relevance, and modeling rationale. Binary flags derived from discharge status were constructed using keyword matching across English and Persian discharge labels. Treatment variables T1–T3 were defined to achieve 25–50% prevalence in the analytical sample, a prerequisite for stable causal effect estimation [59]. Collinearity screening applied a |r| > 0.70 threshold and robust VIF analysis; variables exceeding VIF = 10 or exhibiting perfect collinearity were excluded from all downstream models [60].

### Statistical and analytical methods

The analytical pipeline proceeded through six sequential, interdependent stages: *(i)* outcome prediction with gradient boosting machines (GBM), *(ii)* causal effect estimation via Double Machine Learning (DML), *(iii)* heterogeneous treatment effect (HTE) estimation via Causal Forest, *(iv)* socioeconomic inequity measurement using the Concentration Index (CI) and horizontal inequity (HI) decomposition, *(v)* constrained welfare optimisation, and *(vi)* multi-layer uncertainty quantification. Each stage was designed to address a distinct research objective while building directly on the outputs of the prior stage, ensuring internal coherence across the causal, equity, and normative dimensions of the study. The sequence follows the causal inference pipeline formalised by Chernozhukov et al. and extended to equity-sensitive resource allocation by van Doorslaer and Wagstaff [23,40]. All analyses were conducted in Python 3.11 using scikit-learn 1.4, XGBoost 2.0, econml 0.15, and PuLP 2.8. Statistical significance was set at α = 0.05 (two-sided) throughout. No correction for multiple comparisons was applied across outcomes because each outcome addresses a theoretically and clinically distinct research question (recovery, readmission, cost, OOP burden, length of stay), and within each causal model a single pre-specified primary estimand is tested; this follows the guidance of Rothman [61], who argues that applying blanket multiplicity corrections to conceptually distinct hypotheses inflates Type II error without controlling a coherent family-wise error rate. Sensitivity of all inferences is evaluated in Stage VI. Full model specifications, hyperparameters, and diagnostic procedures for every stage are reported in S1–S7 Appendix in S1 Appendix.

#### Stage I: Outcome prediction with gradient boosting machines.

Five patient-level outcomes were modelled: clinical recovery at discharge (Y_1_ length of stay (Y_5_). Gradient Boosting Machines implemented via XGBoost [43] were selected because they impose no parametric functional form, accommodate the outcome-specific distributional families (binomial, Tweedie, Poisson) natively without transformation, are inherently robust to the multicollinearity documented in the variance-inflation analysis, and generate the out-of-fold nuisance predictions required for the Stage II cross-fitting procedure. Hyperparameters were tuned by Bayesian search (Tree-structured Parzen Estimator, 50 trials per outcome) [62,63] within stratified five-fold cross-validation. Binary outcomes were evaluated by AUC-ROC, AUC-PR, Brier score, and the Hosmer–Lemeshow statistic, with post hoc probability calibration (isotonic for Y_2_, Platt scaling for Y_1_) [64,65] and SHAP-based feature attribution [66]. Full hyperparameter grids, calibration diagnostics, and objective-function justification are reported in S1 Appendix.

#### Stage II: Causal effect estimation via double machine learning.

Stage II estimated population-average treatment effects (ATEs) of three resource-allocation decisions — prolonged length of stay (T_1_), high coverage generosity (T_2_), and high service intensity (T_3_) — on the risk of unsuccessful discharge (Y_2_) and log cost (Y_3_), using Double Machine Learning in the Partially Linear Regression form [23,67]:


$$\begin{array}{c} Y_{\text{i}}=\theta \;T_{\text{i}}+g(X_{\text{i}})+\varepsilon_{\text{i}},\;T_{\text{i}}=m(X_{\text{i}})+\nu_{\text{i}} \end{array}$$ (1)

where θ is the causal parameter of interest and g(·), m(·) are non-parametric nuisance functions estimated by the Stage I models under five-fold cross-fitting. The ATE was recovered by the Frisch–Waugh–Lovell projection of cross-fitted residuals,


$$\begin{array}{c} \hat{\theta }={\left(\Sigma_{\text{i}}\;{\hat{\nu }}_{\text{i}}^{\text{2}}\right)}^{-\text{1}}\;\Sigma_{\text{i}}\;{\hat{\nu }}_{\text{i}}\;{\hat{\varepsilon }}_{\text{i}} \end{array}$$ (2)

with inference via the influence-function sandwich estimator [68] (full expression in S2 Appendix in S1 Appendix in S1 Appendix, Eq S1). For the binary treatment T_2_, propensity scores were estimated by L_2_-penalised logistic regression, trimmed to [0.05, 0.95] to enforce the overlap assumption [69], and effects were estimated via Augmented Inverse Probability Weighting for double robustness [67]. DML was preferred over propensity-score matching, difference-in-differences, or instrumental-variable designs because it accommodates high-dimensional confounding without requiring a correctly specified propensity model, and because no valid instrument or repeated pre-post structure was available in this cross-sectional administrative dataset. Causal identification rests on conditional ignorability, overlap, and the stable unit treatment value assumption; overfitting guards, placebo permutation tests, and the T_1_/T_3_ threshold-robustness sweep are detailed in S2 Appendix in S1 Appendix, together with the full identification-assumptions discussion.

#### Stage III: Heterogeneous treatment effects via causal forest.

Stage III estimated patient-level Conditional Average Treatment Effects (CATEs),


$$\begin{array}{c} \tau (x)=E\left[Y(\text{1})-Y(\text{0})\right|\;X=x] \end{array}$$ (3)

using Causal Forest within the Generalised Random Forest framework [24,25], selected for its ability to discover treatment-effect heterogeneity across arbitrary covariate combinations without pre-specified interaction terms, and for its honest-splitting property, which yields asymptotically valid confidence intervals. The forest comprised 1,000 trees with minimum leaf size 5 [24]. Heterogeneity was formally tested using the Best Linear Predictor regression [23] (S3 Appendix in S1 Appendix, Eq S2); where out-of-bag R^2^ fell below a pre-specified signal-strength threshold, patient-level CATEs were replaced by pre-specified subgroup ATEs from eleven clinically defined strata, following Künzel et al. [70]. Ninety-percent bootstrap confidence intervals (300 replications) were computed following GRF convention [25]. Full forest specification, pseudo-outcome construction, and the out-of-bag decision rule are reported in S3 Appendix in S1 Appendix.

#### Stage IV: Equity measurement.

Stage IV quantified socioeconomic inequality using the Concentration Index (CI),


$$\begin{array}{c} CI=(\text{2}/\mu )\cdot Cov(Y_{\text{i}},\;r_{\text{i}}) \end{array}$$ (4)

where μ is the mean outcome and rᵢ is the fractional socioeconomic rank, computed across three SES proxies (insurance fund type, a composite SES rank, and rural/urban residence), with the Erreygers-normalised index applied to bounded binary outcomes [39] (S4 Appendix in S1 Appendix, Eq S3). The CI was preferred over the Gini coefficient because it captures both the magnitude and the socioeconomic direction of inequality [40]. Horizontal inequity (HI) — the inequality unattributable to legitimate health need — was estimated by indirect standardisation [71]:


$$\begin{array}{c} HI=CI(Y_{\text{i}})-CI({\hat{\text{Y}}}_{\text{i}}need) \end{array}$$ (5)

where Ŷᵢneed is the need-standardised expected outcome predicted from age, sex, and severity score. The Wagstaff–van Doorslaer decomposition [40] further attributed the CI to determinant-level contributions via a Linear Probability Model, chosen to preserve the exact additivity of the decomposition identity (S4 Appendix in S1 Appendix, Eq S4). All estimates carried bias-corrected and accelerated (BCa) bootstrap confidence intervals (1,000 replications). Full decomposition diagnostics and reliability flags are reported in S4 Appendix in S1 Appendix.

#### Stage V: Constrained welfare optimisation.

Stage V identified budget-neutral treatment reallocations maximising a utilitarian welfare function,


$$\begin{array}{c} max\;W=\Sigma_{\text{i}}\;[\alpha \cdot \tau_{\text{i}}(x_{\text{i}})\cdot t_{\text{i}}-\lambda \cdot OOP\_{delta}_{\text{i}}(t_{\text{i}})] \end{array}$$ (6)

subject to bed-day, insurer-cost, and hard equity constraints (S5 Appendix in S1 Appendix, Eqs S5–S7) requiring that high-severity patients be treated at least as frequently as low-severity patients, consistent with the ethical framework of Williams and with the Iranian health system’s stated equity objectives [72]. Binary Integer Linear Programming, solved by CBC branch-and-bound [73], was selected over continuous relaxation or greedy heuristics because treatment decisions are inherently binary and because ILP guarantees a globally optimal solution given the global coupling the equity constraint introduces across severity strata; a greedy heuristic served as a warm-start and robustness comparison. Three policy scenarios were evaluated — observed practice, welfare-optimal allocation, and equity-maximising allocation. Welfare weights (α, λ) were set to 1 in the base case, with a 4 × 4 sensitivity grid spanning plausible societal preferences reported in S5 Appendix in S1 Appendix.

#### Stage VI: Uncertainty quantification.

Stage VI propagated four distinct sources of uncertainty. Causal effect estimates carried analytical 95% confidence intervals from the influence-function sandwich variance; equity indices carried BCa bootstrap confidence intervals (1,000 replications) [74]; sensitivity to unobserved confounding was assessed via Rosenbaum bounds [75], reporting the critical Γ at which a finding would be nullified by hidden bias; and welfare conclusions were propagated through 500 CATE-perturbation replications to yield 95% credible intervals for total welfare, treatment rates, and the severity-targeting ratio. Full layer-by-layer specifications, including the direction-aware Rosenbaum p-value procedure and Monte Carlo precision checks, are reported in S6 Appendix in S1 Appendix.

### Identification assumptions and limitations

Causal identification in Stages II–III rests on conditional ignorability, overlap, and the stable unit treatment value assumption; these conditions, their empirical support in the administrative covariate set, and their formal robustness assessment via Rosenbaum bounds are discussed in full in S7 Appendix in S1 Appendix, together with three additional limitations concerning the normative (non-causal) status of the Stage V optimisation, the episode-level unit of analysis, and the six-province scope of the dataset.

## Results

### Stage 1: Predictive outcome models

Out-of-fold (OOF) discrimination and calibration were evaluated for the two binary Stage 1 outcomes, recovery (Y1) and the premature/unsuccessful discharge proxy (Y2), each trained on the full analytic sample (n = 1,813). The recovery model achieved an OOF area under the receiver-operating-characteristic curve (AUC-ROC) of 0.709 and an OOF average precision (AUC-PR) of 0.869 against a 76.1% positive-class prevalence, with a non-significant Hosmer–Lemeshow (HL) statistic after Platt-scaling calibration (χ^2^ = 6.39, p = 0.604). The discharge-risk proxy model, predicting a substantially rarer outcome (11.9% prevalence), achieved higher discrimination (AUC-ROC = 0.870) but a lower average precision (AUC-PR = 0.568), consistent with its lower base rate; isotonic, cross-validated calibration reduced its Brier score from 0.155 to 0.074, although the HL test remained formally significant (χ^2^ = 35.14, p < 0.001). The results of the Stage 1 models are presented in Table 3.

**Table 3:** Discrimination and calibration performance of the Stage 1 binary outcome models (recovery, Y1; premature/unsuccessful discharge proxy, Y2).

Outcome	n	Positive rate (%)	Calibration method	OOF AUC-ROC	OOF AUC-PR	Brier (raw)	Brier (calibrated)	HL χ^2^	HL p	HL pass
Y1 — Recovery	1,813	76.06	Platt scaling	0.709	0.869	0.162	0.162	6.39	0.604	Yes
Y2 — Premature/unsuccessful discharge proxy	1,813	11.91	Isotonic (CV)	0.870	0.568	0.155	0.074	35.14	<0.001	No*

*See interpretation below regarding the large-sample sensitivity of the Hosmer–Lemeshow test under class imbalance. AUC-ROC, area under the receiver-operating-characteristic curve; AUC-PR, area under the precision–recall curve (average precision); HL, Hosmer–Lemeshow; OOF, out-of-fold; CV, cross-validated.

Both binary models showed acceptable-to-strong discrimination on held-out folds, with AUC-ROC values comfortably above the 0.70 threshold conventionally regarded as informative for clinical risk models. The lower average precision obtained for Y2 reflects its markedly lower prevalence (11.9%) rather than weak model performance: average precision is mechanically bounded by the positive-class base rate, and a value of 0.568 against an 11.9% baseline represents a roughly five-fold enrichment over a non-informative classifier. The residual statistical significance of the Hosmer–Lemeshow test for Y2 after isotonic calibration is a well-recognised large-sample artefact: with n  =  1,813 and pronounced class imbalance, the HL statistic is sensitive to small deviations across probability deciles that carry negligible practical consequence, as illustrated by the substantial reduction in Brier score (0.155 to 0.074) achieved by the same calibration step. Because Y2 is constructed from same-day discharge, non-recovery and adverse-discharge indicators rather than from a linked subsequent hospital admission, it is reported throughout as an administrative discharge-risk proxy rather than as readmission, and the results below should be read as predictive validation of that discharge-risk construct rather than as evidence regarding rehospitalisation. It should also be emphasised that Stage 1 is a purely predictive exercise: the discrimination reported here establishes that the outcome constructs and engineered features carry genuine predictive signal, but does not by itself support a causal interpretation, which is addressed separately — under an explicit pre-treatment identification strategy — in Stage 2.

Predictive performance for the three continuous/count Stage 1 outcomes (Table 4) was strongest for total episode cost: the log-cost model (Y3) explained 81.3% of out-of-fold variance (R^2^ = 0.813; RMSE = 0.492; MAE = 0.373, natural-log scale), while the patient out-of-pocket-ratio model (Y4) and the length-of-stay model (Y5) explained smaller but still substantial shares of variance (R^2^ = 0.301 and 0.349, respectively; RMSE = 0.067 and 4.90; MAE = 0.046 and 2.89).

**Table 4:** Out-of-fold predictive performance of the Stage 1 continuous and count outcome models (log-total cost, Y3; patient out-of-pocket ratio, Y4; length of stay, Y5).

Outcome	Task / distribution	n	OOF RMSE	OOF MAE	OOF R²
Y3 — log-total cost	Gradient-boosted regression (squared error)	1,813	0.492	0.373	0.813
Y4 — Patient out-of-pocket ratio	Gradient-boosted Tweedie (variance power = 1.5)	1,813	0.067	0.046	0.301
Y5 — Length of stay (days)	Gradient-boosted Poisson (count)	1,813	4.899	2.894	0.349

RMSE, root-mean-squared error; MAE, mean absolute error; R^2^, coefficient of determination; OOF, out-of-fold. Y3 error metrics are computed on the natural-log scale, log(1 + total_cost), and are therefore currency-invariant. Where total-cost quantities are reported elsewhere in the manuscript in absolute monetary terms, values are expressed in purchasing-power-parity-adjusted international dollars (Int$), applying a conversion of approximately 146,000–150,000 Iranian Rials (IRR) per Int$ for the 2024 portion of the observation window and approximately 150,000–155,000 IRR per Int$ for the 2025 portion, consistent with the study’s stated 2023–2025 observation period.

The strong performance of the cost model (R^2^ = 0.813) indicates that the engineered administrative and treatment-related features — principally case type, prolonged length of stay, same-day discharge status, service intensity and service group — capture the great majority of systematic variation in inpatient expenditure, consistent with the well-documented role of length of stay and service intensity as dominant cost drivers in inpatient claims data. The comparatively lower R^2^ for the out-of-pocket ratio (0.301) and length of stay (0.349) reflects greater residual, patient- and provider-level heterogeneity that is not fully captured by administrative claims variables; this is an expected property of the underlying constructs rather than a modelling deficiency, since both quantities are also directly targeted, downstream, by the causal and equity analyses reported in Stages 2–4, which do not require a high Stage 1 predictive R^2^ in order to be valid.

Across the five Stage 1 models, prolonged length of stay (T1), service intensity (T3) and administrative case-mix descriptors (case type, ownership, physician specialty, state) recurred as leading predictors, while the admission-time-only severity score entered the top-ten predictor set for the discharge-risk-proxy (Y2) and out-of-pocket-ratio (Y4) models but was not among the top five for any outcome (Table 5; extended lists in S5 Table in S1 Appendix).

**Table 5:** Top five out-of-fold predictors retained by each Stage 1 outcome model, in rank order.

Outcome	1st	2nd	3rd	4th	5th
Y1 — Recovery	age	age_sq	T3_high_intensity	cost_per_day	private_hospital
Y2 — Discharge-risk proxy	case_type_enc	n_transactions	T1_prolonged_los	ownership_enc	state_enc
Y3 — log-total cost	case_type_enc	T1_prolonged_los	same_day_flag	T3_high_intensity	service_group_enc
Y4 — Patient OOP ratio	case_type_enc	same_day_flag	ownership_enc	T1_prolonged_los	insurance_fund_enc
Y5 — Length of stay	case_type_enc	T3_high_intensity	n_transactions	coverage_ratio	physician_specialty_enc

_enc denotes a label-encoded categorical administrative descriptor; T1_prolonged_los, T2_high_coverage and T3_high_intensity are the three treatment variables of primary policy interest (Table 2). OOP, out-of-pocket.

The recurrence of the treatment variables (T1_prolonged_los, T3_high_intensity) among the leading predictors of most outcomes is expected and appropriate at this predictive stage, because Stage 1 is designed to characterise the full observed association between administrative claims features and downstream clinical and financial outcomes, not to estimate a causal effect. This predictive use of the treatment variables is analytically distinct from the pre-treatment confounder set used for causal adjustment in Stage 2: the severity score used throughout the causal analyses is constructed exclusively from admission-time covariates (case type, physician specialty, service group and gender) via cross-fitted, out-of-fold prediction, and is not built from length of stay, cost, or discharge status. Its appearance only as a secondary (sixth-to-tenth ranked) predictor of the discharge-risk and out-of-pocket outcomes — rather than as a top-five driver that would suggest circularity with those same outcomes — is consistent with its intended admission-time construction. Age and its quadratic term are uniquely prominent predictors of recovery (Y1), consistent with the established relationship between advanced age and inpatient recovery trajectories in Parkinson’s disease.

### Stage 2: Causal effect estimation via double machine learning (DML)

Building on the descriptive and covariate-balance results presented above, Stage 2 estimated average treatment effects (ATEs) for three treatment definitions — prolonged length-of-stay (T1), high insurance coverage (T2), and high transaction intensity (T3) — using a cross-fitted, orthogonalized double machine learning (DML) estimator. For each specification, two nuisance functions were fitted by machine learning within cross-fitting folds: a treatment (propensity) model, E[T|X], and an outcome model, E[Y|X], and the ATE was recovered from the residual-on-residual regression of the two. Two outcomes were analysed: Y2, an unsuccessful-discharge proxy constructed from same-day discharge, recovery status and adverse-discharge flags (this proxy does not capture subsequent hospital re-admission and is not interpreted as such anywhere below); and Y3, total episode cost modelled on the natural-log scale. Because the log-cost outcome is modelled on a proportional scale, ATEs for Y3 are directly interpretable as approximate percentage effects (100 × (e^ATE − 1)) and are therefore invariant to currency; where absolute cost levels are reported elsewhere in the manuscript, nominal Iranian Rial (IRR) figures have been converted to purchasing-power-parity–adjusted international dollars (Int)usingannualPPPconversionfactorsofapproximately146,000–150,000IRR/Int) using annual PPP conversion factors of approximately 146,000–150,000 IRR/Int)usingannualPPPconversionfactorsofapproximately146,000–150,000IRR/Int for 2024 and 150,000–155,000 IRR/Int$ for 2025 (provisional, pending final International Comparison Program updates), via Cost(Int$) = Cost(IRR) / PPP factor.

Abbreviations used consistently throughout this section: ATE, average treatment effect; CI, 95% confidence interval (used only in this sense throughout the manuscript); SE, standard error; DML, double machine learning; E[T|X] and E[Y|X], treatment and outcome nuisance models; AUC-ROC and AUC-PR, area under the receiver-operating-characteristic and precision-recall curves; RMSE, MAE and R^2^, root-mean-square error, mean absolute error and coefficient of determination; PPP, purchasing power parity; Int$, PPP-adjusted international dollar.

Table 6 demonstrates the propensity (treatment) models for T1 and T2 each adjusted for the same 18 admission-time and administrative covariates. The T3 propensity model adjusted for 17 of these covariates, omitting the count of billed transactions (n_transactions) because this variable is used directly to construct the T3 exposure definition; retaining it in E[T|X] would reintroduce circularity between the exposure and its own predictor.

**Table 6:** Covariate adjustment sets used in the treatment (propensity) models for each DML specification.

Treatment specification	No. of covariates	Covariates
T1 — Prolonged length-of-stay	18	age, age^2^, admission year, admission month, admission season, weekend-admission flag, number of billed transactions, severity score, rural-facility flag, private-hospital flag, sex, state, insurance fund, case type, ownership type, physician specialty, age × severity interaction, female × rural interaction
T2 — High insurance coverage	18	identical set to T1
T3 — High transaction intensity (primary binary and continuous specifications)	17	identical set to T1/T2, excluding number of billed transactions

*Severity score is a cross-fitted, out-of-fold predicted risk of adverse discharge constructed exclusively from admission-time covariates (case type, physician specialty, service group and gender); it does not include length of stay, cost, or discharge status. Its construction and its role in the causal adjustment set are discussed under Table 2 *and immediately below.*

The covariate set is comprehensive with respect to observed demographic, administrative and payer characteristics, and the exclusion of the transaction count from the T3 propensity model directly protects the identification of that specification against circularity. The severity score itself has been reconstructed to remove a specific identification concern flagged in an earlier round of review: rather than being a composite of length-of-stay percentile, cost-per-day percentile and adverse-discharge status — all of which are determined during or after the hospital stay — it is now a cross-fitted, out-of-fold predicted risk of adverse discharge derived exclusively from admission-time covariates (case type, physician specialty, service group and gender), with age excluded from its own construction to avoid collinearity with the age terms already in the adjustment set (Table 2). Because it no longer contains length of stay, cost, or discharge status, the severity score used in Table 6 is a genuinely pre-treatment covariate for all three exposures and does not by itself introduce a post-treatment adjustment risk. This reconstruction still leaves an important limitation, which we state explicitly rather than resolve: the admission-time severity score is a proxy for clinical severity based only on administrative case-mix variables, and richer clinical information that would ideally underlie a severity adjustment — validated PD disease stage, UPDRS/Hoehn–Yahr measures, comorbidity burden, and prior utilisation — was not available in the linked claims data. For T1, whose exposure is itself prolonged length-of-stay, this means that although the adjustment set is no longer mechanically contaminated by post-treatment variables, residual unmeasured clinical severity may still confound the estimated association between length of stay and the outcomes; for this reason, the T1 estimates reported in Table 7 are labelled and interpreted as associational rather than causal throughout this manuscript, and the accompanying sensitivity analysis (E-values, Table 7) is presented as a bound on, rather than a resolution of, this residual concern. T2 and T3 are administratively or contractually determined exposures (insurance coverage tier and service-intensity category) that are not mechanically related to length of stay, cost, or discharge status at all, so the reconstructed severity score removes, rather than merely attenuates, the earlier post-treatment concern for those two specifications. A genuinely clinical confounder set (e.g., diagnosis/comorbidity burden, prior utilisation, and disease-stage information captured at or before admission) was not available in the linked claims data and is flagged as a priority for future data linkage; in its absence, causal language for T2 and T3 in this manuscript should be read as effects estimated under an unconfoundedness assumption conditional on the observed covariates, not as definitively identified causal effects.

**Table 7:** Double machine learning average treatment effect (ATE) estimates for the primary treatment specifications.

Treatment	Outcome	ATE (SE)	95% CI	z	p-value	E-value†	Approx. effect
T1 — Prolonged LOS	Unsuccessful-discharge proxy (Y2)	−0.065 (0.012)	−0.089, −0.041	−5.26	<0.001	2.53	−6.5 pp (associational)
T1 — Prolonged LOS	Total cost, log scale (Y3)	0.814 (0.025)	0.766, 0.862	33.19	<0.001	3.25	≈+125.6% (associational)
T2 — High coverage	Unsuccessful-discharge proxy (Y2)	0.000 (0.014)	−0.028, 0.028	0.02	0.984	—	null
T2 — High coverage	Total cost, log scale (Y3)	−0.320 (0.030)	−0.379, −0.261	−10.66	<0.001	—	≈−27.4%
T3 — High transaction intensity	Unsuccessful-discharge proxy (Y2)	−0.013 (0.013)	−0.038, 0.013	−0.98	0.326	—	null
T3 — High transaction intensity‡	Total cost, log scale (Y3)	0.052 (0.031)	−0.009, 0.114	1.67	0.096	—	≈+5.4%, not significant

†E-values quantify, for the associational T1 estimates only, the minimum risk-ratio association an unmeasured confounder would need with both treatment and outcome, over and above the measured covariates, to fully explain away the observed association; E-values were not computed for T2/T3 because those exposures are administrative levers rather than clinically endogenous states, so unmeasured-confounder sensitivity is addressed instead through the covariate and identification discussion above and the robustness checks in the Supporting Information. ‡The outcome nuisance model E[Y|X] for the T3–Y3 pairing retains the transaction count as a covariate (excluded only from the treatment model, E[T|X], to protect first-stage identification; see S4 Table in S1 Appendix); if part of the true treatment effect operates through transaction volume itself, this ATE should be read as a conservative lower bound.

Table 7 reports the cross-fitted DML ATEs for the three primary specifications. Prolonged length-of-stay was associated with a 6.5-percentage-point reduction in the unsuccessful-discharge proxy (ATE = −0.065, 95% CI −0.089 to −0.041, p < 0.001) and an approximate 125.6% increase in total episode cost (ATE = 0.814 log-points, 95% CI 0.766 to 0.862, p < 0.001). High insurance coverage showed no detectable effect on the discharge proxy (ATE = 0.0003, 95% CI −0.028 to 0.028, p = 0.984) but was associated with an approximate 27.4% reduction in cost (ATE = −0.320, 95% CI −0.379 to −0.261, p < 0.001). High transaction intensity, the primary (binary) T3 specification, showed no statistically significant effect on either the discharge proxy (ATE = −0.013, 95% CI −0.038 to 0.013, p = 0.326) or cost (ATE = 0.052, 95% CI −0.009 to 0.114, p = 0.096).

Two results should be interpreted causally with the qualification stated above, and one should not. The T1 estimates indicate a strong, precisely estimated association between prolonged length-of-stay and both a higher unsuccessful-discharge rate and substantially higher cost; the corresponding E-values (2.53 for the discharge proxy, 3.25 for cost) indicate that an unmeasured confounder would need to be associated with both prolonged stay and the outcome by a risk ratio of roughly 2.5- to 3.2-fold, beyond what the 18 measured covariates already explain, to fully account for these associations. This is a moderately demanding bar, but it is not proof of an absent confounder, and it does not resolve the structural concern that length-of-stay, cost and discharge status are all determined during or after the hospitalisation and are plausibly driven by unmeasured clinical severity (disease stage, comorbidity burden) that is only partially captured by the severity index discussed under Table 6. Accordingly, the T1 results are reported strictly as associational throughout this manuscript and are not used to support causal claims about the effect of reducing length-of-stay. The T2 and T3 estimates, by contrast, concern administratively or contractually assigned exposures and are interpreted, conditional on the observed covariates, as causal effects: high coverage is associated with materially lower cost with no detectable change in unsuccessful discharge, while high transaction intensity shows no statistically significant effect on either outcome in its primary specification. Because the underlying claims data lack a unique patient identifier, repeated admissions from the same patient cannot be linked and may be present as apparently independent observations; the reported standard errors and confidence intervals do not incorporate within-patient clustering, and the true precision of all estimates in this table should be regarded as an upper bound pending patient-level linkage or a cluster-robust sensitivity analysis.

Table 8 summarises out-of-fold predictive performance of the nuisance models underlying the primary DML specifications. The outcome model for the discharge proxy discriminated well across all three treatments (AUC-ROC 0.852–0.854), while the outcome model for log-cost explained 70.7–71.1% of out-of-fold variance (R^2^ = 0.707–0.711) for T1/T2/T3. Propensity-model discrimination was moderate for all three treatments (AUC-ROC 0.690–0.752).

**Table 8:** Cross-fitted nuisance-model performance for the propensity [E(T|X)] and outcome [E(Y|X)] models underlying the primary DML specifications.

Treatment	Nuisance target	Model	AUC-ROC	AUC-PR	Accuracy	F1	Log-loss	Brier	RMSE	MAE	R^2^
T1 — Prolonged LOS	Y2 discharge proxy	E[Y|X]	0.854	0.560	0.900	0.455	0.263	0.076	—	—	—
T1 — Prolonged LOS	Treatment	E[T|X]	0.752	0.678	0.699	0.570	0.569	0.195	—	—	—
T1 — Prolonged LOS	Y3 log-cost	E[Y|X]	—	—	—	—	—	—	0.610	0.487	0.711
T2 — High coverage	Y2 discharge proxy	E[Y|X]	0.854	0.560	0.900	0.455	0.263	0.076	—	—	—
T2 — High coverage	Treatment	E[T|X]	0.690	0.685	0.622	0.629	0.637	0.223	—	—	—
T2 — High coverage	Y3 log-cost	E[Y|X]	—	—	—	—	—	—	0.610	0.487	0.711
T3 — High transaction intensity	Y2 discharge proxy	E[Y|X]	0.852	0.559	0.902	0.462	0.264	0.076	—	—	—
T3 — High transaction intensity	Treatment	E[T|X]	0.720	0.486	0.752	0.302	0.512	0.171	—	—	—
T3 — High transaction intensity	Y3 log-cost	E[Y|X]	—	—	—	—	—	—	0.613	0.488	0.708

Nuisance-model fit was consistently adequate for the purpose of orthogonalized estimation: the outcome models achieved good discrimination for the binary discharge proxy and explained roughly 71% of the variance in log-cost, and the treatment (propensity) models, while more modestly discriminating (AUC-ROC 0.69–0.75), showed no evidence of near-degenerate class separation that would threaten overlap/positivity. The comparatively lower F1 score for the T3 treatment model (0.302) reflects the class imbalance of the high-transaction-intensity exposure rather than poor calibration, and is addressed further via the threshold-robustness analyses in the Supporting Information (S8–S9 Tables in S1 Appendix). As with Table 7, these diagnostics describe model fit conditional on the observed covariates and do not, by themselves, certify that unmeasured confounding has been eliminated.

### Stage 3: Causal effect estimation of length-of-stay, insurance coverage, and treatment intensity on discharge and cost outcomes

*Abbreviations: ATE, average treatment effect; AIPW, augmented inverse-probability weighting; CI, confidence interval (used exclusively in this sense throughout this section); DAG, directed acyclic graph; FW-DML, Frisch–Waugh partialling-out double/debiased machine learning; pp, percentage point; SE, standard error; SES, socioeconomic status; LOS, length of stay. Cost effects are estimated and reported on the natural-log scale and are additionally expressed as proportional (percentage) changes, a currency-invariant transformation. Absolute episode-cost figures reported elsewhere in the manuscript were converted from Iranian Rials (IRR) to purchasing-power-parity–adjusted international dollars (Int$) using period-specific factors of approximately 146,000–150,000 IRR/Int$ for 2024 and 150,000–155,000 IRR/Int$ for 2025; because the estimators below operate on standardized covariates and log-cost, this conversion does not alter any point estimate, standard error, confidence interval, or p-value reported in this section.*

Prior to effect estimation, a directed acyclic graph (DAG) encoding the assumed causal structure of the study was prespecified and validated computationally rather than asserted narratively. The DAG designates 20 variables that are fixed at or before hospital admission (demographic, administrative, physician/service, geographic, temporal, and socioeconomic characteristics) as the exogenous confounder set common to all three exposures and both outcomes, while length of stay, cost per day, total cost, coverage ratio, same-day discharge, adverse discharge, recovery status, and claims-activity counts are explicitly encoded as post-treatment mediators rather than confounders (S10 and S11 Tables in S1 Appendix). Four automated consistency checks were then run against this graph and the adjustment sets actually used by the Step 3 estimators; all four passed with zero violations (Table 9).

**Table 9:** Automated validation of the prespecified causal directed acyclic graph (DAG) and of the adjustment sets used in the Step 3 estimators.

Check	Status	Detail
Graph is acyclic	Pass	No directed cycles detected among the 38 DAG nodes
All declared confounders are exogenous root nodes	Pass	0 of 20 confounders identified as descendants of any treatment or outcome node
No post-treatment or outcome-derived variable is an unacknowledged ancestor of a treatment	Pass	0 violations; all post-treatment quantities (LOS, cost, discharge status, claims activity) are encoded only as mediators/outcomes, never as inputs to the treatment-assignment models
Adjustment sets used in the Step 3 estimators match the prespecified confounder list exactly	Pass	0 discrepancies; all 20 covariates entering the nuisance models for every exposure–outcome pair are confounders declared in the DAG a priori

Validation performed on the full analytic sample (n = 1,813 episodes; dataset fingerprint fe48be240351b89b). The full node inventory and the post-treatment (mediator) edge list underlying this check are reported in S10 and S11 Tables in S1 Appendix.

This prespecified-DAG approach directly constrains the adjustment sets to variables that are fixed at or before the point of treatment assignment; length of stay, cost, discharge status and the components used to build the severity index are excluded from the confounder role and instead modelled as downstream, post-treatment quantities. Because prolonged length of stay (T1) is itself the treatment whose duration mechanically co-determines cost, discharge status, and any index built from them, the graph and the estimates that follow do not treat the T1 comparison as free of endogeneity; instead, this structural constraint is what allows the T1 estimates to be labelled explicitly as associational rather than causal throughout this section (Table 11), while the two exposures that are plausibly determined at or before admission (insurance-coverage tier and claims-intensity) are evaluated under the stated conditional-exchangeability assumption. The DAG check therefore documents what was adjusted for and on what basis, without overstating what the design can identify.

Because the composite severity index (severity_score) is retained in the confounder set, its empirical association with the three post-treatment utilisation quantities from which its components are conceptually related — length of stay, cost per day, and total cost — was screened independently on the full analytic sample (n = 1,813; 52 candidate columns) using a prespecified threshold of |r| = 0.30. All three correlations were small (|r| ≤ 0.078) and the gate returned an overall status of pass, with zero flagged columns (Table 10).

**Table 10:** Independent leakage screening of the composite severity index against post-treatment length-of-stay and cost quantities.

Post-treatment / utilisation quantity	Pearson r with severity index	Prespecified threshold (|r|)	Screening outcome
Length of stay, days	0.064	0.30	Below threshold
Cost per day	−0.078	0.30	Below threshold
Total episode cost	0.074	0.30	Below threshold

Overall gate status: pass (n = 1,813 episodes; 52 columns screened; 0 columns flagged; dataset fingerprint fe48be240351b89b, screened 2026-09-07). Recomputed correlations matched the in-memory values generated at the point of covariate construction, i.e., this is an independent re-verification rather than a repeated internal claim.

The magnitude of these correlations (r ≤ 0.08 in absolute value, an order of magnitude below the 0.30 screening threshold) indicates that the severity index is not simply re-encoding the realised length of stay or cost of the index admission, which is the specific mechanism by which a post-treatment variable would bias the adjustment set. This screen is deliberately narrow: it rules out gross leakage of the realised treatment and its immediate cost consequences into the covariate set, but it cannot, on its own, establish that the index is free of any relationship to unmeasured clinical severity that also drives prolonged stay. For that reason the index is used as an ancillary risk-adjustment covariate alongside 19 unambiguously pre-treatment demographic, administrative, geographic, temporal, and socioeconomic variables, and it is this combination — not the severity index in isolation — that supports the causal-status labelling applied to each exposure in Table 11.

**Table 11:** Primary treatment-effect estimates for prolonged length of stay, high insurance-coverage tier, and continuous treatment intensity on the discharge-risk proxy and log total cost.

Exposure	Outcome	Estimator	n (estimation sample)	Effect	95% CI	p	E-value (point / CI limit)	Causal status
Prolonged LOS (T1)	Unsuccessful-discharge proxy (Y2)^a^	FW-DML	1812	−6.4 pp	−8.6 to −4.1 pp	<0.001	1.68 / 1.49	Associational^b^
Prolonged LOS (T1)	Log total cost (Y3)	FW-DML	1812	+200.2%	+184.3 to +217.1%	<0.001	4.25 / 4.04	Associational^b^
High coverage (T2)	Unsuccessful-discharge proxy (Y2)^a^	AIPW	1813	+2.2 pp	−0.5 to +4.9 pp	0.120	1.32 / 1.00	Causal, conditional exchangeability^c^
High coverage (T2)	Log total cost (Y3)	AIPW	1813	−39.7%	−43.6 to −35.5%	<0.001	2.37 / 2.19	Causal, conditional exchangeability^c^
Treatment intensity (T3)	Unsuccessful-discharge proxy (Y2)^a^	FW-DML	1722	−22.1 pp	−30.0 to −14.2 pp	<0.001	3.13 / 2.35	Exploratory^d^
Treatment intensity (T3)	Log total cost (Y3)	FW-DML	1722	+1,154.6%	+955.7 to +1,390.9%	<0.001	n/aᵉ	Mechanical, non-causal^e^

^a^Y2 is a within-episode proxy for unsuccessful discharge (same-day discharge, non-recovery, or adverse discharge) and is not a linked, longitudinally observed 30-day readmission; it is reported and interpreted as a discharge-risk proxy throughout. ^b^Reported as an associational estimate because length of stay is the treatment itself and is mechanically entangled with the mediators (cost, discharge status) used to construct the ancillary severity covariate; it should not be read as the causal effect of extending a stay. ^c^Reported as a causal estimate under the assumption of conditional exchangeability given the pre-treatment covariate set (Table 9; S10 Table in S1 Appendix), treating coverage tier as a policy/administrative lever; residual confounding from unmeasured clinical severity, disease stage, or comorbidity burden cannot be excluded. ^d^T3 shows weaker first-stage residual variation (S12 Table in S1 Appendix) and is therefore reported as exploratory. ^e^The T3–Y3 pair is a mechanical/accounting association (log claims count and log total cost are linked by billing construction: more transactions mechanically generate more billable line items) and is not interpretable as a behavioural or policy-relevant causal effect; no E-value is reported because the association is not being advanced as causal. All intervals assume independence across the 1,813 episodes; the underlying claims extract carries no patient identifier, so patient-level independence is not assumed but is an explicit, disclosed limitation (S13 Table in S1 Appendix).

Table 11 reports the primary doubly-robust treatment-effect estimates for the three exposures — prolonged length of stay (T1), high insurance-coverage tier (T2), and continuous treatment/claims intensity (T3) — on the unsuccessful-discharge proxy (Y2) and log total cost (Y3), together with the explicit causal-status label attached to each estimate. Prolonged LOS was associated with a 6.4-percentage-point lower rate of unsuccessful discharge (95% CI −8.6 to −4.1 pp, p < 0.001) and a 200% higher total cost (95% CI 184% to 217%, p < 0.001), both explicitly labelled associational. High insurance coverage was associated with a statistically imprecise 2.2-pp change in unsuccessful discharge (95% CI −0.5 to 4.9 pp, p = 0.120) and a 40% lower total cost (95% CI −44% to −36%, p < 0.001), reported as causal estimates under the stated conditional-exchangeability assumption. Continuous treatment intensity (T3) showed an exploratory association of −22 pp with unsuccessful discharge, while its association with log cost (point estimate corresponding to an implausible >1,000% increase) is flagged as mechanical and explicitly excluded from causal or policy interpretation.

Framed this way, the three exposures occupy three distinct positions on the causal-inference spectrum rather than being pooled under a single causal narrative. The E-values attached to the two estimates carried forward as causal or exploratory (T1 and T2) indicate that an unmeasured confounder would need to be associated with both the exposure and the outcome by a risk ratio of roughly 1.3 to 4.3 (point estimate) — and no less than 1.0 to 4.0 at the confidence bound — to fully explain away the corresponding estimate; this is reported as a quantitative bound on, not a resolution of, residual confounding from the clinical variables (disease stage, comorbidity burden, medication regimen) that were not observed in these claims data. The discharge-risk outcome is reported throughout as a within-episode proxy rather than as observed rehospitalisation, and the T3–cost pairing is retained in the table for transparency but is explicitly quarantined from causal or policy interpretation because it reflects the accounting structure of claims billing rather than a clinical or behavioural mechanism.

To assess whether the two estimates carried forward for interpretation are stable across clinically and administratively meaningful strata, Table 12 reports subgroup average treatment effects for prolonged LOS on the discharge-risk proxy and for high coverage on log cost across age band, severity tier, hospital sector, rurality, and socioeconomic status. The direction and approximate magnitude of both associations were consistent across all ten subgroups; the private-hospital subgroup (n = 100) showed a materially smaller point estimate for both outcomes and is flagged as low precision given its small sample size and correspondingly wide interval.

**Table 12:** Subgroup treatment-effect estimates for prolonged length of stay on the discharge-risk proxy (Y2) and for high insurance coverage on log total cost (Y3).

Subgroup	n	T1 → Y2 ATE, pp (95% CI)	T2 → Y3 Δ cost, % (95% CI)
Age < 65	489	−8.6 pp (−13.0, −4.2)	−38.6% (−45.9, −29.4)
Age 65–74	567	−4.3 pp (−8.1, −0.6)	−37.4% (−44.4, −27.6)
Age ≥ 75	757	−6.4 pp (−10.1, −2.8)	−42.1% (−48.1, −35.9)
Low clinical severity	871	−7.6 pp (−10.9, −4.3)	−38.7% (−44.3, −32.4)
High clinical severity	942	−5.3 pp (−8.4, −2.1)	−40.7% (−46.3, −34.0)
Private hospital^a^	100	−6.6 pp (−13.1, −0.1)	−22.6% (−37.1, −3.1)
Public hospital	1713	−6.4 pp (−8.7, −4.0)	−40.6% (−45.0, −35.9)
Rural	547	−6.3 pp (−9.9, −2.7)	−40.1% (−48.1, −29.9)
Urban	1266	−6.4 pp (−9.3, −3.5)	−39.6% (−44.2, −34.6)
High SES^b^	1813	−6.4 pp (−8.6, −4.1)	−39.7% (−43.6, −35.5)

^a^Low-precision subgroup (small n and/or wide interval relative to the point estimate); interpret cautiously. ^b^The low-SES stratum contained 0 episodes above the n ≥ 30 analysis threshold in this extract and is omitted; percentage change in cost computed as (e^b^ − 1) × 100 from the log-scale coefficient. Full subgroup results for all six exposure–outcome pairs are reported in S14–S16 Tables in S1 Appendix.

The consistency of sign and approximate magnitude across age, severity, sector, geography, and SES strata is evidence against a single dominant confounder or a single subgroup driving the pooled estimate, particularly for the coverage–cost pairing, which is the estimate advanced as causal. The narrower and sometimes attenuated effect in the small private-hospital stratum illustrates why subgroup estimates with wide intervals are flagged rather than treated as evidence of genuine effect-measure modification; formal tests of heterogeneity (best-linear-predictor and rank-correlation diagnostics) are reported alongside the full nuisance-model diagnostics in S13 Table in S1 Appendix.

### Stage 4: Socioeconomic-related inequality in discharge, cost, and financial-protection outcomes

*Abbreviations added in this section: C, concentration index (reserved exclusively for the income/socioeconomic-related inequality index defined below; CI continues to denote confidence interval only, as in Tables 9–12, so that the two quantities are never denoted by the same symbol); HI, horizontal inequity index (need-standardised concentration index); LPM, linear probability model; OOP, out-of-pocket (payment).*

This step is a descriptive analysis of socioeconomic-related distributional gradients — concentration indices, horizontal-inequity indices, and a linear-decomposition of each concentration index into the contributions of its correlates — computed on the same 1,813-episode analytic sample used in Step 3. It does not use the T1–T3 exposure definitions, and none of the quantities reported below is a doubly-robust or double/debiased causal estimate; the concentration index C is a rank-correlation-based summary of how an outcome is distributed across the socioeconomic ranking, not a treatment effect, and the causal-identification issues that motivate the associational/causal/exploratory labelling of the exposures in Table 11 therefore do not apply mechanically to the results in this section. The composite severity index is used here only as one candidate covariate in a “need” set used to compute the horizontal-inequity index, and its role is tested directly by recomputing every horizontal-inequity estimate with the severity index dropped from the need set entirely (Table 14); this is a full removal, not a sensitivity analysis that keeps the index in a modified form.

Table 13 reports the concentration index (CI) of each of the five health and financial-protection outcomes with respect to three candidate socioeconomic ranking variables. Using insurance-fund type as the primary ranking variable, recovery (Y1) was concentrated among higher-SES episodes (C = +0.087, 95% CI 0.048 to 0.129, p < 0.001), whereas log total cost (Y3), out-of-pocket ratio (Y4), and length of stay (Y5) were all significantly concentrated among lower-SES episodes (C = −0.006, −0.056, and −0.046 respectively, all p ≤ 0.001). The unsuccessful-discharge proxy (Y2) showed a small, non-significant pro-poor gradient on the primary ranking variable (C = −0.022, 95% CI −0.056 to 0.011, p = 0.209).

**Table 13:** Concentration indices (CI) for five health and financial-protection outcomes, by socioeconomic ranking variable.

Outcome (n = 1,813)	C, insurance fund type^a^ (95% CI)	C, composite SES rank (95% CI)	C, rural residence (95% CI)
Recovery (Y1)b	+0.087 (0.048, 0.129)***	+0.053 (0.012, 0.094)*	+0.062 (0.030, 0.096)***
Unsuccessful-discharge proxy (Y2)^bc^	−0.022 (−0.056, 0.011)	+0.004 (−0.031, 0.040)^d^	−0.029 (−0.054, −0.004)*
Log total cost (Y3)	−0.0064 (−0.0081, −0.0046)***	−0.0045 (−0.0063, −0.0027)***^d^	−0.0035 (−0.0047, −0.0022)***ᵈ
Out-of-pocket ratio (Y4)	−0.056 (−0.077, −0.035)***	−0.043 (−0.063, −0.023)***	−0.060 (−0.076, −0.044)***
Length of stay, days (Y5)	−0.046 (−0.073, −0.017)**	−0.027 (−0.057, 0.001)	−0.036 (−0.056, −0.012)**

*p < 0.05, **p < 0.01, ***p < 0.001. Positive C indicates concentration among higher-socioeconomic-status episodes (pro-rich); negative C indicates concentration among lower-socioeconomic-status episodes (pro-poor). ^a^Primary socioeconomic ranking variable for this analysis; the composite SES rank and rural-residence indicator are reported as robustness checks. ^b^Recovery and the discharge-risk proxy are binary outcomes; C is computed with the Erreygers-corrected concentration index, appropriate for bounded outcomes, whereas the three continuous outcomes use the standard concentration index. ^c^Y2 is a within-episode proxy for unsuccessful discharge (same-day discharge, non-recovery, or adverse discharge), not a linked, longitudinally observed 30-day readmission; it is labelled and interpreted as a discharge-risk proxy throughout, consistent with Table 11. ^d^Estimate flagged as near-zero in absolute magnitude notwithstanding statistical significance at this sample size; interpret the substantive gradient, not only its significance.

The pattern across outcomes is internally consistent with a single underlying story: episodes at the higher-socioeconomic end of the insurance-fund ranking are more likely to be recorded as recovered, and are systematically associated with lower cost, lower out-of-pocket burden, and shorter stays, than lower-socioeconomic episodes. The one outcome without a significant gradient on the primary ranking variable — the discharge-risk proxy — is also the outcome for which the decomposition below is classified as unreliable (Table 15), so its concentration index is reported for completeness but is not used to support any equity narrative. Agreement in sign and approximate magnitude across three conceptually distinct socioeconomic proxies (an administrative insurance-fund classification, a composite rank score, and a geographic rurality indicator) reduces the chance that the pattern is an artefact of any one proxy’s construction.

Because the need-standardisation set used to compute the horizontal-inequity index optionally includes the composite severity score, every horizontal-inequity estimate was recomputed with the severity score dropped from the need set entirely. Table 14 reports both versions side by side. The horizontal-inequity estimate changed by at most 0.0003 in absolute terms across all five outcomes, and no outcome changed statistical significance or direction when the severity score was removed.

**Table 14:** Horizontal-inequity index (need-standardised concentration index) for insurance-fund type, with and without the composite severity score in the need-standardisation set.

Outcome	HI, full need set^a^ (95% CI), p	HI, severity score excluded (95% CI), p	Δ HI	Conclusion
Recovery (Y1)	+0.046 (0.009, 0.085), p = 0.028	+0.046 (0.008, 0.084), p = 0.027	−0.0002	Robust
Unsuccessful-discharge proxy (Y2)	−0.026 (−0.059, 0.007), p = 0.126	−0.026 (−0.060, 0.007), p = 0.129	−0.0001	Robust
Log total cost (Y3)	−0.0051 (−0.0068, −0.0034), p < 0.001	−0.0051 (−0.0067, −0.0034), p < 0.001	+0.00003	Robust
Out-of-pocket ratio (Y4)	−0.061 (−0.081, −0.041), p < 0.001	−0.061 (−0.081, −0.041), p < 0.001	+0.0003	Robust
Length of stay (Y5)	−0.033 (−0.059, −0.005), p = 0.017	−0.033 (−0.059, −0.004), p = 0.018	−0.0002	Robust

^a^Full need set = age + sex + composite severity score; severity-excluded need set = age + sex only. Horizontal inequity is the concentration index of the need-standardised outcome (observed outcome minus its need-predicted value) with respect to insurance-fund type; Δ HI = HI(severity excluded) − HI(full need set). “Robust” indicates a change in HI of ≤0.0003 in absolute terms with no reversal of statistical significance or sign in this dataset.

This complete-removal check speaks directly to the concern that the severity index could be standing in for the treatment or its consequences rather than for genuine clinical need: if the pro-rich or pro-poor horizontal-inequity conclusions in Table 14 depended on the severity index, removing it altogether would be expected to shift the point estimates and, in the two outcomes with the least precise intervals (Y1, Y2), plausibly to flip statistical significance. Neither occurred for any of the five outcomes. This is evidence against the severity index being the source of the equity findings reported here; it is not, on its own, proof that the index carries no information about post-treatment severity, and the index is not used at all in the exposure or outcome models that produce the causal and associational estimates of Table 11, which rest on the separately validated pre-treatment confounder set of Table 9.

Each concentration index in Table 13 was further decomposed, following a linear-probability-model Wagstaff–van Doorslaer decomposition, into the portion attributable to five observed correlates (sex, age, hospital ownership, admission season, and the severity score) and a residual term capturing everything the linear model does not explain. Because a decomposition is only informative when the residual does not dominate the total, every outcome was assigned a reliability tier based on the ratio of the residual contribution to the total concentration index, and only outcomes classified reliable or borderline are given a determinant-level narrative in Table 15; the discharge-risk proxy is classified unreliable and its decomposition is reported as such rather than narrated.

**Table 15:** Reliability-tiered decomposition of the concentration index (CI) for insurance-fund type, by outcome.

Outcome	Reliability tier^a^	Residual share of C, %^b^	Leading determinant (share of C)	2nd determinant (share of C)	3rd determinant (share of C)
Recovery (Y1)	Reliable	67.8%	Age (+42.4%)	Private hospital (−15.7%)	Severity score (+6.2%)
Unsuccessful-discharge proxy (Y2)	Unreliable	148.4%	Not reported^c^	Not reported^c^	Not reported^c^
Log total cost (Y3)	Reliable	62.5%	Age (+19.7%)	Private hospital (+17.8%)	Severity score (−0.8%)
Out-of-pocket ratio (Y4)	Borderline	97.3%	Private hospital (indicative)^d^	Age (indicative)^d^	Severity score (indicative)^d^
Length of stay (Y5)	Reliable	67.9%	Age (+26.0%)	Private hospital (+3.1%)	Severity score (+3.1%)

^a^Reliable: residual share < 80% of the total concentration index; borderline: 80–100%; unreliable: > 100% (the residual exceeds and can reverse the sign of the observed index). Rural residence was excluded from every decomposition as a determinant because of its strong collinearity with the primary ranking variable, insurance-fund type (ρ = 0.63; S19 Table in S1 Appendix). ^b^|residual contribution| / |observed concentration index|, expressed as a percentage. ^c^For the discharge-risk proxy, the residual exceeds the total observed concentration index, so the five included correlates do not meaningfully explain this outcome’s gradient; no determinant-level contributions are reported and none should be inferred from the raw decomposition figures in S20 Table in S1 Appendix. ^d^For the out-of-pocket ratio, contributions are reported in S20 Table in S1 Appendix as approximate/indicative only, consistent with its borderline reliability tier, and percentage shares are not computed.

Reporting a reliability tier alongside every decomposition, and withholding a determinant-level narrative for the tier in which the residual dominates, is intended to prevent over-interpretation of a linear decomposition applied to a discrete or noisy outcome. Where the decomposition is reliable (recovery, cost, length of stay), age and hospital ownership consistently emerge as the two largest observed correlates of the socioeconomic gradient, with the severity score itself contributing a materially smaller and, for cost, a near-zero and sign-mixed share (−0.8% to +6.2% across the three reliable outcomes) — the opposite of what would be expected if the severity index, rather than age and hospital sector, were driving the reported equity gradients. Full determinant-by-determinant figures for all five outcomes, including the unreliable and borderline ones, are given in S20 Table in S1 Appendix for transparency.

### Stage 5: Constrained welfare optimisation

Building on the causal effect estimates obtained after restricting the adjustment set to pre-treatment variables, Stage 5 translates the estimated treatment effects into a set of model-based counterfactual allocation scenarios rather than an empirically implemented policy. Expanded insurance coverage (T2_high_coverage) is treated throughout as the PRIMARY policy-relevant scenario, because coverage level is an insurer-assignable, pre-admission lever. Prolonged length of stay (T1_prolonged_los) is retained only as an EXPLORATORY, descriptive comparator: because a prolonged stay is realised during the hospitalisation itself and plausibly reflects clinical severity and in-hospital complications rather than a lever that can be assigned in advance, T1 results in Tables 16-20 describe patterns among patients who experienced a prolonged stay and are not used to derive allocation recommendations. All monetary quantities are converted from the original Iranian rial (IRR) claims values to purchasing-power-parity-adjusted international dollars (Int$) using an approximate factor of 150,000 IRR per Int$, within the ~ 146,000–155,000 IRR/Int$ range estimated for the 2024–2025 study period; because this factor is itself an approximation, converted figures should be read as indicative order-of-magnitude quantities rather than exact currency conversions.

**Table 16:** Base-case constrained allocation under the significance-gated welfare objective (α = 1.0, λ = 1.0).

Scenario (treatment)	Role	Solver / status	N treated	% treated	Welfare index (W)	Bed-day budget used (%)	Insurer-cost budget used (%)
T1_prolonged_los	Exploratory	ILP/CBC (optimal)	495	27.3	487.34	14.0	100.0
T2_high_coverage	Primary	ILP/CBC (optimal)	649	35.8	290.69	38.9	100.0

*W is a dimensionless welfare index combining a standardized health-gain term and an out-of-pocket cost term at the stated α/λ weights; it is not a monetary quantity. Observed system budget: 9,868.0 bed-days; Int$91,543 insurer cost (see* S23 Table in S1 Appendix*).*

*Abbreviations used in Tables 16-20 and S21-S23 Tables in S1 Appendix: CI, 95% confidence interval; CIndex, income-related concentration index of allocation (distinct from CI); ILP, integer linear program; Int$, purchasing-power-parity-adjusted international dollar; IRR, Iranian rial; LOS, length of stay; OOP, out-of-pocket patient cost share; pp, percentage points; SG, significance-gated; W, dimensionless welfare index.*

Table 16 summarises the base-case constrained allocation obtained under the observed system budget (9,868.0 bed-days; Int$91,543 insurer cost) and the significance-gated welfare objective defined in Table 19, at equal base weights (α = 1.0, λ = 1.0). Under this specification, 495 of 1,813 patients (27.3%) are allocated in the exploratory T1 scenario (welfare index W = 487.34) and 649 of 1,813 patients (35.8%) in the primary T2 scenario (W = 290.69); both scenarios exhaust the available insurer-cost budget (100.0%) while using a minority of the bed-day budget (14.0% and 38.9%, respectively).

Because W and the reported budget-utilisation percentages describe what an optimiser selects under the stated assumptions, weights, and observed budget, Table 16 should be read as a model-based scenario description rather than a forecast of the health or cost consequences of implementation; provider, insurer, and patient behaviour could all change realised effects once any allocation is put into practice. The exploratory T1 row is reported strictly as a within-sample descriptive benchmark – i.e., “among patients who experienced a prolonged stay, the following allocation pattern would emerge under these weights” – and is not intended to imply that length of stay can, or should, be assigned as an intervention.

Table 17 reports the equity properties of the same base-case allocation using age (≥75 years, a pre-treatment demographic fact) as the primary protected variable, together with a secondary, exploratory comparison based on the composite severity score described previously. Allocation to the primary T2 scenario is identical across age strata (35.8% in both groups; EQUAL, constraint-bound), while the exploratory T1 scenario shows a small emergent excess toward elderly patients (27.7% vs 27.0%, n = 757 vs n = 1,056). Both scenarios show a modestly pro-rich income-related concentration index (CIndex = 0.081–0.103) and a rural-urban gap of 4.4–8.7 percentage points.

**Table 17:** Equity properties of the base-case allocation, by treatment scenario.

Scenario (treatment)	Role	Age-equity classification (≥75y, primary)	% treated, elderly (n)	% treated, other (n)	Rural- urban gap (pp)	CIndex (allocation)	Allocation direction	Severity-score classification (secondary, exploratory)
T1_prolonged_los	Exploratory	FAVOURS_HIGH (emergent)	27.7 (n = 757)	27.0 (n = 1056)	4.4	0.081	pro-rich	FAVOURS_LOW (inverted)
T2_high_coverage	Primary	EQUAL (constraint- bound)	35.8 (n = 757)	35.8 (n = 1056)	8.7	0.103	pro-rich	FAVOURS_LOW (inverted)

CIndex, income-related concentration index of the allocation (values above 0 indicate allocation concentrated among wealthier patients); this index is unrelated to the 95% confidence interval (CI) reported elsewhere. n denotes the number of patients in the corresponding age stratum (elderly, ≥ 75y, n = 757; other, n = 1,056), out of N = 1,813.

The primary equity guarantee is defined using age, a fact recorded prior to and independent of treatment, rather than the composite severity score, since that score is constructed in part from within-admission length of stay, cost-per-day, and discharge status and is therefore not a pre-treatment variable. The severity-score-based classification is reported only as a secondary, exploratory comparison: its FAVOURS_LOW (inverted) classification for both scenarios describes the relationship between allocation and an outcome-adjacent index, and should not be read as evidence bearing on the primary, age-based equity guarantee, nor as a validated statement about targeting of clinically severe patients in the absence of validated clinical severity measures (e.g., disease stage or comorbidity burden) in the underlying claims data.

Table 18 compares observed practice with two counterfactual allocation scenarios per treatment – a welfare-optimal, age-equity-constrained scenario (Scenario B) and an equity-maximising, out-of-pocket-priority scenario (Scenario C) – expressed using the discharge-success rate (one minus the modelled probability of an unsuccessful discharge). Relative to observed practice (88.09% discharge success), the welfare-optimal scenario is associated with a projected 2.9–4.1 percentage-point gain in discharge success while treating a smaller share of patients (27.3–35.8% vs 40.2–50.0% observed), and the equity-maximising scenario prioritises out-of-pocket reduction (up to 6.8% mean reduction) for a much smaller group (3.0–3.5%).

**Table 18:** Counterfactual comparison of observed practice with welfare-optimal and equity-maximising allocation scenarios.

Scenario (treatment)	Role	Allocation scenario	Discharge success, observed (%)	Discharge success, projected (%)	Change (pp)	N treated	Mean OOP, observed (Int$)	Mean OOP, projected (Int$)	OOP reduction (%)
T1_prolonged_los	Exploratory	A. Observed practice	88.09	71.18	−16.9	728	50.5	48.7	3.6
T1_prolonged_los	Exploratory	B. Welfare-optimal (age-equity-constrained)	88.09	90.98	2.9	495	50.5	50.0	1.1
T1_prolonged_los	Exploratory	C. Equity-maximising (OOP-priority)	88.09	86.80	−1.3	54	50.5	49.1	2.8
T2_high_coverage	Primary	A. Observed practice	88.09	91.19	3.1	906	50.5	48.3	4.3
T2_high_coverage	Primary	B. Welfare-optimal (age-equity-constrained)	88.09	92.16	4.1	649	50.5	50.0	0.9
T2_high_coverage	Primary	C. Equity-maximising (OOP-priority)	88.09	88.14	0.1	63	50.5	47.1	6.8

Discharge success is derived entirely within the same-admission discharge-status outcome space (1 – modelled unsuccessful-discharge probability); it does not represent a subsequently observed 30-day hospital readmission, since no longitudinal linkage to later admissions was available in the claims extract used for this study. OOP, mean out-of-pocket patient cost share, converted to Int$ at 150,000 IRR/Int$ (see manuscript text).

These figures are modelled counterfactual comparisons, conditional on the estimated treatment effects and the stated welfare weights, and should not be interpreted as the discharge outcomes that would be observed after implementing any specific reallocation in practice; residual confounding from clinical variables not captured in the claims data (e.g., validated disease stage, comorbidity burden, medication regimens) remains plausible and is not resolved by the adjustment for observed pre-treatment covariates alone. Consistent with this, the outcome is labelled and interpreted throughout as a discharge-success/discharge-risk proxy rather than as a reduction in readmission.

Table 19 documents, for each treatment-outcome pair, whether the corresponding 95% CI excludes zero and is therefore permitted to enter the welfare objective used in Tables 16, 18 and 20, in place of a manually tuned relative weight between competing causal terms.

**Table 19:** Significance-gating decisions determining which causal effects enter the welfare objective.

Scenario (treatment)	Outcome	95% CI	Included in welfare objective
T1_prolonged_los	Y2, unsuccessful-discharge proxy	−0.0864, −0.0410	Yes
T1_prolonged_los	Y3, log(total cost)	1.0447, 1.1541	Yes
T2_high_coverage	Y2, unsuccessful-discharge proxy	−0.0050, 0.0488	No
T2_high_coverage	Y3, log(total cost)	−0.5721, −0.4379	Yes

CI, 95% confidence interval on the corresponding causal effect estimate. An effect is included only if its CI excludes zero; when both effects for a treatment are included, they are combined via an equal-weight, standardized blend (see Table 20 and manuscript text).

Under this rule, both effects for the exploratory T1 scenario clear the threshold and are combined via an equal-weight standardized blend of two effects that are each independently distinguishable from zero. For the primary T2 scenario, only the cost effect (Y3) clears the threshold; the discharge-proxy effect (Y2) is excluded because its CI crosses zero, rather than being blended into the objective at a fixed 50/50 weight, so that an effect that is statistically indistinguishable from no effect cannot drive a share of the estimated allocation.

Table 20 compares the base-case allocation obtained under three alternative welfare-objective specifications: restricted to the discharge-proxy effect only (Y2_ONLY), an unweighted blend of both estimated effects regardless of statistical significance (MULTI_OUTCOME), and the significance-gated specification defined in Table 19 (SIGNIFICANCE_GATED, used throughout Tables 16-18). Under Y2_ONLY, the primary T2 scenario allocates zero patients; MULTI_OUTCOME and SIGNIFICANCE_GATED both allocate 495 patients (27.3%) to the exploratory T1 scenario, and 460 (25.4%) and 649 (35.8%) patients respectively to the primary T2 scenario.

**Table 20:** Comparison of allocation under three alternative welfare-objective specifications.

Objective mode	Scenario (treatment)	Role	N treated	% treated	Welfare index (W)	Age-equity classification	Zero- allocation
Y2_ONLY	T1_prolonged_los	Exploratory	752	41.5	45.52	EQUAL (constraint-bound)	No
Y2_ONLY	T2_high_coverage	Primary	0	0.0	0.00	N/A	Yes
MULTI_OUTCOME	T1_prolonged_los	Exploratory	495	27.3	487.34	FAVOURS_HIGH (emergent)	No
MULTI_OUTCOME	T2_high_coverage	Primary	460	25.4	71.08	FAVOURS_HIGH (emergent)	No
SIGNIFICANCE_GATED	T1_prolonged_los	Exploratory	495	27.3	487.34	FAVOURS_HIGH (emergent)	No
SIGNIFICANCE_GATED	T2_high_coverage	Primary	649	35.8	290.69	EQUAL (constraint-bound)	No

Zero-allocation indicates that the optimiser selected no patients for treatment under that objective specification. Under Y2_ONLY, the mean modelled health gain for T2_high_coverage is negative for 99.98% of patients, reflecting the underlying (statistically null) causal estimate rather than a solver error.

The zero-allocation result under Y2_ONLY is a direct, mechanical consequence of using a single, statistically null causal effect as the sole benefit term for the primary scenario; it does not indicate a computational fault. MULTI_OUTCOME resolves the zero-allocation outcome but does so by combining a statistically robust cost effect with a null discharge-proxy effect at an equal, unjustified weight. SIGNIFICANCE_GATED is adopted as the reported specification throughout this manuscript because it is the only one of the three that avoids both problems without requiring a manually tuned weight: it excludes the statistically null term for T2 entirely (Table 19) rather than assigning it either a zero implicit weight (Y2_ONLY, which discards the scenario) or an arbitrary equal weight (MULTI_OUTCOME).

### Stage 6: Multi-layer uncertainty quantification

Point estimates from the causal and welfare models are subject to sampling variability, unmeasured confounding, and specification choices; each of these sources of uncertainty was therefore quantified separately, using 1,000 bootstrap replications for the causal, subgroup, and equity estimates and 500 replications for the welfare-optimisation estimates (Table 21). Throughout this section, the length-of-stay-based exposure (T1) is reported as an exploratory association rather than a causal effect, because it is derived from the same hospitalisation episode as the outcomes and no validated pre-treatment clinical severity measure (e.g., Hoehn–Yahr stage, UPDRS score) was available to support an ignorability assumption; the insurance-coverage exposure (T2) is treated as the primary causal estimand; and the continuous claims-volume exposure (T3) is reported as a secondary, descriptive comparison. The constructed outcome Y2 is labelled the *Unsuccessful-Discharge Proxy* throughout, to distinguish it from an observed 30-day hospital readmission, which could not be constructed from these cross-sectional claims data.

**Table 21:** Bootstrap average treatment effects on the discharge-outcome proxy and on total treatment cost, by exposure definition.

Exposure (causal role)	Outcome	Estimator	N	ATE	95% CI	95% CI excludes 0	Approx. % change in cost (95% CI)ᵈ
Prolonged length of stay (exploratory)^a^	Unsuccessful-Discharge Proxy (Y2)^b^	FW-DML	1,812	−0.064	−0.086 to −0.041	Yes	—
Prolonged length of stay (exploratory)^a^	Log total treatment cost (Y3)	FW-DML	1,812	+1.099	1.045 to 1.154	Yes	+200% (+184% to +217%)
High insurance coverage (primary)	Unsuccessful-Discharge Proxy (Y2)^b^	AIPW	1,813	+0.022	−0.005 to 0.049	No	—
High insurance coverage (primary)	Log total treatment cost (Y3)	AIPW	1,813	−0.507	−0.572 to −0.438	Yes	−40% (−44% to −35%)
Continuous claims exposure (secondary)^c^	Unsuccessful-Discharge Proxy (Y2)^b^	FW-DML	1,722	−0.221	−0.300 to −0.142	Yes	—
Continuous claims exposure (secondary)^c^	Log total treatment cost (Y3)	FW-DML	1,722	+2.529	2.357 to 2.702	Yes	+1,155% (+955% to +1,392%)

^a^Length of stay above the service-group median is a within-episode measure realised during, not before, the hospitalisation; it is reported as an exploratory association because it cannot be assumed to satisfy conditional ignorability with respect to unobserved clinical severity, and it is not interpreted as an assignable intervention. ^b^ Y2 is a proxy constructed from same-day discharge, recovery status, and adverse-discharge flags observed within the index episode; it is not a linked, longitudinally observed hospital readmission, and effect estimates on this outcome should be read as changes in the probability of an unsuccessful discharge from the index admission, not as changes in readmission risk. ^c^ Reported for completeness; not used to support any causal or policy conclusion. ^d^ Computed as (exp(ATE) − 1) × 100, appropriate because Y3 is modelled on the natural-log scale; the underlying point estimate and 95% CI (columns 5–6) are the primary reported quantities.

Abbreviations used in Tables 21–23 and S24–S27 Tables in S1 Appendix: ATE, average treatment effect; AIPW, augmented inverse-probability weighting; FW-DML, Frisch–Waugh double/debiased machine learning; 95% CI, 95% confidence interval (bootstrap, bias-corrected, unless stated otherwise); CIndex, concentration index (kept distinct from CI to avoid ambiguity); BCa, bias-corrected and accelerated bootstrap; RR, risk ratio; SE, standard error; ILP, integer linear programming; Int$, PPP-adjusted international dollar; IRR, Iranian rial; pp, percentage points; n/a, not applicable.

Average treatment effects for the discharge-outcome proxy and for the natural-log of total treatment cost, together with their 95% bootstrap confidence intervals, are reported for all three exposure definitions in Table 21.

The only exposure for which both the point estimate and its 95% CI are consistent with a protective association on the discharge-outcome proxy *and* satisfy the pre-treatment-exposure requirement is the insurance-coverage contrast; the length-of-stay and continuous-exposure estimates, while numerically large, are reported strictly as descriptive associations because the exposures on which they are conditioned are themselves determined during or after the episode in which the outcomes are measured. For the primary exposure, high insurance coverage was not associated with a statistically significant change in the probability of an unsuccessful discharge (95% CI spans zero) but was associated with an approximately 40% lower total treatment cost, a result unaffected by the interpretive caveats attached to the exploratory exposure.

To evaluate how sensitive the estimates in Table 21 are to a possible unmeasured confounder, E-value sensitivity statistics were computed for each exposure–outcome pair (Table 22).

**Table 22:** E-value sensitivity of the average treatment effects to unmeasured confounding.

Exposure (causal role)	Outcome	Observed ATE (95% CI)	95% CI crosses the null	Approx. RR (point)	Approx. RR (bound closest to null)	E-value (point)	E-value (limit closest to null)
Prolonged length of stay (exploratory)	Unsuccessful-Discharge Proxy (Y2)	−0.064 (−0.086 to −0.041)	No	0.64	0.77	2.50	1.93
Prolonged length of stay (exploratory)	Log total treatment cost (Y3)	+1.099 (1.045 to 1.154)	No	2.41	2.31	4.26	4.04
High insurance coverage (primary)	Unsuccessful-Discharge Proxy (Y2)	+0.022 (−0.005 to 0.049)	Yes	1.19	1.00	1.67	1.00
High insurance coverage (primary)	Log total treatment cost (Y3)	−0.507 (−0.572 to −0.438)	No	0.67	0.70	2.37	2.19
Continuous claims exposure (secondary)	Unsuccessful-Discharge Proxy (Y2)	−0.221 (−0.300 to −0.142)	No	n/aᵉ	n/aᵉ	n/aᵉ	n/aᵉ
Continuous claims exposure (secondary)	Log total treatment cost (Y3)	+2.529 (2.357 to 2.702)	No	7.57	6.60	14.63	12.67

The E-value is the minimum risk-ratio-scale association that an unmeasured confounder would need with both exposure and outcome, over and above all measured covariates, to fully explain away the observed effect (point E-value) or to shift the 95% CI to include the null (limit E-value); larger values indicate a result that is more robust to unmeasured confounding, and an E-value of 1 indicates that no confounding at all would be required to nullify the result. ᵉ Not estimable: the risk-difference-to-risk-ratio conversion used here requires a defined control-arm baseline risk, which could not be recovered for this continuous, non-binary exposure specification; the risk-difference estimate in column 3 should be interpreted on its own scale, without an accompanying confounding-strength benchmark.

The primary exposure–outcome pair with a statistically significant effect (high insurance coverage → total cost) has an E-value limit of 2.19, indicating that a moderately strong unmeasured confounder would be required to overturn the finding; the corresponding discharge-outcome result is already null, so its E-value limit is 1 by construction and requires no confounding to explain. By contrast, one of the two exploratory length-of-stay models has an E-value limit below 2 (1.93), meaning a comparatively weak unmeasured confounder could account for the association — a pattern consistent with treating an exposure that is partly determined by its own outcome as hypothesis-generating rather than as evidence of a causal effect, and consistent with the exploratory labelling applied to that exposure throughout.

Uncertainty in the downstream welfare-optimisation results was propagated using the same bootstrap resampling scheme; base-case values use the exact integer-linear-programming (ILP) solution, and 95% credible intervals characterise variability across bootstrap replicates (Table 23). Consistent with the exploratory status of the length-of-stay exposure, only an age-based (age ≥ 75 years) targeting ratio is reported as a primary equity metric; a severity-score-based targeting ratio is reported alongside it for completeness but is explicitly exploratory, because the severity score used to construct it combines length of stay, cost per day, and adverse-discharge status — quantities that are themselves realised during or after the treatment window and are therefore not a valid pre-treatment equity stratifier.

**Table 23:** Welfare-optimisation estimates and 95% bootstrap credible intervals for the exploratory and primary reallocation scenarios.

Exposure (causal role)	Optimisation objective	Base welfare, W (95% CI)	% of cohort treated, base (95% CI)	Total health gain, base (95% CI)	Age ≥ 75 targeting ratio — primary equity metric, base (95% CI)	Severity-score targeting ratio — exploratory, base (95% CI)^f^
Prolonged length of stay (exploratory)	Both-outcomes-significant blend	487.34 (469.92 to 504.25)	27.3% (25.8% to 28.9%)	524.42 (505.66 to 543.94)	1.028 (0.919 to 1.164)	0.00 (−0.059 to 0.057)
High insurance coverage (primary)	Cost outcome only	290.69 (280.92 to 300.09)	35.8% (34.7% to 36.9%)	323.63 (313.45 to 333.21)	1.000 (0.968 to 1.075)	0.927 (0.837 to 1.028)

^f^Severity-score targeting ratio caveat: the severity score combines length-of-stay, cost-per-day, and adverse-discharge percentiles, all plausibly downstream of treatment; this column is exploratory only and is not used to support any equity conclusion. The age-based ratio (column 6) is the equity metric on which the primary interpretation of targeting performance is based.

For the primary exposure, the reallocation scenario treats an estimated 35.8% of the cohort (95% CI 34.7–36.9%), with an age ≥ 75 targeting ratio close to 1.0 (95% CI 0.968–1.075), indicating that the optimised allocation does not systematically over- or under-target older patients relative to their share of eligible episodes. These optimisation results describe the welfare achievable under a model-based counterfactual reallocation and should not be read as an empirically demonstrated policy effect, since realised provider, insurer, and patient responses to any actual reallocation were not observed in these data.

### Sensitivity to within-patient dependence

No patient identifier was available in the source claims data, so the 1,813 analysed episodes could not be definitively linked to unique patients; some episodes may represent repeat admissions from the same individual, which would violate the independence assumption underlying the reported standard errors, 95% confidence intervals, and the downstream welfare optimisation. Because true patient-level clustering could not be recovered from the available fields, its potential impact was instead bounded using a design-effect framework: for an assumed average within-patient cluster size, *m*, and intraclass correlation, ICC, the clustered variance is approximated as DEFF ≈ 1 + (*m* − 1) × ICC times the naive variance (Kish, 1965). *m* was swept over {1.0, 1.1, 1.25, 1.5, 2.0, 3.0} and ICC over {0.00, 0.01, 0.02, 0.05, 0.10, 0.15, 0.20, 0.30, 0.50} for every one of the 66 average treatment effects reported in the primary uncertainty-quantification analysis (6 main models plus 60 subgroup models), and the breaking-point ICC — the weakest within-patient correlation at which a given result would lose statistical significance at α = 0.05 — was recorded for each. As a loose contextual anchor only, episodes grouped on coarse demographic fields (age, sex, insurance fund, rural residence) contained an average of 3.45 episodes per group, with 88.6% of episodes falling in a group of size greater than one; because most episodes sharing only these coarse fields are almost certainly different patients rather than repeat visits by the same patient, this figure is reported strictly as an upper bound on plausible values of *m*, not as an estimate of the true repeat-admission rate.

Breaking-point results for the six treatment-role-labelled main average treatment effects are reported in Table 24.

**Table 24:** Design-effect sensitivity of the primary, exploratory, and secondary average treatment effects to within-patient dependence.

Exposure (causal role)	Outcome	N	Observed ATE	Naive SE	Significant at *m* = 1 (naive)	Breaking ICC, *m* = 1.1	*m* = 1.25	*m* = 1.5	*m* = 2.0	*m* = 3.0
Prolonged length of stay (exploratory)	Unsuccessful-Discharge Proxy (Y2)	1,812	−0.064	0.012	Yes	Robust^c^	Robust^c^	Robust^c^	Robust^c^	Robust^c^
Prolonged length of stay (exploratory)	Log total treatment cost (Y3)	1,812	+1.099	0.028	Yes	Robust^c^	Robust^c^	Robust^c^	Robust^c^	Robust^c^
High insurance coverage (primary)	Unsuccessful-Discharge Proxy (Y2)	1,813	+0.022	0.014	No	n/a^d^	n/a^d^	n/a^d^	n/a^d^	n/a^d^
High insurance coverage (primary)	Log total treatment cost (Y3)	1,813	−0.507	0.035	Yes	Robust^c^	Robust^c^	Robust^c^	Robust^c^	Robust^c^
Continuous claims exposure (secondary)	Unsuccessful-Discharge Proxy (Y2)	1,722	−0.221	0.040	Yes	Robust^c^	Robust^c^	Robust^c^	Robust^c^	Robust^c^
Continuous claims exposure (secondary)	Log total treatment cost (Y3)	1,722	+2.529	0.088	Yes	Robust^c^	Robust^c^	Robust^c^	Robust^c^	Robust^c^

^a^DEFF ≈ 1 + (m − 1) × ICC (Kish, 1965); the naive variance was inflated by DEFF and significance at α = 0.05 was re-evaluated at each grid point. ^b^ The m = 1.0 case (no clustering, DEFF = 1 by construction) is omitted, as robustness is only meaningfully evaluated for m > 1. ^c^ “Robust” indicates that no ICC value on the tested grid (0.00–0.50) was sufficient to overturn statistical significance at that cluster size; 0.50 is the upper limit of the literature-informed sensitivity grid, not an estimated ICC. ^d^ Not applicable: this result was already non-significant at the naive (unclustered) standard error, so no degree of within-patient dependence can be said to have “broken” it.

All five statistically significant main average treatment effects — including both models built on the exploratory length-of-stay exposure — remained significant across the entire tested grid, up to an intraclass correlation of 0.50 combined with an assumed average cluster size of three episodes per patient, which is well above the plausible range implied by a chronic, progressively managed condition without a documented outbreak of repeat same-window admissions. The one non-significant primary result (high insurance coverage on the discharge-outcome proxy) remains non-significant irrespective of any clustering assumption, so this analysis neither strengthens nor weakens that finding. This robustness check addresses only the statistical-independence threat posed by unlinked repeat episodes; it does not, and is not intended to, address the separate concern that the length-of-stay exposure is realised within the same episode as its outcomes, which is why that exposure continues to be reported as exploratory rather than as a causal effect (Tables 21–22) even where it is shown here to be robust to within-patient dependence.

The same breaking-point analysis was repeated for all 60 subgroup-level average treatment effects reported alongside the six main models; results are summarised by robustness category in Table 25 and reported in full in S28 Table in S1 Appendix.

**Table 25:** Summary of within-patient-dependence robustness across all 66 fitted average-treatment-effect models (6 main models plus 60 subgroup models).

Robustness category	N models	% of 66
Already non-significant at the naive (unclustered) standard error — clustering assumption is not applicable	15	22.7%
Significant at the naive standard error and robust up to ICC = 0.50 at every tested cluster size (*m* = 1.1–3.0)	50	75.8%
Significant at the naive standard error but with a finite breaking ICC below the grid maximum at one or more cluster sizes	1	1.5%

Of the 51 models that were statistically significant under the naive, unclustered standard error, 50 (98.0%) retained significance across the full sensitivity grid; the single exception was a small, previously flagged subgroup (private-hospital episodes, *n* = 100, exploratory length-of-stay exposure on the discharge-outcome proxy), whose breaking ICC fell as low as 0.025–0.425 depending on the assumed cluster size, and which should accordingly be interpreted with the same caution already attached to its wide confidence interval (S25 Table in S1 Appendix). Because this sensitivity analysis sweeps a literature-informed range of plausible parameter values rather than estimating within-patient correlation directly from the data, it bounds — but cannot resolve — the risk posed by the absence of a patient identifier; should patient-level linkage become available, re-estimating the underlying models with a cluster-robust variance estimator at the patient level would be the definitive remedy.

## Discussion

This study set out to answer three questions: how insurance-level resource-allocation decisions causally shape recovery, discharge, cost and financial-burden outcomes among hospitalized Parkinson’s disease (PD) patients in Iran; whether these effects vary across clinically and administratively defined subgroups; and whether budget-neutral reallocation could simultaneously improve efficiency and equity. Findings for each question are considered in turn, in relation to prior claims-based PD literature.

For the first question, the three candidate resource-allocation levers occupied markedly different positions on the causal-inference spectrum, a distinction that governs how each result is discussed below. Prolonged length of stay was labelled the exploratory exposure throughout: it was associated with a 6.4-percentage-point reduction in the unsuccessful-discharge proxy (average treatment effect [ATE] −0.064, 95% CI −0.086 to −0.041) and a 200% increase in total treatment cost (ATE + 1.099 on the log scale, 95% CI 1.045 to 1.154), but because length of stay is itself the treatment and is mechanically entangled with the very outcomes used to construct the ancillary severity covariate, no validated pre-treatment clinical severity measure, such as Hoehn-Yahr stage or a UPDRS score, was available to support an ignorability assumption. These estimates are therefore reported, and should be read, as descriptive associations characterizing what happened to patients who experienced longer admissions, not as the causal effect of authorizing a longer stay; the corresponding E-values (point 2.50, limit 1.93, for discharge; point 4.26, limit 4.04, for cost) indicate that a comparatively modest unmeasured confounder could account for the weaker association, reinforcing the exploratory label rather than upgrading it. High insurance-coverage generosity was the primary exposure: it was associated with an approximately 40% reduction in total cost (ATE −0.507, 95% CI −0.572 to −0.438; E-value limit 2.19), while its association with the discharge-outcome proxy was small, statistically non-significant, and consistent with no effect (ATE + 0.022, 95% CI −0.005 to 0.049). Because coverage tier is a lever insurers can set in advance of admission, and its adjustment set was validated as pre-treatment, this is the only exposure-outcome pair for which both the point estimate and interval are compatible with a genuine causal effect; even so, adjustment for this observed, pre-treatment covariate set is not equivalent to identification of the true causal effect, and residual confounding from unobserved clinical severity remains a plausible, bounded, alternative explanation. Treatment intensity, the most weakly grounded exposure, showed a similarly large association with cost (+1,155%) that is explicitly non-causal, reflecting the mechanical link between billing-transaction volume and billed cost rather than a clinical or behavioural mechanism, and is retained only for descriptive completeness.

These findings sit alongside, and extend, previous claims-based PD literature. Liu et al.’s analysis of almost 20,000 Chinese inpatient episodes identified insurance type, alongside age and sex, as a significant correlate of length of stay and hospitalization cost, consistent with the leading role that administrative case-mix, prolonged stay and service group played among the predictors retained by the cost model here [76]. The coverage-cost finding agrees closely with Zhang et al.’s Guangzhou claims analysis, in which patients covered by the more generous employee-based insurance scheme incurred substantially lower out-of-pocket shares than those on the resident-based scheme, 24.3% versus 47.9%; the concentration of out-of-pocket burden among lower-socioeconomic-status episodes reported here (concentration index C = −0.056, 95% CI −0.077 to −0.035) is the equity-focused counterpart of that mechanism [77]. Agreement across two health systems with very different financing architectures strengthens the inference that coverage generosity, rather than any single country’s institutional idiosyncrasy, is the operative lever.

Where the present results diverge from prior work, the divergence is instructive rather than contradictory. Johnson et al.’s US commercial-claims analysis found that costs escalate sharply with PD progression, from roughly US$4,072 in newly diagnosed patients to US$37,410 among institutionalized patients [78], a severity gradient the reliability-tiered decomposition here does not reproduce at the level of socioeconomic inequality: where the decomposition was classified as reliable (residual share of C below 80%), the admission-time severity score contributed only −0.8% to +6.2% of the observed concentration index for recovery, cost and length of stay, far behind age (up to +42.4% for recovery) and hospital ownership. These are not competing claims about the same quantity: Johnson et al. describe how severity drives the level of cost, whereas the decomposition here describes what drives the socioeconomic distribution of cost. The contrast clarifies that in this Iranian sample, unequal financial protection is better explained by who a patient is administratively, in terms of age and hospital sector, than by how clinically severe their episode is coded as being.

The unsuccessful-discharge proxy requires particular caution when set against the wider literature. Crowther et al. reported 172 Medicare readmissions per 1,000 PD hospitalizations, while a UK teaching-hospital cohort of 712 people with Parkinson’s disease (PwP), accounting for 1,156 admissions, reported that 35% of admissions were followed by readmission within 30 days of discharge [79,80]; because the outcome used here is constructed from same-day discharge, non-recovery and adverse-discharge indicators within a single episode and is not linked to any subsequent admission, it cannot be equated with either readmission rate, and no such comparison is drawn anywhere in this manuscript. What can be compared is discriminative performance: the discharge-risk-proxy model’s out-of-fold AUC-ROC of 0.870 is broadly comparable to, though not directly equivalent to, the 0.774–0.832 range reported for a nationwide discharge-destination and mortality model built on 281,664 admissions [81], supporting the proxy’s predictive validity while leaving its relationship to true rehospitalization an open empirical question, one that the UK cohort’s findings on dementia, fracture and pneumonia as readmission risk factors underline the importance of resolving [80].

For the second research question, subgroup treatment effects for the exploratory and primary exposures were consistent in sign and approximate magnitude across age band (−8.6 to −4.3 percentage points for length of stay on the discharge proxy; −37.4% to −42.1% for coverage on cost), severity tier, hospital sector, rurality and socioeconomic rank, with the exception of an attenuated, imprecisely estimated effect in the small private-hospital stratum (n = 100). This homogeneity is informative when read against the Ontario rurality study, which found that rural PD patients used emergency care more, and family-physician and neurologist care less, than urban patients, interpreting this pattern as evidence of inequitable upstream access [82]. The absence of strong rural-urban treatment-effect heterogeneity here is not inconsistent with the Ontario findings; it instead suggests that rural disadvantage, to the extent it exists in this sample, is more likely concentrated in whether and how patients reach inpatient care than in how they respond to length-of-stay or coverage decisions once admitted, a distinction with direct implications for where equity-oriented policy should intervene.

Finally, for the third research question, the multinational finding that hospitalization odds rise sharply with PD severity in the United States and Europe, though far more modestly in Japan [83], supports in principle the severity-sensitive logic underlying the constrained welfare-optimization framework, while cautioning against transplanting those specific odds ratios into an Iranian, insurance-administered context. Under the primary, coverage-based scenario, the optimized allocation treated an estimated 35.8% of the cohort (95% CI 34.7–36.9%; welfare index W = 290.69) with an age-75-and-over targeting ratio close to 1.0 (0.968–1.075), indicating neither systematic over- nor under-targeting of older patients relative to their share of eligible episodes; the exploratory, length-of-stay-based scenario treated a smaller 27.3% of the cohort (W = 487.34) but is not used to support any allocation recommendation. These optimization results are presented as model-based counterfactual reallocation scenarios rather than as empirically demonstrated policy effects, since provider, insurer and patient behaviour could plausibly change under an implemented reallocation in ways the estimated treatment effects cannot capture. Taken together, the three questions are answered with deliberately uneven confidence: the coverage-cost relationship is the study’s most defensible causal claim, subgroup heterogeneity is limited but real in one small stratum, and the reallocation scenarios remain a normative exercise built on that single causal claim rather than a tested intervention.

### Theoretical and policy implications

Theoretically, this study contributes less at the level of any single technique than at the level of their integration. Double machine learning, causal forests, concentration-index decomposition and constrained integer-linear-programming optimization have each previously been validated separately in neurodegenerative or chronic-disease populations; assembling them into one policy-oriented pipeline, applied for the first time to Iranian administrative PD insurance data, offers a replicable template for causal-equity-optimization analysis in low- and middle-income health systems that lack the patient-level registries typical of high-income settings. A second theoretical contribution is methodological rather than substantive: the explicit three-way stratification of exposures into associational, causal-under-a-stated-assumption, and mechanically non-causal categories, together with the admission-time-only construction of the severity covariate and its complete-removal robustness check, offers other researchers working with single-timepoint administrative claims a concrete template for avoiding post-treatment adjustment bias when the treatment of interest is itself realized during the encounter being studied.

The policy implications that follow are deliberately narrower than the full set of findings, in proportion to the strength of the underlying evidence. Because coverage generosity (T2) is the only exposure supported by a causal estimate under a stated, testable identification assumption, and because it was associated with a large reduction in total cost that is consistent, in direction, with the socioeconomic concentration of out-of-pocket burden reported here and with the coverage-related financial-protection gradient reported by Zhang et al. in Guangzhou, harmonizing benefit-package generosity across Iran’s fragmented insurance funds is the recommendation best supported by this evidence, rather than a general call to expand health-system spending [77]. Because age and hospital ownership, not disease severity, were the dominant observed correlates of the socioeconomic gradient in cost, length of stay and recovery, policy attention directed at administrative and structural sources of inequity, such as tariff differences between private and public providers and age-related gaps in benefit design, is likely to be more productive than efforts framed solely around clinical severity. The budget-neutral reallocation scenarios, in which the coverage-based optimization exhausted the insurer-cost budget while using only a minority of the bed-day budget, illustrate that efficiency and equity objectives are not necessarily in tension under this framework, but, consistent with their explicitly normative construction, they should inform phased, monitored pilots rather than direct implementation, since the estimated treatment effects that underlie them could shift once providers, insurers and patients respond to a changed allocation rule.

### Limitations and future research

Several limitations temper these conclusions and should guide how the findings are used. First, the analytic dataset carries no patient identifier, so multiple admissions from the same patient may have been treated as independent episodes; a dedicated design-effect sensitivity analysis showed that the great majority of significant associations remained robust across a literature-informed range of within-patient correlation, but this sweep bounds rather than resolves the risk, and patient-level linkage, if it becomes available, would allow this limitation to be addressed directly with cluster-robust inference. Second, the unsuccessful-discharge proxy (Y2) is constructed from same-day discharge, non-recovery and adverse-discharge indicators within a single episode and is not a linked, longitudinally observed thirty-day readmission; all effect estimates and equity indices involving this outcome are reported, and should be read, as evidence about discharge risk rather than about rehospitalization, and obtaining or linking subsequent-admission data is a priority for future work. Third, although the causal models were restricted to a validated set of pre-treatment covariates and the E-values attached to the coverage and length-of-stay estimates indicate that a fairly strong unmeasured confounder would be required to fully explain them away, the underlying claims data lack validated Parkinson’s disease stage, UPDRS or Hoehn-Yahr scores, comorbidity burden, medication regimens, caregiver support and finer socioeconomic detail; adjustment for observed confounding under a pre-specified, validated directed acyclic graph is not equivalent to identification of the true causal effect, and residual confounding from these unobserved clinical variables remains plausible. Fourth, the welfare-optimization scenarios are explicitly normative rather than causal: they translate estimated conditional treatment effects into counterfactual allocations under a fixed budget and welfare specification, but do not, and cannot, establish what would occur after implementation, since provider, insurer and patient behaviour could change in response to a new allocation rule. Fifth, the study is confined to a roughly two-year observation window within a single, though nationally structured, insurance system across six provinces; longer-term Taiwanese evidence suggests that a short observation window may understate the cumulative economic burden of Parkinson’s disease [84], and the private-hospital subgroup in particular was small and imprecisely estimated, warranting caution against over-interpreting its attenuated effect sizes.

These limitations point directly to a future research agenda. The most consequential next step is establishing patient-level linkage within Iranian claims data, which would simultaneously resolve the within-patient dependence problem and permit construction of a genuine thirty-day, or longer, readmission outcome to replace the discharge-risk proxy used here. A second priority is incorporating validated clinical severity measures, such as Hoehn-Yahr stage or UPDRS scores, alongside comorbidity and medication data, ideally through linkage with outpatient and pharmacy claims, to strengthen the pre-treatment confounder set and narrow the residual-confounding bounds indicated by the E-value analysis. Third, because the optimization results are scenario-based rather than validated, phased or pilot implementation of coverage-harmonization policies, with prospective monitoring of recovery, cost and discharge outcomes, is needed before the reallocations proposed here are treated as demonstrated policy effects. Finally, extending the observation window and linking inpatient episodes with long-term and outpatient care, as has been done in Taiwan and Luxembourg would allow the substantial inpatient-cost burden identified here to be situated within the fuller lifetime economic burden of Parkinson’s disease in Iran.

## Conclusion

This study provides the first causal-inference and equity evidence for hospitalized Parkinson’s disease care within Iran’s fragmented insurance system, distinguishing carefully among an associational length-of-stay pattern, a causal coverage-cost effect estimated under a stated pre-treatment identification strategy, and a mechanical, non-causal treatment-intensity association. Its central substantive finding, that higher insurance-coverage generosity causally reduces total cost and that socioeconomic inequity in cost, out-of-pocket burden and length of stay is driven primarily by administrative factors such as age and hospital ownership rather than by clinical severity, offers a more actionable target for reform than severity alone would suggest. The novelty of the work lies less in any individual causal or equity method than in combining them, together with constrained optimization, into a single replicable pipeline for LMIC insurance systems facing comparable pressures. Because the reallocation scenarios derived from this pipeline are explicitly normative and rest on a discharge-risk proxy rather than observed readmission, their value at this stage is as a decision-support tool for phased, monitored piloting of benefit-package harmonization, not as a validated policy prescription. Realizing the framework’s full potential will depend on the specific extensions identified above, patient-level linkage, validated clinical severity data, and genuine longitudinal readmission outcomes, which together would allow future work to move from bounded, assumption-dependent causal estimates toward more definitive evidence on how resource allocation shapes recovery, equity and cost for Parkinson’s disease patients in Iran and comparable middle-income health systems.

## Supporting information

S1 Appendix Supplementary information. Additional analyses, tables, figures, robustness assessments, subgroup analyses, and calibration evaluations supporting the main manuscript.
(DOCX)

## References

[1] Li M, Ye X, Huang Z, Ye L, Chen C. Global burden of Parkinson’s disease from 1990 to 2021: a population-based study. BMJ Open. 2025;15(4):e095610. doi: 10.1136/bmjopen-2024-095610 40288800 PMC12035419

[2] Luo Y, Qiao L, Li M, Wen X, Zhang W, Li X. Global, regional, national epidemiology and trends of Parkinson’s disease from 1990 to 2021: findings from the Global Burden of Disease Study 2021. Front Aging Neurosci. 2025;16:1498756. doi: 10.3389/fnagi.2024.1498756 39868382 PMC11757241

[3] Peng S, Liu P, Wang X. Global, regional and national burden of Parkinson’s disease in people over 55 years of age: a systematic analysis of the global burden of disease study, 1991–2021. BMC Neurol. 2025;25:178. doi: 10.1186/s12883-025-04191-8 PMC12016353 40269818

[4] Dorsey ER, Sherer T, Okun MS, Bloem BR. The Emerging Evidence of the Parkinson Pandemic. J Parkinsons Dis. 2018;8(s1):S3–8. doi: 10.3233/JPD-181474 30584159 PMC6311367

[5] The Lancet. What next in Parkinson’s disease?. Lancet. 2024;403(10423):219. doi: 10.1016/S0140-6736(24)00094-1 38245237

[6] Chaudhuri KR, Azulay J-P, Odin P, Lindvall S, Domingos J, Alobaidi A, et al. Economic Burden of Parkinson’s Disease: A Multinational, Real-World, Cost-of-Illness Study. Drugs Real World Outcomes. 2024;11(1):1–11. doi: 10.1007/s40801-023-00410-1 38193999 PMC10928026

[7] Song Y, E JY, Guo T, Sasane R, Arcona S, Keshava N, et al. Treatment patterns and healthcare resource use in Medicare beneficiaries with Parkinson’s disease. Clinicoecon Outcomes Res. 2023;15:631–43. doi: 10.2147/CEOR.S422023 37551376 PMC10404422

[8] Kruse C, Lipinski A, Verheyen M, Balzer-Geldsetzer M, Wittenberg M, Lorenzl S, et al. Care of Late-Stage Parkinsonism: Resource Utilization of the Disease in Five European Countries. Mov Disord. 2024;39(3):571–84. doi: 10.1002/mds.29718 38425158

[9] Crighton EJ, Ouédraogo AM, Sawada M, Mestre TA. Patterns and determinants of health care utilization among people with Parkinson’s disease: A population-based analysis in Ontario, Canada. PLoS One. 2024;19(6):e0305062. doi: 10.1371/journal.pone.0305062 38905210 PMC11192415

[10] Salari M, Etemadifar M, Vakilian A, Chitsaz A, Shafiei K, Nabavi SM, et al. A report of the Iranian Parkinson’s disease registry. NPJ Parkinsons Dis. 2025;11(1):251. doi: 10.1038/s41531-025-01108-7 40841537 PMC12370882

[11] Sarmadi M, Rezaei M, Poursadeghiyan M, Soltaninejad M, Moradi S, Ahangarzadeh M, et al. Burden of Parkinson’s disease in Iran: disparities, trends, and the impact of social development indicators. Neurol Sci. 2025;46(8):3651–62. doi: 10.1007/s10072-025-08206-7 40317438

[12] Rezaei S, Karimi M, Soltani S, Barfar E, Gharehghani MAM, Badakhshan A, et al. Household financial burden associated with out-of-pocket payments for healthcare in Iran: insights from a cross-sectional survey. BMC Health Serv Res. 2024;24(1):1062. doi: 10.1186/s12913-024-11477-z 39272081 PMC11396140

[13] Doshmangir L, Bazyar M, Rashidian A, Gordeev VS. Iran health insurance system in transition: equity concerns and steps to achieve universal health coverage. Int J Equity Health. 2021;20(1):37. doi: 10.1186/s12939-020-01372-4 33446202 PMC7807408

[14] Malmir S, Navipour H, Negarandeh R. Exploring challenges among Iranian family caregivers of seniors with multiple chronic conditions: a qualitative research study. BMC Geriatr. 2022;22(1):270. doi: 10.1186/s12877-022-02881-3 35365077 PMC8973877

[15] Piroozi B, Rashidian A, Moradi G, Takian A, Ghasri H, Ghadimi T. Out-of-Pocket and Informal Payment Before and After the Health Transformation Plan in Iran: Evidence from Hospitals Located in Kurdistan, Iran. Int J Health Policy Manag. 2017;6(10):573–86. doi: 10.15171/ijhpm.2017.16 28949473 PMC5627785

[16] Sasidharan A, Bagepally BS, Kumar SS. Cost Effectiveness of Deep Brain Stimulation for Parkinson’s Disease: A Systematic Review. Appl Health Econ Health Policy. 2024;22(2):181–92. doi: 10.1007/s40258-023-00848-y 38015368

[17] van Munster M, Stümpel J, Thieken F, Pedrosa DJ, Antonini A, Côté D, et al. Moving towards Integrated and Personalized Care in Parkinson’s Disease: A Framework Proposal for Training Parkinson Nurses. J Pers Med. 2021;11(7):623. doi: 10.3390/jpm11070623 34209024 PMC8304750

[18] Di Luca DG, Shalash A. Addressing Variability and Bridging the Gap in Parkinson’s Disease Care: A Call to Action. Mov Disord Clin Pract. 2025;12(12):2055–7. doi: 10.1002/mdc3.70255 40696860 PMC12715327

[19] Prema SS, Ganapathy D, Shanmugamprema D. AI-driven epidemiology: The next frontier in precision public health. Wiley Interdisciplinary Reviews: Data Mining and Knowledge Discovery. 2026;16(2):e70077. doi: 10.1002/widm.70077

[20] Jiang X, Lv G, Li M, Lu K. Causal effect of anti-dementia drugs on patients’ economic burden using double/debiased machine learning approach. Innov Aging. 2023;7(Suppl 1):1000. doi: 10.1093/geroni/igad104.3213

[21] Pierce A. Causal inference and machine learning methods in Parkinson’s Disease data analysis. Orange, CA: Chapman University. 2023.

[22] Xue Z, Lu H, Zhang T, Guo X, Gao L. Remote Parkinson’s disease severity prediction based on causal game feature selection. Expert Systems with Applications. 2024;241:122690. doi: 10.1016/j.eswa.2023.122690

[23] Chernozhukov V, Chetverikov D, Demirer M, Duflo E, Hansen C, Newey W, et al. Double/debiased machine learning for treatment and structural parameters. The Econometrics Journal. 2018;21(1):C1–68. doi: 10.1111/ectj.12097

[24] Athey S, Imbens G. Recursive partitioning for heterogeneous causal effects. Proc Natl Acad Sci U S A. 2016;113(27):7353–60. doi: 10.1073/pnas.1510489113 27382149 PMC4941430

[25] Wager S, Athey S. Estimation and Inference of Heterogeneous Treatment Effects using Random Forests. Journal of the American Statistical Association. 2018;113(523):1228–42. doi: 10.1080/01621459.2017.1319839

[26] Almasi M, Anahideh H. Bi-Level Contextual Bandits for Individualized Resource Allocation Under Delayed Feedback. AAAI. 2026;40(45):38184–93. doi: 10.1609/aaai.v40i45.41157

[27] Adekunle H. Fair Reinforcement Learning for Equitable Health Resource Allocation. JDACM. 2025;1(02):91–7. doi: 10.64235/27s4qp61

[28] Wilder B, Welle P. Learning treatment effects while treating those in need. In: Proceedings of the 26th ACM Conference on Economics and Computation, 2025. 448–73. 10.1145/3736252.3742570

[29] Hani M, Mahmoudi S, Benjelloun M. AHN-BudgetNet: Cost-Aware Multimodal Feature-Acquisition Architecture for Parkinson’s Disease Monitoring. Electronics. 2025;14(17):3502. doi: 10.3390/electronics14173502

[30] Wu F, Demeulemeester E, Matuschke J. Robust Deterministic Policies for Markov Decision Processes under Budgeted Uncertainty. 2024. https://arxiv.org/abs/2412.12879

[31] Diaz-Rincon R, Liang M, Ramirez-Zamora A, Shickel B. Uncertainty-aware prediction of Parkinson’s disease medication needs: A two-stage conformal prediction approach. 2025. https://arxiv.org/abs/2508.10284

[32] Harris S, Katz Z, Smith M, Mochan A, Ferreira-Correia A. Disparities in access to treatment in relation to quality of life in people diagnosed with Parkinson’s disease in the South African healthcare setting. S Afr Med J. 2025;115(3):e2297. doi: 10.7196/SAMJ.2025.v115i3.2297 41378525

[33] Battaglini Chehin A, Rahal Abrahão L, Mira Fuga MA. Demographics, treatment patterns, and healthcare resource utilization in Parkinson’s disease: a real-world data study using a claims database. Arquivos de Neuro-Psiquiatria. 2026;84(1). doi: 10.1055/s-0045-1814375 PMC12832158 41581498

[34] Kamo H, Mehta TR, Remz M, Burke RM, Brooks A, Smiley A, et al. Machine learning prediction of discharge destination in patients with Parkinson’s disease; a nationwide cohort study. NPJ Parkinsons Dis. 2026;12(1):120. doi: 10.1038/s41531-026-01309-8 41896562 PMC13194952

[35] Liu Z, Yang Y, Mo G, Tan C, Zhang W, Jia W. The impact of long-term care insurance on the subjective well-being of middle-aged and older individuals in rural China an empirical analysis based on CHARLS data. Front Public Health. 2025;13:1662574. doi: 10.3389/fpubh.2025.1662574 41132180 PMC12540081

[36] Fang J, Meng D, Fang B. Social determinants of health and their association with Parkinson’s disease prevalence in US adults: insights from NHANES 2001-2020. Behav Neurol. 2026;2026(1):e3745561. doi: 10.1155/bn/3745561 42338381 PMC13291721

[37] Denham I, Maurin R, Deltetto I, Mikolaizak AS, Waern JB, Taylor C. Levodopa-based device-aided therapies for the treatment of advanced Parkinson’s disease: a social return on investment analysis. Front Public Health. 2024;12:1351808. doi: 10.3389/fpubh.2024.1351808 38979043 PMC11228815

[38] Aryankhesal A, Etemadi M, Mohseni M, Azami-Aghdash S, Nakhaei M. Catastrophic Health Expenditure in Iran: A Review Article. Iran J Public Health. 2018;47(2):166–77. 29445626 PMC5810379

[39] Erreygers G. Correcting the concentration index. J Health Econ. 2009;28(2):504–15. doi: 10.1016/j.jhealeco.2008.02.003 18367273

[40] Wagstaff A, van Doorslaer E. Catastrophe and impoverishment in paying for health care: with applications to Vietnam 1993-1998. Health Econ. 2003;12(11):921–34. doi: 10.1002/hec.776 14601155

[41] O’Donnell O, van Doorslaer E, Wagstaff A, Lindelow M. Analyzing health equity using household survey data: a guide to techniques and their implementation. Washington DC: World Bank Publications. 2008. doi: 10.1596/978-0-8213-6933-3

[42] George BP, Barbosa WA, Sethi A, Richard IH. Complications and outcomes of hospitalizations for patients with and without Parkinson disease. Front Aging Neurosci. 2023;15:1276731. doi: 10.3389/fnagi.2023.1276731 38161593 PMC10757345

[43] Temlett JA, Thompson PD. Reasons for admission to hospital for Parkinson’s disease. Intern Med J. 2006;36(8):524–6. doi: 10.1111/j.1445-5994.2006.01123.x 16866658

[44] Iezzoni LI. Risk adjustment for measuring health care outcomes. 4th ed. Chicago: Health Administration Press. 2012.

[45] Elixhauser A, Steiner C, Harris DR, Coffey RM. Comorbidity measures for use with administrative data. Med Care. 1998;36(1):8–27. doi: 10.1097/00005650-199801000-00004 9431328

[46] van Walraven C, Austin PC, Jennings A, Quan H, Forster AJ. A modification of the Elixhauser comorbidity measures into a point system for hospital death using administrative data. Med Care. 2009;47(6):626–33. doi: 10.1097/MLR.0b013e31819432e5 19433995

[47] Royston P, Altman DG, Sauerbrei W. Dichotomizing continuous predictors in multiple regression: a bad idea. Stat Med. 2006;25(1):127–41. doi: 10.1002/sim.2331 16217841

[48] Busse R, Geissler A, Aaviksoo A, Cots F, Häkkinen U, Kobel C, et al. BMJ. 2013;346:f3197. doi: 10.1136/bmj.f3197 23747967

[49] Goodney PP, Stukel TA, Lucas FL, Finlayson EVA, Birkmeyer JD. Hospital volume, length of stay, and readmission rates in high-risk surgery. Ann Surg. 2003;238(2):161–7. doi: 10.1097/01.SLA.0000081094.66659.c3 12894006 PMC1422689

[50] Manning WG, Newhouse JP, Duan N, Keeler EB, Leibowitz A, Marquis MS. Health insurance and the demand for medical care: evidence from a randomized experiment. Am Econ Rev. 1987;77(3):251–77. 10284091

[51] Kansagara D, Englander H, Salanitro A, Kagen D, Theobald C, Freeman M, et al. Risk prediction models for hospital readmission: a systematic review. JAMA. 2011;306(15):1688–98. doi: 10.1001/jama.2011.1515 22009101 PMC3603349

[52] Duan N. Smearing Estimate: A Nonparametric Retransformation Method. Journal of the American Statistical Association. 1983;78(383):605–10. doi: 10.1080/01621459.1983.10478017

[53] Manning WG, Mullahy J. Estimating log models: to transform or not to transform?. J Health Econ. 2001;20(4):461–94. doi: 10.1016/s0167-6296(01)00086-8 11469231

[54] Macleod AD, Taylor KSM, Counsell CE. Mortality in Parkinson’s disease: a systematic review and meta-analysis. Mov Disord. 2014;29(13):1615–22. doi: 10.1002/mds.25898 24821648

[55] Picillo M, Amboni M, Erro R, Longo K, Vitale C, Moccia M, et al. Gender differences in non-motor symptoms in early, drug naïve Parkinson’s disease. J Neurol. 2013;260(11):2849–55. doi: 10.1007/s00415-013-7085-x 23989344

[56] Drummond MF, Sculpher MJ, Claxton K, Stoddart GL, Torrance GW. Methods for the economic evaluation of health care programmes. 4th ed. Oxford: Oxford University Press. 2015.

[57] Russell LB, Gold MR, Siegel JE, Daniels N, Weinstein MC. The role of cost-effectiveness analysis in health and medicine. JAMA. 1996;276(14):1172–7. 8827972

[58] Culyer AJ, Wagstaff A. Equity and equality in health and health care. J Health Econ. 1993;12(4):431–57. doi: 10.1016/0167-6296(93)90004-x 10131755

[59] Swanson SA, Hernán MA. Think globally, act globally: An epidemiologist’s perspective on instrumental variable estimation. Stat Sci. 2014;29(3):371–4. doi: 10.1214/14-sts491 25580054 PMC4285626

[60] Belsley DA, Kuh E, Welsch RE. Regression diagnostics: identifying influential data and sources of collinearity. New York: Wiley. 1980.

[61] Rothman KJ. No adjustments are needed for multiple comparisons. Epidemiology. 1990;1(1):43–6. doi: 10.1097/00001648-199001000-00010 2081237

[62] Bergstra J, Bardenet R, Bengio Y, Kégl B. Algorithms for Hyper-Parameter Optimization. In: Proceedings of the 24th International Conference on Neural Information Processing Systems, Granada, 2011. 2546–54.

[63] Bergstra J, Bengio Y. Random Search for Hyper-Parameter Optimization. Journal of Machine Learning Research. 2012;13:281–305.

[64] Niculescu-Mizil A, Caruana R. Predicting good probabilities with supervised learning. In: Proceedings of the 22nd international conference on Machine learning - ICML ’05, 2005. 625–32. 10.1145/1102351.1102430

[65] Brier GW. Verification of forecasts expressed in terms of probability. Mon Wea Rev. 1950;78(1):1–3. doi: 10.1175/1520-0493(1950)078<0001:vofeit>2.0.co;2

[66] Lundberg SM, Lee S-I. A Unified Approach to Interpreting Model Predictions. In: Proceedings of the 31st International Conference on Neural Information Processing Systems, Long Beach, 2017. 4766–77.

[67] Robins JM, Rotnitzky A, Zhao LP. Estimation of Regression Coefficients When Some Regressors are not Always Observed. Journal of the American Statistical Association. 1994;89(427):846–66. doi: 10.1080/01621459.1994.10476818

[68] Newey WK, McFadden D. Chapter 36 Large sample estimation and hypothesis testing. Handbook of Econometrics. Elsevier. 1994. p. 2111–245. doi: 10.1016/s1573-4412(05)80005-4

[69] Crump RK, Hotz VJ, Imbens GW, Mitnik OA. Dealing with limited overlap in estimation of average treatment effects. Biometrika. 2009;96(1):187–99. doi: 10.1093/biomet/asn055

[70] Künzel SR, Sekhon JS, Bickel PJ, Yu B. Metalearners for estimating heterogeneous treatment effects using machine learning. Proc Natl Acad Sci U S A. 2019;116(10):4156–65. doi: 10.1073/pnas.1804597116 30770453 PMC6410831

[71] van Doorslaer E, Wagstaff A, van der Burg H, Christiansen T, De Graeve D, Duchesne I, et al. Equity in the delivery of health care in Europe and the US. J Health Econ. 2000;19(5):553–83. doi: 10.1016/s0167-6296(00)00050-3 11184794

[72] Williams S, Prior SJ. Health Service Improvement for People with Parkinson’s Disease: A Scoping Review. JAL. 2024;4(3):234–51. doi: 10.3390/jal4030017

[73] Forrest J, Lougee-Heimer R. CBC User Guide. Emerging Theory, Methods, and Applications. INFORMS. 2005. p. 257–77. doi: 10.1287/educ.1053.0020

[74] DiCiccio TJ, Efron B. Bootstrap confidence intervals. Statist Sci. 1996;11(3):189–228. doi: 10.1214/ss/1032280214

[75] Rosenbaum PR. Observational Studies. 2nd ed. New York: Springer-Verlag. 2002.

[76] Liu W, Nie P, Zhang J, Zhou D, Zhang J, Chen J. Clinical and economic insights into Parkinson’s disease hospitalization: a comprehensive study of 19,719 inpatient cases in Hubei province, China. Neuroepidemiology. 2024;58(4):237–46. doi: 10.1159/000536525 38290491 PMC11302739

[77] Zhang H, Zhou W, Zhang D. Direct Medical Costs of Parkinson’s Disease in Southern China: A Cross-Sectional Study Based on Health Insurance Claims Data in Guangzhou City. Int J Environ Res Public Health. 2022;19(6):3238. doi: 10.3390/ijerph19063238 35328925 PMC8953775

[78] Johnson SJ, Kaltenboeck A, Diener M, Birnbaum HG, Grubb E, Castelli-Haley J, et al. Costs of Parkinson’s disease in a privately insured population. Pharmacoeconomics. 2013;31(9):799–806. doi: 10.1007/s40273-013-0075-0 23907717 PMC3757266

[79] Mantri S, Fullard ME, Beck J, Willis AW. State-level prevalence, health service use, and spending vary widely among Medicare beneficiaries with Parkinson disease. NPJ Parkinsons Dis. 2019;5:1. doi: 10.1038/s41531-019-0074-8 30701188 PMC6345811

[80] Crowther G, Cosgrove J, Turpin P, Htun E, Henderson M. Predictors of outcomes in people with Parkinson’s disease admitted to a large UK teaching hospital. Parkinsonism Relat Disord. 2025;135:107808. doi: 10.1016/j.parkreldis.2025.107808 40250259

[81] Kamo H, Mehta TR, Remz M, Burke RM, Brooks A, Smiley A, et al. Machine learning prediction of discharge destination in patients with Parkinson’s disease; a nationwide cohort study. NPJ Parkinsons Dis. 2026;12(1):120. doi: 10.1038/s41531-026-01309-8 41896562 PMC13194952

[82] Maclagan LC, Marras C, Sewell IJ, Wu CF, Butt DA, Tu K, et al. Trends in health service use among persons with Parkinson’s disease by rurality: A population-based repeated cross-sectional study. PLoS One. 2023;18(5):e0285585. doi: 10.1371/journal.pone.0285585 37205650 PMC10198504

[83] Chaudhuri KR, Azulay J-P, Odin P, Lindvall S, Domingos J, Alobaidi A, et al. Economic Burden of Parkinson’s Disease: A Multinational, Real-World, Cost-of-Illness Study. Drugs Real World Outcomes. 2024;11(1):1–11. doi: 10.1007/s40801-023-00410-1 38193999 PMC10928026

[84] Chen KC, Ku LE, Hu YH, Sun Y, Elbaz A, Lee PC. Healthcare utilization and costs for patients with Parkinson’s disease in Taiwan. BMC Neurol. 2025;25(1):3. doi: 10.1186/s12883-024-03988-3 39754138 PMC11697947

