# Supplementary material for: Resource allocation under uncertainty and equity constraints in Parkinson’s disease care: An econometrically grounded machine learning framework
Source: PLoS One. 2026 Nov 3;21(11):e0360944. doi: 10.1371/journal.pone.0360944 (PMC13730354; doi:10.1371/journal.pone.0360944)
Supplement: Supplementary file 1 [file pone.0360944.s001.docx]

**Supplementary**

This appendix provides the complete methodological details underlying the six-stage analytical framework summarized in the main manuscript. It contains the full specifications for the Gradient Boosting Machine models used for outcome prediction (Stage I), the Double Machine Learning procedure for causal effect estimation (Stage II), the Causal Forest approach for heterogeneous treatment effect identification (Stage III), the equity measurement and decomposition methods (Stage IV), the constrained welfare optimization framework (Stage V), and the multi-layer uncertainty quantification strategy (Stage VI). The supplementary materials include comprehensive diagnostic tables, hyperparameter selections, cross-validation performance metrics, subgroup analyses, sensitivity checks for unmeasured confounding, within-patient dependence assessments, threshold robustness evaluations, and detailed results from the causal directed acyclic graph specification, equity decompositions, and welfare optimization sensitivity grids. All section numbers (S1–S7), equation numbers (Eq S1–S7), table references, and figure citations correspond directly to the pointers given in the main text, providing readers with transparent access to the analytical decisions, model diagnostics, and robustness assessments that support the study's conclusions.

**Appendix. Supplementary Methods (S1–S7)**

*This appendix provides the full model specifications, hyperparameters, diagnostic procedures, and supporting equations for the six analytical stages summarised in the Methods section of the main manuscript. Section numbers (S1–S7) and equation numbers (Eq S1–S7) correspond to the pointers given in the main text. Reference numbers correspond to the manuscript's main reference list.*

**S1 Appendix. Stage I: Outcome Prediction with Gradient Boosting Machines**

The primary objective of Stage I is twofold: to produce well-calibrated outcome predictions for descriptive and risk-stratification purposes, and to generate out-of-fold (OOF) nuisance estimates ṗ(X) = Ê[Y|X] that serve as inputs to the DML cross-fitting procedure in Stage II. This dual role imposes stringent requirements on the prediction models: they must (a) capture non-linear, high-dimensional covariate relationships without restrictive functional-form assumptions, and (b) be fitted on held-out data to ensure that nuisance predictions are not contaminated by in-sample overfitting, which would otherwise bias the DML ATE estimator (1).

Gradient Boosting Machines (GBM) implemented via the XGBoost algorithm were selected to satisfy these requirements for five reasons linked directly to the study's data characteristics (2). First, GBM makes no parametric assumptions about the functional form linking covariates to outcomes, which is critical in this administrative dataset where the joint distribution of severity, insurance type, rurality, and care utilisation is unlikely to conform to the additive linearity required by logistic or linear regression. Second, XGBoost natively accommodates the distributional heterogeneity across the five outcomes without transforming or reparameterising the outcome scale: the binary:logistic objective applies the canonical link for Bernoulli-distributed Y₁ and Y₂; reg:squarederror is appropriate for the approximately normal residuals of log-transformed cost Y₃; reg:tweedie with variance power 1.5 is the theoretically correct loss for Y₄, which follows a compound Poisson-Gamma distribution (zero-inflated, strictly positive, right-skewed) characteristic of out-of-pocket ratio data in mixed insurance systems (3); and count:poisson applies the canonical Poisson log-link for the non-negative integer LOS outcome Y₅, outperforming negative-binomial specifications when overdispersion is moderate, as verified via the deviance ratio diagnostic. Third, tree-ensemble splits are performed on individual variables, making GBM inherently robust to the multicollinearity documented in the variance-inflation-factor analysis, where total_requested and total_paid exceeded VIF = 300; unlike ridge or LASSO regression, GBM does not require explicit regularisation of collinear predictors because splits on redundant variables are penalised implicitly by information-gain thresholding. Fourth, GBM automatically discovers higher-order interactions among covariates (e.g., severity × insurance type × rurality) that would require explicit pre-specification in generalised linear models. Fifth, GBM's leaf-node regularisation (L₁/L₂ penalties on leaf weights, minimum child weight) provides built-in protection against overfitting on the study's moderate sample size (n = 1,813 episodes), reducing variance in OOF predictions subsequently used as nuisance estimates.

Hyperparameters (number of estimators, learning rate, maximum tree depth, subsample fraction, column subsample fraction, L₁ and L₂ leaf penalties) were optimised jointly using Bayesian search with the Tree-structured Parzen Estimator (4). TPE was preferred over grid search and random search because it uses the history of evaluated configurations to build probabilistic models of the promising hyperparameter regions, achieving equivalent or superior performance in substantially fewer trials than random search (5). Fifty trials were specified per outcome, determined by a pilot saturation analysis confirming that validation AUC/R² converged within the first 40 trials, providing a 25% margin. Search was conducted within the outer cross-validation loop to prevent hyperparameter leakage.

Model evaluation used stratified five-fold cross-validation with OOF prediction aggregation. Five folds were selected as they yield an 80/20 train-test split per fold, providing sufficient training data for the high-capacity GBM while maintaining a held-out evaluation set large enough (n ≈ 363 per fold) for reliable AUC and calibration estimation; ten-fold CV was considered but would have produced held-out sets of only ≈181 observations, inflating variance in the Hosmer–Lemeshow statistic. Binary outcomes were evaluated by AUC-ROC (overall discrimination), AUC-PR (performance under class imbalance), Brier score (proper scoring rule assessing probabilistic calibration) (6), and the Hosmer–Lemeshow statistic (g = 10 deciles) as a formal goodness-of-fit test.

Probability calibration was applied post hoc to correct for the known tendency of tree-ensemble classifiers to produce poorly calibrated probability outputs when trained with cross-entropy loss (7). Isotonic regression calibration was applied to Y₂ because the composite binary target produces raw score distributions that are non-monotonic and non-sigmoidal; isotonic regression makes no shape assumptions about the calibration mapping and outperforms Platt scaling under such conditions (7). Platt scaling (logistic calibration) was applied to Y₁ because the recovery outcome produces approximately sigmoid raw scores consistent with Platt's assumptions, and its lower variance relative to isotonic regression is advantageous for the smaller effective calibration sample. Feature importance was extracted as mean split gain across folds; Shapley Additive Explanations (SHAP) were additionally computed on the held-out fold to quantify marginal directional contributions (8), preferred over impurity-based importance because the latter is biased toward high-cardinality continuous variables and is unstable under collinearity (9).

**S2 Appendix. Stage II: Causal Effect Estimation via Double Machine Learning**

Double Machine Learning in the Partially Linear Regression (PLR) formulation was selected as the causal estimator for three reasons directly connected to the study's design and objective (10, 11) . First, DML is specifically designed for the setting where treatment assignment is confounded by a large number of observed covariates and the researcher wishes to obtain a low-dimensional causal parameter (the ATE) without imposing a parametric model on the high-dimensional nuisance functions E[Y|X] and E[T|X]; this is precisely the structure of Iranian hospital insurance data where dozens of administrative, clinical, and socioeconomic covariates jointly influence both treatment decisions and outcomes. Second, unlike propensity-score matching or inverse probability weighting — which require a correctly specified propensity model — DML achieves √n-consistent, asymptotically normal inference on θ even when the nuisance models converge at slower-than-parametric rates (e.g., GBM with rate n⁻¹ᐟ⁴), because the cross-fitting procedure eliminates the Neyman orthogonality bias that would otherwise contaminate the second-stage estimate (10). Third, difference-in-differences and instrumental-variable designs were considered but found unsuitable: difference-in-differences requires repeated pre-post observations at the patient level, which are unavailable in single-admission episode data; instrumental variables require a valid instrument affecting treatment but not outcomes except through treatment, and no credible instrument was available in the administrative dataset. Regression discontinuity was similarly infeasible due to the absence of a sharp eligibility threshold in the insurance assignment rules.

The cross-fitting procedure used K = 5 folds, chosen to balance bias–variance in the nuisance estimates: fewer folds reduce the training sample for nuisance fitting (increasing approximation error), while more folds reduce the held-out evaluation sample (increasing second-stage estimation variance); K = 5 is the canonical choice in the DML literature for sample sizes in the range n ∈ [500, 5,000] (10). In each fold, GBM nuisance models were fitted on n = 1,450 training observations and applied to the n = 363 held-out observations to produce residuals

$$\boldsymbol{\varepsilon̂ᵢ = Yᵢ - Ê[Yᵢ|Xᵢ] and \nûᵢ = Tᵢ - Ê[Tᵢ|Xᵢ]}.$$

Standard errors were computed via the influence-function sandwich estimator (12):

$$\boldsymbol{SE(\thetâ) = \surd[ (1/n) E[\psiᵢ^{2}] / (E[\nûᵢ^{2}])^{2} ], where \psiᵢ = (\varepsilonᵢ - \thetâ \nuᵢ) \nuᵢ(S}\boldsymbol{1)}$$

The sandwich estimator is used rather than the bootstrap because it is asymptotically valid under the Neyman orthogonality conditions of DML, does not require resampling of the full cross-fitting procedure (which would multiply computational cost by the number of bootstrap replications), and provides consistent standard errors even under heteroscedasticity and non-normal residuals (13).

For the binary treatment T₂ (coverage generosity), logistic regression with L₂ penalty (C = 0.1) was used for the propensity model E[T₂|X], preferred over GBM because it produces well-calibrated propensity scores on the moderate-dimensional covariate set and avoids the propensity-score extrapolation instability that can arise when a high-capacity model is applied to predict a coarsely defined binary treatment. Estimated propensity scores were trimmed to [0.05, 0.95] to enforce the overlap assumption and exclude episodes for which the counterfactual coverage scenario is empirically unsupported (14). Augmented Inverse Probability Weighting was applied for T₂ because it achieves the semiparametric efficiency bound and remains consistent if either the outcome or propensity model is correctly specified (but not necessarily both), providing double robustness (11).

For the binary treatments T₁ and T₃ (prolonged LOS and high service intensity), the FWL regression was applied directly to continuous residuals from a GBM first stage. T₃ was defined as an indicator for episodes in the top quartile (≥75th percentile) of the transaction-count distribution, yielding a prevalence of approximately 25% by construction; this dichotomisation strategy is formally justified by Swanson and Hernan (2013) (38), who demonstrate that binarisation of a continuous exposure at a substantively meaningful threshold produces a valid ATE estimand. The upper-quartile threshold was selected on three grounds: (i) epidemiological convention for defining “high utilisation” in administrative claims data (15) (16); (ii) adequate positivity — a 25% marginal prevalence avoids the near-violation of the overlap assumption arising from extreme quantile definitions such as top-decile cutoffs (17) ; and (iii) clinical interpretability, identifying episodes with a substantially elevated number of billing claim lines (≥4 per episode).

An overfit guard was implemented for all nuisance models: if AUC-ROC (binary) or R² (continuous) on the held-out fold exceeded 0.95, the model was refitted with a shallower specification (max_depth = 1, min_samples_leaf = 80) to prevent near-zero treatment residual variance that would cause the FWL denominator Σᵢν̂ᵢ² to collapse and inflate the ATE estimate. First-stage diagnostics additionally flagged treatment residual variance Var(ν̂) < 0.02 as indicative of weak treatment variation after conditioning on covariates, a continuous-treatment analogue of the weak-instrument condition (18); such estimates were reported as exploratory. Placebo permutation tests (199 permutations of T residuals) were conducted for each treatment–outcome pair as a non-parametric falsification check. Covariate leakage was prevented by excluding Y₁ (recovery) from the nuisance covariate set for Y₂, and all raw string columns whose label-encoded counterparts were already included. Inference used 95% Wald confidence intervals (z = 1.96) and two-sided p-values.

Causal identification rests on three core assumptions. Conditional ignorability (Y(t) ⊥ T | X for all t) requires that all confounders of the treatment–outcome relationship are captured in the observed covariate vector X. This assumption is supported by the breadth of the administrative covariate set — encompassing clinical severity, insurance type, provider identity, geographic location, demographic characteristics, and prior utilisation — but cannot be empirically verified in observational data; Rosenbaum bounds (S6 Appendix) quantify the degree of unobserved confounding that would be required to nullify each finding. Overlap (0 < P(T=1|X) < 1 for all X) was enforced by propensity-score trimming for T₂ and verified diagnostically for T₁ and T₃ via inspection of the first-stage R² and treatment residual variance. The stable unit treatment value assumption requires that a patient's potential outcome under treatment t is unaffected by the treatment received by other patients; this is plausible given that episodes are drawn from six geographically distinct provinces with limited documented inter-facility patient transfer, though it would be violated if hospital occupancy-level capacity constraints caused treated patients to displace untreated patients, a mechanism that was not testable with the available data.

**S3 Appendix. Stage III: Heterogeneous Treatment Effects via Causal Forest**

Causal Forest, implemented via the Generalised Random Forest (GRF) framework (19, 20), was selected over alternative HTE estimators for reasons specifically tied to the study's objectives and data structure. First, Causal Forest extends the DML partialling-out step of Stage II to local, non-parametric estimation via an adaptive nearest-neighbour kernel: trees are split to maximise heterogeneity in treatment effect rather than prediction accuracy, making the algorithm inherently oriented toward the causal quantity of interest. Second, compared to parametric interaction regression, Causal Forest discovers heterogeneity driven by any covariate combination or higher-order interaction without requiring the analyst to pre-specify which variables modify treatment response; in a dataset with 20 engineered features, the number of two-way interaction terms alone (¹⁰C₂ = 190) exceeds the effective degrees of freedom available for reliable parametric estimation. Third, GRF's honest estimation — whereby each tree builds its splits on a training subsample and evaluates leaf estimates on a disjoint estimation subsample — provably eliminates in-sample overfitting of the CATE surface, producing asymptotically valid confidence intervals (19). Fourth, T-learner and S-learner meta-learners were considered as alternatives; however, the T-learner produces biased CATEs when treatment group sample sizes are unequal (as is the case for all three treatments in this study) (21), and the S-learner is known to shrink heterogeneity estimates toward zero when the treatment indicator has small marginal importance relative to the full covariate set. GRF's weighting by treatment residuals ν̂ᵢ² explicitly corrects for this shrinkage.

The forest specification comprised 1,000 trees, minimum leaf size 5, and square-root feature subsampling. The 1,000-tree specification follows the GRF default recommended for samples of n ≈ 1,800, providing sufficient variance reduction without excessive computational cost (22) ; pilot experiments with 500 and 2,000 trees confirmed that CATE point estimates and standard errors were stable beyond 800 trees. The minimum leaf size of 5 ensures that each leaf contains at least 5 treated and 5 control observations, preventing degenerate variance estimates in the leaf-level CATE calculations. Pseudo-outcomes Γ̃ᵢ = ε̂ᵢ/ν̂ᵢ were computed from Stage II residuals (clipped at the 1st–99th percentile to remove leverage points from extreme treatment residuals), weighted by ν̂ᵢ² to down-weight observations with poor treatment variation.

Out-of-bag (OOB) R² was computed for each model as a data-driven signal-strength criterion. When OOB R² fell below the pre-specified threshold of 0.02 — indicating that the forest cannot predict pseudo-outcomes above chance, and therefore that patient-level CATE variation is indistinguishable from sampling noise — patient-level CATE was replaced by pre-specified subgroup ATEs from 11 clinically defined groups (three age categories × two severity tiers × rural/urban × SES strata) estimated within the Stage II FWL/AIPW framework. This decision rule prevents false claims of individual-level heterogeneity when signal-to-noise is insufficient, following the recommendation to validate meta-learner predictions against a null benchmark before interpreting CATE heterogeneity (21). For models retaining patient-level CATEs, heterogeneity was formally tested using the Best Linear Predictor (BLP) regression:

$$\boldsymbol{Yᵢ - m̂(Xᵢ) = \alpha₀ \cdot(Tᵢ - ê(Xᵢ)) + \alpha₁ \cdot(Tᵢ - ê(Xᵢ)) \cdot(\taû(Xᵢ) - ATE) + \xiᵢ(S}\boldsymbol{2)}$$

A significant α₁ (t-test, α = 0.05) indicates that τ̂(Xᵢ) carries genuine heterogeneity signal beyond the mean effect (41). The BLP test was preferred over CLAN and GATES tests because it is a single-degree-of-freedom test, more powerful than multi-group GATES for detecting aggregate heterogeneity when its direction is unknown, and does not require pre-specifying the number of subgroups. Ninety-percent bootstrap confidence intervals (300 replications, random-forest resampling with replacement) were computed for patient-level CATEs; 90% rather than 95% intervals were adopted following the GRF convention (19), reflecting that OOB-based CIs for individual predictions have higher variance than population-mean CIs.

**S4 Appendix. Stage IV: Equity Measurement**

The Concentration Index was selected as the primary inequality measure rather than the Gini coefficient or Theil index for three reasons specific to the study's equity objective (15). First, unlike the Gini coefficient, the CI simultaneously captures both the magnitude of outcome inequality and its socioeconomic direction (CI > 0 indicates pro-rich concentration, CI < 0 indicates pro-poor), which is essential for determining whether wealthier patients are receiving disproportionately more or less care. Second, unlike income-based Gini measures, the CI ranks individuals by their socioeconomic position rather than by the outcome itself, making it appropriate for measuring socioeconomic-related health disparities rather than pure outcome dispersion (15). Third, the CI has direct and well-established relationships to horizontal inequity measurement and to the Wagstaff–van Doorslaer decomposition framework, providing methodological coherence across the equity measurement, standardisation, and attribution components of Stage IV.

Three SES proxies were used: insurance fund type (primary, most theoretically justified given the Iranian multi-payer system where fund type is a direct proxy for employer-based socioeconomic position), a composite SES rank index, and rural/urban residence. For binary outcomes, the Erreygers (2009) (23) normalised index was applied,

$$\boldsymbol{CI\_E = 4}\boldsymbol{\mu\cdot CI(S}\boldsymbol{3)}$$

correcting for the mathematical property that the range of the standard CI is constrained by the outcome mean in [0,1]-bounded variables, rendering cross-group comparisons invalid without normalisation (23, 24). Bootstrap confidence intervals (1,000 replications, bias-corrected and accelerated — BCa; DiCiccio and Efron, 1996) were computed for all estimates. BCa bootstrap was preferred over the standard percentile bootstrap and normal approximation because it corrects for both the median bias and the skewness of the bootstrap distribution of the CI statistic, which is particularly important when the SES distribution is itself skewed (as is the case with insurance fund type in the Iranian system).

Horizontal inequity (HI) — the component of inequality not attributable to legitimate differences in health need — was estimated by the indirect standardisation method (25). A need-standardised expected outcome Ŷᵢneed was generated by regressing Yᵢ on the legitimate need variables (age, sex, severity score) using logistic regression for binary outcomes and OLS for continuous outcomes; logistic regression was used for the prediction step because it correctly preserves the [0,1] probability bounds for binary outcomes, which OLS does not. HI = CI(Yᵢ) − CI(Ŷᵢneed); a significant HI > 0 implies that better-off patients receive more care than their health need warrants. Indirect rather than direct standardisation was used because it produces a HI measure interpretable as the inequality remaining after removing the portion 'explained' by legitimate need variation, and because direct standardisation requires applying counterfactual need distributions across SES groups that may have unrealistic support in the data(25).

The decomposition attributed (15) the CI to determinant contributions via a Linear Probability Model (LPM; OLS on binary Y) rather than logistic regression:

***Σₖ ηₖ CIₖ + GC(ε)/μ = CI*	(S4)**

LPM is used specifically because it guarantees that this decomposition identity holds exactly; logistic partial effects do not have the linearity property required by the identity, so estimated contributions from a logistic decomposition do not sum to the observed CI, producing an unquantifiable residual(25). This is a deliberate methodological choice: the LPM is used solely for the decomposition arithmetic (where exact additivity is required) while logistic regression serves the prediction role (where probability bounds matter). The squared age term was excluded from the decomposition after diagnostics identified near-collinearity with age and disease severity in the LPM, inflating elasticity estimates. The primary SES variable was excluded from its own CI decomposition to avoid the circularity of using SES rank to explain itself (26). Decompositions were flagged as unreliable when |GC(ε)|/|CI| > 1.0 (residual dominates observed inequality) or the additive gap exceeded 0.01.

**S5 Appendix. Stage V: Constrained Welfare Optimisation**

Binary Integer Linear Programming (ILP) was selected as the optimisation framework over continuous relaxation and greedy heuristics for three reasons aligned with the study's decision problem. First, treatment decisions in this context are inherently binary (a patient either receives the treatment modality or does not), making the binary variable domain the correct representation of the decision space; a continuous relaxation would produce fractional treatment assignments that are not interpretable as actionable policy recommendations. Second, ILP with CBC branch-and-bound (27) guarantees a globally optimal solution within the 1% MIP gap tolerance, whereas greedy descent provides only a local optimum; given that the equity constraint introduces a global coupling between high- and low-severity allocation rates, local greedy methods can systematically under-allocate to severity groups that are collectively sub-optimal even when individually they appear beneficial. Third, the budget constraints are linear in the binary decision variables, making the problem exactly representable as an ILP without approximation. The CBC solver was chosen over commercial alternatives (CPLEX) because it is open-source and reproducible without institutional licensing, and benchmarks on problems of this scale (n ≈ 1,800 binary variables, 3 constraints) show CBC achieves the 1% gap within 120 seconds reliably (27).

The welfare function maximised (main text, Eq 6) was subject to the following budget-neutral constraints:

$$\boldsymbol{\Sigmaᵢ \Delta\_LOSᵢ \cdot tᵢ \leq B\_LOS (bed-day budget = observed system total)(S}\boldsymbol{5)}$$

$$\boldsymbol{\Sigmaᵢ \Delta\_InsCostᵢ \cdot tᵢ \leq B\_cost (insurer cost budget = observed system total)(S}\boldsymbol{6)}$$

$$\boldsymbol{n\_treated(high-sev) / N\_high \geq n\_treated(low-sev) / N\_low (equity constraint)(S}\boldsymbol{7)}$$

The utilitarian welfare function is the canonical choice for health-economic resource allocation analysis (28) and is appropriate here because the study's objective is to identify the allocation that maximises total population health gain (weighted by α) net of total patient financial burden (weighted by λ), not to maximise the welfare of any individual. The welfare weights α = λ = 1 in the base case represent a neutral social planner indifferent between one unit of health gain and one unit of out-of-pocket reduction; the 4 × 4 sensitivity grid (α, λ ∈ {0.5, 1, 2, 5}) spans the range of plausible societal preferences from health-maximising to out-of-pocket-minimising. The patient-level health gain τᵢ(xᵢ) was set to the Stage III CATE for recovery (Y₁), falling back to the negated readmission-proxy CATE where Y₁ subgroup ATEs were degenerate.

The budget constraints were defined as budget-neutral relative to observed system totals, reflecting the policy-relevant question of how to reallocate existing resources rather than requesting additional funding. Incremental insurer cost Δ_InsCostᵢ was computed from first principles by multiplying each patient's observed total cost by the difference between the counterfactual and observed coverage ratios, rather than from the population-constant expected-cost auxiliary variable (which was found diagnostically to equal the dataset mean for all patients, producing an infeasible budget ratio of 4.7× observed spending). The hard equity constraint encodes the need principle — high-severity patients should be treated at least as frequently as low-severity patients — following the ethical framework and consistent with the Iranian health system's stated equity objectives(29). A welfare-descending greedy heuristic incorporating the same constraint served as both a warm-start for CBC and a robustness comparison. Three policy scenarios were evaluated: (A) observed practice, (B) welfare-optimal allocation under the equity constraint, and (C) equity-maximising allocation (α = 0, λ = −1). Allocation equity was measured by the CI of the binary allocation vector.

**S6 Appendix. Stage VI: Uncertainty Quantification**

A four-layer architecture was adopted to separately address statistically distinct sources of uncertainty, each requiring a different propagation method.

**Layer 1** — Causal effect confidence intervals. ATEs and mean-CATEs from Stage III were accompanied by analytical 95% confidence intervals derived from the influence-function sandwich standard errors. The analytical approach is used rather than a bootstrap of the full DML–Causal Forest pipeline because (i) the sandwich SE is asymptotically exact under the GRF's estimating equations and does not require resampling assumptions about the cross-fitting distribution, (ii) re-running the full pipeline 1,000 times would accumulate Monte Carlo noise from the TPE hyperparameter search and the 5-fold CV splits, introducing variance not present in the original estimate, and (iii) the influence-function approach is computationally tractable at n = 1,813 where full bootstrap would require ≈1,000 re-fits of six GBM models (30). Subgroup ATEs were accompanied by bias-corrected bootstrap confidence intervals (1,000 replications, normal approximation recentred on the observed estimate, SE taken as the maximum of the bootstrap SE and the Stage III analytical SE), providing conservative uncertainty bounds when the bootstrap distribution is asymmetric.

**Layer 2** — Equity index confidence intervals. Bootstrap confidence intervals (1,000 replications, BCa correction (31) were computed for all Concentration Indices and HI measures. The BCa bootstrap was selected over the delta method because the delta-method approximation for the CI's covariance-based estimator assumes that the sampling distribution of Cov(Y, r) is normal, which is violated when outcome distributions are skewed (as in cost and length-of-stay outcomes); the BCa correction adjusts for both bias and skewness in the bootstrap distribution without requiring distributional assumptions.

**Layer 3** — Sensitivity to unobserved confounding. Rosenbaum (2002) (32) sensitivity bounds were derived for each causal model using the Wilcoxon signed-rank statistic applied to DML residual pseudo-pairs (ε̂ᵢ, ν̂ᵢ), computing the critical Γ — the minimum unobserved odds ratio of treatment assignment that would invalidate the finding at α = 0.05. The Wilcoxon signed-rank statistic was selected over the sign test and the Hodges–Lehmann estimator because it uses the full information in the magnitude of residuals (not just their sign) and is more powerful against alternatives involving continuous effect distributions. A direction-aware p-value was applied (32): lower-tail for negative ATEs (confounding would need to explain a protective effect), upper-tail for positive ATEs (confounding would need to explain a harmful effect); this directionality avoids the anti-conservative error of using upper-tail p-values for negative ATEs, which would overstate robustness. The critical Γ was reported as the study's primary sensitivity summary: a Γ > 1.5 is conventionally interpreted as indicating moderate robustness to hidden bias (32).

**Layer 4** — Welfare conclusion uncertainty. Bootstrap uncertainty in CATEs was propagated into the welfare optimisation by perturbing each patient's CATE by a draw from N(0, SE_subgroup²) (500 replications) and re-solving the greedy allocation problem in each draw. Five hundred replications were chosen to achieve a Monte Carlo standard error on the 2.5th percentile of the welfare distribution below 1% of the interquartile range, verified in a pilot run; 1,000 replications provided negligible additional precision. The greedy solver was used for computational tractability (500 full ILP re-solves would require ≈10 hours; greedy produces near-identical allocation patterns to ILP for the base-case scenario, verified in the main analysis). The resulting 95% credible intervals for total welfare, treatment rates, and severity-targeting ratio were reported. If the lower bound of the severity-targeting ratio confidence interval exceeded 1.0, the equity property of the allocation was declared robust to CATE uncertainty.

**S7 Appendix. Key Assumptions, Identification Conditions, and Limitations**

Causal identification in Stages II–III rests on three core assumptions. Conditional ignorability (Y(t) ⊥ T | X for all t) requires that all confounders of the treatment–outcome relationship are captured in the observed covariate vector X. This assumption is supported by the breadth of the administrative covariate set — encompassing clinical severity, insurance type, provider identity, geographic location, demographic characteristics, and prior utilisation — but cannot be empirically verified in observational data; Rosenbaum bounds (S6 Appendix, Layer 3) quantify the degree of unobserved confounding that would be required to nullify each finding. Overlap (0 < P(T=1|X) < 1 for all X) was enforced by propensity-score trimming [0.05, 0.95] for T₂ and verified diagnostically for T₁ and T₃ via inspection of the first-stage R² and treatment residual variance; the upper-quartile definition of T₃ was specifically designed to ensure a 25% marginal treatment prevalence that supports the overlap assumption across all major covariate subgroups. SUTVA (stable unit treatment value assumption) requires that a patient's potential outcome under treatment t is unaffected by the treatment received by other patients. This is plausible given that episodes are drawn from six geographically distinct provinces with limited documented inter-facility patient transfer, reducing the likelihood of spillover effects through bed capacity or provider substitution; it would be violated if hospital occupancy-level capacity constraints caused treated patients to displace untreated patients, a mechanism that was not testable with the available data.

Three additional limitations bear noting. First, the welfare optimisation in Stage V is normative rather than causal: it identifies the allocation maximising welfare given the estimated CATEs under the stated preference function, but it does not predict what outcomes would actually result from implementing a new allocation policy, because implementation may induce behavioural responses from providers, patients, and insurers that alter the CATEs themselves. Second, the episode-level unit of analysis means that multiple admissions for the same patient are treated as independent observations; the magnitude of this dependence is unknown without a patient linkage key, but sensitivity analyses excluding same-day discharges (flagged by same_day_flag) were pre-specified to probe robustness of cost and length-of-stay estimates to the most procedurally distinct episode type. Third, the six-province administrative dataset may not be nationally representative of Parkinson's disease care in Iran, limiting the external validity of the welfare and equity conclusions to settings with similar multi-payer insurance structures and urban–rural gradients.

**A:**

**S1 Figure. Descriptive Epidemiology of Parkinson’s Disease Episodes: Length of Stay, Age Distribution, Temporal Trends, Insurance Coverage, and Cost by Discharge Status**

**S1Table.Variance Inflation Factor (VIF) Analysis for Multicollinearity Among initial variables**

| **Variable** | **VIF** |
| --- | --- |
| total_requested | 335.5631 |
| total_paid | 316.3822 |
| total_cost | 21.42038 |
| los_days | 5.158915 |
| insured_share | 4.180155 |
| admission_dow | 2.685369 |
| is_weekend_admit | 2.677766 |
| n_transactions | 2.631554 |
| cost_per_day | 1.921278 |
| patient_oop_ratio | 1.900814 |
| coverage_ratio | 1.858414 |
| same_day_flag | 1.452121 |
| admission_year | 1.450204 |
| admission_month | 1.32603 |
| age | 1.066614 |

*Note: Variables with VIF > 10 (total_requested, total_paid, total_cost) indicate high multicollinearity, guiding regularization or exclusion in the ML framework.*

**S1 Table.** Variance Inflation Factor (VIF) analysis identified severe multicollinearity among the three aggregate expenditure variables (*total_requested*, *total_paid*, and *total_cost*; VIFs 21.4–335.6), reflecting their close accounting relationship. All remaining predictors showed low to moderate collinearity (VIF < 6), indicating that multicollinearity was not a concern for the core clinical, utilization, and demographic variables. Accordingly, the highly collinear cost variables were excluded or regularized within the machine learning framework to improve model stability while retaining the remaining predictors for subsequent causal and predictive analyses.

**S2 Figure. Correlation Matrix Heatmap of Key Cost and initial Variables (Pearson Coefficients)**

**Stage 1**

Fold-level out-of-fold metrics for the two binary Stage 1 models are reported in S2 Table to allow assessment of estimate stability across the five cross-validation folds underlying Table 3. AUC-ROC for Y1 ranged from 0.675 to 0.763 across folds and for Y2 from 0.836 to 0.926, with no single fold disproportionately responsible for the pooled estimates.

**S2 Table. Fold-level five-fold cross-validation performance for the Stage 1 binary outcome models (recovery, Y1; discharge-risk proxy, Y2).**

| **Outcome** | **Fold** | **AUC-ROC** | **AUC-PR** | **Brier score** | **Log-loss** |
| --- | --- | --- | --- | --- | --- |
| Y1 — Recovery | 1 | 0.741 | 0.887 | 0.161 | 0.494 |
| Y1 — Recovery | 2 | 0.694 | 0.857 | 0.166 | 0.509 |
| Y1 — Recovery | 3 | 0.763 | 0.902 | 0.153 | 0.471 |
| Y1 — Recovery | 4 | 0.675 | 0.859 | 0.171 | 0.521 |
| Y1 — Recovery | 5 | 0.702 | 0.866 | 0.158 | 0.495 |
| Y2 — Discharge-risk proxy | 1 | 0.868 | 0.542 | 0.208* | 0.608 |
| Y2 — Discharge-risk proxy | 2 | 0.854 | 0.424 | 0.167* | 0.521 |
| Y2 — Discharge-risk proxy | 3 | 0.911 | 0.680 | 0.127* | 0.430 |
| Y2 — Discharge-risk proxy | 4 | 0.836 | 0.468 | 0.155* | 0.495 |
| Y2 — Discharge-risk proxy | 5 | 0.926 | 0.787 | 0.119* | 0.409 |

*AUC-ROC, area under the receiver-operating-characteristic curve; AUC-PR, average precision. *Fold-level Brier scores for Y2 are reported prior to the isotonic recalibration applied to produce the pooled, calibrated Brier score in Table 3 (0.074); log-loss is likewise computed on raw (pre-calibration) predicted probabilities.*

Fold-to-fold variability was modest for both outcomes given the sample size (n = 1,813 across five folds, approximately 363 per fold), and no fold showed AUC-ROC or Brier-score values that were outliers relative to the remaining four, supporting the stability of the pooled out-of-fold estimates reported in Table 3. The wider range in average precision for Y2 (0.424–0.787) is attributable to the small absolute number of positive cases per fold (approximately 43, given an 11.9% base rate), which is an inherent property of cross-validating a rare-outcome model rather than evidence of an unstable predictor set.

Fold-level metrics for the three continuous/count outcome models are reported in S3 Table; R² for the log-cost model (Y3) remained stable across folds (range 0.801–0.841), as did R² for the out-of-pocket-ratio (Y4; range 0.269–0.335) and length-of-stay (Y5; range 0.235–0.407) models.

**S3 Table. Fold-level five-fold cross-validation performance for the Stage 1 continuous and count outcome models (log-total cost, Y3; patient out-of-pocket ratio, Y4; length of stay, Y5).**

| **Outcome** | **Fold** | **RMSE** | **MAE** | **R²** |
| --- | --- | --- | --- | --- |
| Y3 — log-total cost | 1 | 0.489 | 0.364 | 0.804 |
| Y3 — log-total cost | 2 | 0.450 | 0.351 | 0.841 |
| Y3 — log-total cost | 3 | 0.499 | 0.388 | 0.808 |
| Y3 — log-total cost | 4 | 0.504 | 0.375 | 0.808 |
| Y3 — log-total cost | 5 | 0.514 | 0.386 | 0.801 |
| Y4 — Patient OOP ratio | 1 | 0.067 | 0.044 | 0.269 |
| Y4 — Patient OOP ratio | 2 | 0.063 | 0.044 | 0.335 |
| Y4 — Patient OOP ratio | 3 | 0.070 | 0.047 | 0.323 |
| Y4 — Patient OOP ratio | 4 | 0.063 | 0.045 | 0.274 |
| Y4 — Patient OOP ratio | 5 | 0.070 | 0.048 | 0.292 |
| Y5 — Length of stay | 1 | 5.667 | 3.260 | 0.235 |
| Y5 — Length of stay | 2 | 4.146 | 2.569 | 0.379 |
| Y5 — Length of stay | 3 | 4.917 | 2.977 | 0.355 |
| Y5 — Length of stay | 4 | 4.871 | 2.807 | 0.383 |
| Y5 — Length of stay | 5 | 4.773 | 2.857 | 0.407 |

*RMSE, root-mean-squared error; MAE, mean absolute error; R², coefficient of determination. Y3 metrics are on the natural-log scale, log(1 + total_cost), and are currency-invariant; see Table 4 footnote for the purchasing-power-parity convention applied wherever absolute cost values are reported elsewhere in the manuscript.*

As with the binary outcomes, fold-level performance for the continuous and count models was consistent across the five cross-validation folds, with no single fold driving the pooled R² estimates reported in Table 4. The comparatively wider fold range observed for Y5 (length of stay; R² 0.235–0.407) is consistent with the greater dispersion and right-skew typical of hospital length-of-stay data and does not indicate model instability, since RMSE and MAE varied correspondingly and proportionately across the same folds.

Hyperparameters selected via an objective-specific Tree-structured Parzen Estimator search (50 trials per outcome) are reported in S4 Table. All five outcome models converged on a shared core configuration (600 boosting rounds, learning rate 0.03, maximum tree depth 4, 75% row and column subsampling, minimum child weight 10, L1/L2 regularisation of 0.5/2.0), differing only in their outcome-appropriate objective function and, for Y2, a class-imbalance weight (scale_pos_weight = 7.39) reflecting its 11.9% positive-class prevalence.

**S4 Table. Selected hyperparameters for the Stage 1 gradient-boosting models, by outcome.**

| **Outcome** | **Objective** | **Eval. metric** | **n_est.** | **LR** | **Depth** | **Subs.** | **Cols.** | **Min ch. wt.** | **L1/L2 reg.** | **Outcome-specific parameter** |
| --- | --- | --- | --- | --- | --- | --- | --- | --- | --- | --- |
| Y1 — Recovery | binary:logistic | AUC | 600 | 0.03 | 4 | 0.75 | 0.75 | 10 | 0.5 / 2.0 | scale_pos_weight = 1.00 |
| Y2 — Discharge-risk proxy | binary:logistic | AUC | 600 | 0.03 | 4 | 0.75 | 0.75 | 10 | 0.5 / 2.0 | scale_pos_weight = 7.39 |
| Y3 — log-total cost | reg:squarederror | RMSE | 600 | 0.03 | 4 | 0.75 | 0.75 | 10 | 0.5 / 2.0 | — |
| Y4 — Patient OOP ratio | reg:tweedie | Tweedie neg. log-lik. (p=1.5) | 600 | 0.03 | 4 | 0.75 | 0.75 | 10 | 0.5 / 2.0 | tweedie_variance_power = 1.5 |
| Y5 — Length of stay | count:poisson | Poisson neg. log-lik. | 600 | 0.03 | 4 | 0.75 | 0.75 | 10 | 0.5 / 2.0 | — |

*All models used 40-round early stopping, the histogram tree method, and random_state = 42. LR, learning rate; Subs., subsample fraction; Cols., colsample_bytree; Min ch. wt., minimum child weight; L1/L2 reg., reg_alpha / reg_lambda; RMSE, root-mean-squared error; OOP, out-of-pocket; wt., weight.*

The convergence of independently tuned models on a common core hyperparameter configuration, differing only in outcome-appropriate loss functions, indicates that a single moderately regularised gradient-boosting architecture is well suited across the heterogeneous outcome types (binary, continuous, Tweedie-distributed ratio, and count) examined in this study, and that the performance differences reported in Tables 3 and 4 reflect genuine differences in the predictability of the underlying outcomes rather than differences in model capacity or tuning effort. The class-imbalance weighting applied to Y2 (scale_pos_weight = 7.39) is proportionate to its observed prevalence and was applied only at the training stage, prior to the isotonic recalibration reported in Table 3.

The extended (top-ten) predictor sets underlying Table 5 are reported in S5 Table, confirming that administrative case-mix and treatment-related features, rather than the severity score alone, drive predictive performance across all five outcomes.

**S5 Table. Extended (top ten, rank-ordered) predictors retained by each Stage 1 outcome model.**

| **Outcome** | **Top ten predictors (rank 1 → 10)** |
| --- | --- |
| Y1 — Recovery | age; age_sq; T3_high_intensity; cost_per_day; private_hospital; state_enc; same_day_flag; physician_specialty_enc; ownership_enc; T2_high_coverage |
| Y2 — Discharge-risk proxy | case_type_enc; n_transactions; T1_prolonged_los; ownership_enc; state_enc; rural_x_coverage; severity_score; physician_specialty_enc; cost_per_day; coverage_ratio |
| Y3 — log-total cost | case_type_enc; T1_prolonged_los; same_day_flag; T3_high_intensity; service_group_enc; private_hospital; T2_high_coverage; coverage_ratio; ownership_enc; insurance_fund_enc |
| Y4 — Patient OOP ratio | case_type_enc; same_day_flag; ownership_enc; T1_prolonged_los; insurance_fund_enc; admission_year; state_enc; rural_flag; n_transactions; severity_score |
| Y5 — Length of stay | case_type_enc; T3_high_intensity; n_transactions; coverage_ratio; physician_specialty_enc; T2_high_coverage; service_group_enc; ownership_enc; state_enc; private_hospital |

*_enc denotes a label-encoded categorical administrative descriptor. T1_prolonged_los, T2_high_coverage and T3_high_intensity are the three treatment variables of primary policy interest; severity_score is the admission-time-only, cross-fitted case-mix score used for causal adjustment in Stage 2 (Table 2).*

Reviewing the full top-ten predictor sets confirms the pattern summarised in Table 5: encoded administrative descriptors (case type, ownership, state, physician specialty, service group) and the three treatment variables account for the majority of predictive contribution across outcomes, while the severity score and coverage ratio contribute at only a secondary rank (sixth-to-tenth position) for the discharge-risk-proxy and out-of-pocket-ratio models. No post-treatment outcome variable (length of stay, total cost, or discharge status) was used as an input feature for its own outcome model, and the admission-time-only severity score was never among the top-five predictors of any outcome, consistent with its pre-treatment construction (Table 2) and with the identification strategy applied in Stage 2.

**S3 Figure. Cross-Validation Performance Across 10 Folds: Binary AUC (Readmission Proxy & Recovery) and Continuous RMSE (LOS, Log Total Cost, OOP Burden Ratio)**

**S4 Figure. Feature-Level Interpretability: Mean Gain Importance, Predicted Probabilities, Top 15 Feature Importances, and Top 15 SHAP Contributions (Last Fold)**

**S5 Figure. Summary Distribution of Performance Metrics Across Folds: ROC-AUC, Precision-Recall AUC, Calibration, and SHAP/Importance Ranges (Min, Q1, Median, Q3, Max)**

**S6 Figure. Model Fit and Feature Attribution for Log Total Cost**

**S7Figure. Model Fit and Feature Attribution for LOS days**

**S8 Figure. Model Fit and Feature Attribution for OOP Burden Ratio**

**Stage 2**

The T3 outcome (cost) nuisance model, E[Y|X], was estimated using a distinct 18-covariate set that reinstates the transaction count omitted from the T3 propensity model (Table 6), so that predictive information relevant to cost is retained in the outcome model without compromising identification of the treatment model.

**S4 Table. Covariate set used for the outcome nuisance model E[Y|X] under the T3 high-transaction-intensity specification.**

| **Treatment specification** | **Nuisance target** | **No. of covariates** | **Covariates** |
| --- | --- | --- | --- |
| T3 — High transaction intensity | E[Y\|X] only (Y3 log-cost) | 18 | age, age², admission year, admission month, admission season, weekend-admission flag, severity score, rural-facility flag, private-hospital flag, sex, state, insurance fund, case type, ownership type, physician specialty, age × severity interaction, female × rural interaction, number of billed transactions |

Reinstating the transaction count in the outcome model but not the treatment model is a deliberate identification-protecting asymmetry: including it in E[T|X] would make the propensity model a near-tautological predictor of the exposure it is meant to explain, whereas including it in E[Y|X] improves out-of-fold prediction of cost without affecting the orthogonality condition used to identify the ATE. The practical consequence, noted in Table 7, is that the T3–Y3 ATE (≈+5.4%, not statistically significant) should be read as a conservative estimate: to the extent that any true causal effect of high transaction intensity on cost operates through transaction volume itself, that pathway is partly absorbed by the outcome model rather than attributed to the treatment effect, biasing the estimate toward the null.

A continuous specification of T3 (transaction count rather than a binary high/low indicator) was estimated as a secondary robustness check. This specification produced markedly larger ATEs than the primary binary specification — a 22.0-percentage-point reduction in the discharge proxy (ATE = −0.220, 95% CI −0.298 to −0.142, p < 0.001) and an implausibly large cost effect equivalent to roughly a ten-fold (≈+1,045%) increase (ATE = 2.437, 95% CI 2.257 to 2.617, p < 0.001) — alongside a propensity (first-stage) model that explained only 41.6% of the variance in the continuous exposure (R² = 0.416).

**S5 Table. Sensitivity analysis: DML ATE estimates and nuisance diagnostics for the continuous T3 (transaction-intensity) specification.**

| **Outcome** | | **ATE (SE)** | | **95% CI** | | **p-value** | |
| --- | --- | --- | --- | --- | --- | --- | --- |
| Unsuccessful-discharge proxy (Y2) | | −0.220 (0.040) | | −0.298, −0.142 | | <0.001 | |
| Total cost, log scale (Y3) | | 2.437 (0.092) | | 2.257, 2.617 | | <0.001 | |
| **Nuisance target** | **Model** | **AUC-ROC** | **Accuracy** | **RMSE** | **MAE** | | **R²** |
| Treatment (continuous) | E[T\|X] | — | — | 0.214 | 0.162 | | 0.416 |
| Y2 discharge proxy | E[Y\|X] | 0.823 | 0.895 | — | — | | — |
| Y3 log-cost | E[Y\|X] | — | — | 0.799 | 0.630 | | 0.504 |

This specification is presented only as an exploratory robustness check and is explicitly not the headline T3 estimate. Two features of the diagnostics motivate this caution: the propensity model for the continuous exposure explains less than half of its variance (R² = 0.416, versus AUC-ROC 0.72 for the binary specification's propensity model), indicating a comparatively weak first stage and a correspondingly higher risk of finite-sample bias and inflated effect sizes in the residual-on-residual estimator; and the resulting cost ATE (an approximate ten-fold proportional increase) is implausibly large for a real-world health-financing effect. Both the direction of the discharge-proxy effect and the direction of the cost effect are consistent with the primary binary specification, which supports the qualitative pattern reported in Table 7, but the magnitudes here should not be used for quantitative or policy interpretation.

The T1 discharge-proxy ATE remained negative and statistically significant across every length-of-stay percentile threshold tested, from the 40th to the 75th percentile (ATE range −0.068 to −0.036, all p < 0.01), with the treated-group prevalence ranging from 51.0% to 21.7% as the threshold was tightened.

**S6 Table. Threshold robustness of the prolonged-LOS ATE on the unsuccessful-discharge proxy (Y2) across alternative percentile cut-offs.**

| **LOS percentile threshold** | **Treated prevalence** | **ATE** | **SE** | **p-value** |
| --- | --- | --- | --- | --- |
| 40th | 51.0% | −0.068 | 0.013 | <0.001 |
| 50th (primary) | 40.2% | −0.065 | 0.012 | <0.001 |
| 60th | 32.1% | −0.042 | 0.012 | <0.001 |
| 70th | 26.0% | −0.044 | 0.013 | <0.001 |
| 75th | 21.7% | −0.036 | 0.014 | 0.008 |

The effect is stable in sign and remains statistically significant regardless of where the prolonged-LOS threshold is drawn, with a modest decline in magnitude as the definition is tightened (from −6.8 to −3.6 percentage points). This indicates that the association is not an artefact of a single, arbitrarily chosen cut-off. It does not, however, address the endogeneity concern raised under Table 6/7: because the finding is robust across thresholds but the severity-score adjustment remains a plausibly post-treatment/outcome-related covariate for this specification, the result continues to be reported as a robust associational pattern rather than a robust causal effect.

The T1 cost ATE was similarly stable across thresholds, ranging from 0.814 to 0.829 log-points (all p < 0.001), corresponding to an approximate 125.6–129.1% increase in total cost associated with prolonged length-of-stay regardless of the specific percentile used to define "prolonged."

**S7 Table. Threshold robustness of the prolonged-LOS ATE on total cost (Y3, log scale) across alternative percentile cut-offs.**

| **LOS percentile threshold** | **Treated prevalence** | **ATE** | **SE** | **p-value** | **Approx. effect** |
| --- | --- | --- | --- | --- | --- |
| 40th | 51.0% | 0.829 | 0.025 | <0.001 | ≈+129.1% |
| 50th (primary) | 40.2% | 0.814 | 0.025 | <0.001 | ≈+125.6% |
| 60th | 32.1% | 0.822 | 0.025 | <0.001 | ≈+127.6% |
| 70th | 26.0% | 0.818 | 0.026 | <0.001 | ≈+126.6% |
| 75th | 21.7% | 0.828 | 0.027 | <0.001 | ≈+129.0% |

The cost association is essentially invariant to the choice of length-of-stay threshold, both in statistical significance and in magnitude (a narrow ≈125–129% band across a near-doubling of the implied treated-group size). As with S6 Table, this rules out threshold selection as an explanation for the finding but leaves the associational, rather than causal, interpretation unchanged for the reasons set out under Table 6/7.

Unlike the T1 results, the T3 discharge-proxy ATE was not robust to threshold choice: the effect stayed negative in direction across all six percentile cut-offs tested but reached conventional statistical significance only at the most extreme (90th percentile) threshold (ATE = −0.032, p = 0.032), remaining non-significant (p = 0.33–0.39) at all more moderate thresholds, consistent with the null primary-specification result in Table 7.

**S8 Table. Threshold robustness of the high-transaction-intensity ATE on the unsuccessful-discharge proxy (Y2) across alternative percentile cut-offs.**

| **Transaction-intensity percentile threshold** | **Treated prevalence** | **ATE** | **SE** | **p-value** |
| --- | --- | --- | --- | --- |
| 50th | 52.4% | −0.012 | 0.013 | 0.357 |
| 60th | 52.4% | −0.012 | 0.013 | 0.357 |
| 70th | 38.1% | −0.011 | 0.012 | 0.391 |
| 75th (primary) | 26.5% | −0.013 | 0.013 | 0.326 |
| 80th | 26.5% | −0.013 | 0.013 | 0.326 |
| 90th | 10.1% | −0.032 | 0.015 | 0.032 |

The consistently negative but largely non-significant pattern across thresholds supports treating the T3-to-discharge-proxy relationship as inconclusive rather than as an established null or an established effect; the single significant result at the extreme 90th-percentile cut-off, based on the smallest treated subgroup (10.1% prevalence), is more likely to reflect reduced statistical power at moderate thresholds combined with a genuinely small effect than a threshold-specific artefact, but it should not be over-interpreted given the multiple comparisons involved in this sweep.

The T3 cost ATE showed a similar borderline pattern: effects were positive and of modest magnitude (≈+5.4% to +10.4%) at every threshold, reaching nominal significance at the 50th, 60th and 90th percentiles (p = 0.009–0.037) but not at the 70th–80th percentiles (p = 0.068–0.096), including the primary 75th-percentile specification reported in Table 7.

**S9 Table. Threshold robustness of the high-transaction-intensity ATE on total cost (Y3, log scale) across alternative percentile cut-offs.**

| **Transaction-intensity percentile threshold** | **Treated prevalence** | **ATE** | **SE** | **p-value** | **Approx. effect** |
| --- | --- | --- | --- | --- | --- |
| 50th | 52.4% | 0.068 | 0.033 | 0.037 | ≈+7.0% |
| 60th | 52.4% | 0.068 | 0.033 | 0.037 | ≈+7.0% |
| 70th | 38.1% | 0.056 | 0.031 | 0.068 | ≈+5.8% |
| 75th (primary) | 26.5% | 0.052 | 0.031 | 0.096 | ≈+5.4% |
| 80th | 26.5% | 0.052 | 0.031 | 0.096 | ≈+5.4% |
| 90th | 10.1% | 0.099 | 0.038 | 0.009 | ≈+10.4% |

Taken with S8 Table, this pattern indicates a small, borderline-significant positive association between high transaction intensity and cost that is directionally consistent but statistically fragile across plausible threshold choices. Combined with the primary (Table 7) and continuous (S5 Table) specifications, the overall weight of evidence for T3 is that any causal effect on cost, if present, is modest in size and should be described in the manuscript with corresponding hedged language rather than as a confirmed policy-relevant effect.

**Stage 3**

The tables below provide the full methodological detail underlying the DAG-based confounder specification, the positivity/overlap diagnostics, the nuisance-model and robustness diagnostics, the complete subgroup breakdowns for all six exposure–outcome pairs, and the covariate-importance rankings summarised in the main text (Tables 9–12).

S10 Table lists the full inventory of 38 nodes in the prespecified DAG together with their assigned causal role. Twenty variables fixed at or before admission are declared confounders common to all three exposures and both outcomes; three variables are the treatments under study; thirteen variables that are realised during or after the hospitalisation are declared mediators (post-treatment, non-confounder) rather than adjustment covariates; and two variables are the study outcomes.

**S10 Table. Full node inventory of the prespecified causal directed acyclic graph, by causal role.**

| **Variable** | **DAG role** |
| --- | --- |
| age | Confounder (pre-treatment) |
| gender_enc | Confounder (pre-treatment) |
| case_type_enc | Confounder (pre-treatment) |
| physician_specialty_enc | Confounder (pre-treatment) |
| service_group_enc | Confounder (pre-treatment) |
| state_enc | Confounder (pre-treatment) |
| rural_flag | Confounder (pre-treatment) |
| private_hospital | Confounder (pre-treatment) |
| ownership_enc | Confounder (pre-treatment) |
| insurance_fund_enc | Confounder (pre-treatment) |
| admission_month | Confounder (pre-treatment) |
| admission_season | Confounder (pre-treatment) |
| admission_year | Confounder (pre-treatment) |
| is_weekend_admit | Confounder (pre-treatment) |
| year_flag | Confounder (pre-treatment) |
| ses_rank | Confounder (pre-treatment) |
| severity_score | Confounder (pre-treatment) |
| age_sq | Confounder (pre-treatment) |
| age_x_severity | Confounder (pre-treatment) |
| female_x_rural | Confounder (pre-treatment) |
| T1_prolonged_los | Treatment / exposure |
| T2_high_coverage | Treatment / exposure |
| T3_continuous | Treatment / exposure |
| los_days | Mediator (post-treatment) |
| cost_per_day | Mediator (post-treatment) |
| total_cost | Mediator (post-treatment) |
| coverage_ratio | Mediator (post-treatment) |
| same_day_flag | Mediator (post-treatment) |
| adverse_discharge | Mediator (post-treatment) |
| recovery_flag | Mediator (post-treatment) |
| n_transactions | Mediator (post-treatment) |
| service_count | Mediator (post-treatment) |
| claim_line_count | Mediator (post-treatment) |
| episode_length | Mediator (post-treatment) |
| Y2_readmission_proxy (unsuccessful-discharge proxy) | Outcome |
| Y3_log_total_cost | Outcome |

*All 20 confounders were linked by a confounding edge to each of the 3 treatments and both outcomes (100 confounding edges in total); the treatment-to-mediator and mediator-to-outcome edges are enumerated in S11 Table.*

S11 Table enumerates every treatment→mediator, mediator→outcome, and treatment→outcome edge in the prespecified DAG. These are the edges of substantive interest for the endogeneity concern raised during review: they show explicitly that length of stay, cost, discharge status, and claims-activity counts are modelled as consequences of treatment (or as the outcome itself), and never as confounders feeding back into treatment assignment.

**S11 Table. Post-treatment (mediator) and outcome edges of the prespecified causal directed acyclic graph.**

| **Source** | **Target** | **Edge type** |
| --- | --- | --- |
| T1_prolonged_los | los_days | Post-treatment path (mediator) |
| T1_prolonged_los | cost_per_day | Post-treatment path (mediator) |
| T1_prolonged_los | total_cost | Post-treatment path (mediator) |
| T1_prolonged_los | adverse_discharge | Post-treatment path (mediator) |
| T1_prolonged_los | recovery_flag | Post-treatment path (mediator) |
| T1_prolonged_los | same_day_flag | Post-treatment path (mediator) |
| T1_prolonged_los | Y2_readmission_proxy | Estimated effect (associational, Table 11) |
| T1_prolonged_los | Y3_log_total_cost | Estimated effect (associational, Table 11) |
| T2_high_coverage | coverage_ratio | Post-treatment path (mediator) |
| T2_high_coverage | cost_per_day | Post-treatment path (mediator) |
| T2_high_coverage | total_cost | Post-treatment path (mediator) |
| T2_high_coverage | adverse_discharge | Post-treatment path (mediator) |
| T2_high_coverage | recovery_flag | Post-treatment path (mediator) |
| T2_high_coverage | same_day_flag | Post-treatment path (mediator) |
| T2_high_coverage | Y2_readmission_proxy | Estimated effect (causal, conditional exchangeability, Table 11) |
| T2_high_coverage | Y3_log_total_cost | Estimated effect (causal, conditional exchangeability, Table 11) |
| T3_continuous | service_count | Post-treatment path (mediator) |
| T3_continuous | claim_line_count | Post-treatment path (mediator) |
| T3_continuous | episode_length | Post-treatment path (mediator) |
| T3_continuous | cost_per_day | Post-treatment path (mediator) |
| T3_continuous | total_cost | Post-treatment path (mediator) |
| T3_continuous | same_day_flag | Post-treatment path (mediator) |
| T3_continuous | recovery_flag | Post-treatment path (mediator) |
| T3_continuous | adverse_discharge | Post-treatment path (mediator) |
| T3_continuous | Y2_readmission_proxy | Estimated effect (exploratory, Table 11) |
| T3_continuous | Y3_log_total_cost | Mechanical, flagged non-causal (Table 11) |
| n_transactions | T3_continuous | Definitional (mediator is a determinant of the treatment definition) |
| same_day_flag | Y2_readmission_proxy | Definitional component of the outcome proxy |
| adverse_discharge | Y2_readmission_proxy | Definitional component of the outcome proxy |
| recovery_flag | Y2_readmission_proxy | Definitional component of the outcome proxy |

*The 20 confounder→treatment and confounder→outcome edges (100 edges) are omitted from this listing for brevity and summarised in S10 Table; source data are identical to those validated in Table 9.*

S12 Table reports the full positivity/overlap diagnostics for the treatment-assignment (propensity or first-stage residual) models underlying each doubly-robust estimator. The binary coverage exposure (T2) showed a well-behaved propensity-score distribution (5th–95th percentile: 0.33–0.66) with 0% of observations requiring clipping. The continuous exposures (T1, T3) are evaluated via the distribution of absolute first-stage residuals; T3 showed a heavier concentration of near-zero residuals (5th percentile = 0.014, versus 0.078 for T1), consistent with weaker first-stage variation and motivating its exploratory label in Table 11.

**S12 Table. Positivity and overlap diagnostics for the treatment-assignment models used in the Step 3 estimators.**

| **Treatment** | **Diagnostic** | **Min** | **P1** | **P5** | **P25** | **Median** | **P75** | **P95** | **P99** | **Max** | **Clip thresh.** | **% trimmed** | **N treated / control** |
| --- | --- | --- | --- | --- | --- | --- | --- | --- | --- | --- | --- | --- | --- |
| T1 (prolonged LOS) | \|First-stage residual\| | 0.001 | 0.016 | 0.078 | 0.314 | 0.443 | 0.573 | 0.743 | 0.846 | 1.105 | 0.001 | 0.06% | — |
| T2 (high coverage) | Propensity score | 0.204 | 0.261 | 0.328 | 0.431 | 0.504 | 0.575 | 0.663 | 0.714 | 0.795 | 0.05 | 0.00% | 906 / 907 |
| T3 (treatment intensity) | \|First-stage residual\| | <0.001 | 0.002 | 0.014 | 0.060 | 0.123 | 0.212 | 0.384 | 0.560 | 0.980 | 0.0135 | 5.02% | — |

*Percentiles (P1–P99) are of the fitted propensity score (T2) or of the absolute residual from the first-stage regression of treatment on the pre-treatment covariate set (T1, T3). “% trimmed” is the share of observations below the clipping threshold applied prior to inverse-weighting.*

S13 Table reports, for all six exposure–outcome pairs, the nuisance-model fit diagnostics for the treatment model E(T∣X) and outcome model E(Y∣X), the E-values, and the explicit disclosure of the repeated-measures limitation: the underlying claims extract carries no patient identifier, so multiple admissions by the same patient cannot be linked, patient-level independence is not assumed, and a separate design-effect sensitivity script (within_patient_dependence_sensitivity.py) was run to bound the influence of unlinked repeated admissions on the standard errors reported in Table 11.

**S13 Table. Nuisance-model diagnostics, E-values, and repeated-measures disclosure for all six exposure–outcome pairs.**

| **Exposure** | **Outcome** | **E(T\|X) metric (score)** | **E(Y\|X) metric (score)** | **E-value (point/CI)** | **Patient ID available** | **Patient-level independence assumed** |
| --- | --- | --- | --- | --- | --- | --- |
| T1 | Y2 | R² (0.056, poor fit) | AUC-ROC (0.83) | 1.68 / 1.49 | No | No |
| T1 | Y3 | R² (0.056, poor fit) | R² (0.52, poor fit) | 4.25 / 4.04 | No | No |
| T2 | Y2 | AUC-ROC (0.59, poor fit) | AUC-ROC (0.83) | 1.32 / 1.00 | No | No |
| T2 | Y3 | AUC-ROC (0.59, poor fit) | R² (0.52, poor fit) | 2.37 / 2.19 | No | No |
| T3 | Y2 | R² (0.51, poor fit) | AUC-ROC (0.83) | 3.13 / 2.35 | No | No |
| T3 | Y3 | R² (0.51, poor fit) | R² (0.52, poor fit) | n/a (mechanical) | No | No |

*“Poor fit” flags nuisance models whose cross-validated score fell below the prespecified adequacy threshold; a poor-fitting nuisance model widens the plausible range of residual bias in the corresponding doubly-robust estimate and is reported rather than concealed. Design-effect-adjusted, clustering-robust confidence intervals from the dedicated sensitivity script are reported separately from the main analysis and should be consulted alongside Table 11 wherever within-patient dependence is a concern for a specific estimate.*

S14 Table reports the complete subgroup breakdown for prolonged length of stay (T1) on both the discharge-risk proxy (Y2) and log total cost (Y3), across age band, clinical severity tier, hospital sector, rurality, and socioeconomic status, underlying the condensed comparison in Table 12 (T1, T2) or reported here in full for completeness (T3).

**S14 Table. Subgroup treatment-effect estimates for prolonged length of stay (T1) on the discharge-risk proxy (Y2) and log total cost (Y3).**

| **Subgroup** | **n** | **ATE, Y2 (95% CI)** | **p (Y2)** | **ATE, Y3 log-scale (95% CI)** | **p (Y3)** |
| --- | --- | --- | --- | --- | --- |
| Age < 65 | 489 | -0.086 (-0.130, -0.042) | <0.001 | 1.092 (0.982, 1.202) | <0.001 |
| Age 65–74 | 567 | -0.043 (-0.081, -0.006) | 0.024 | 1.113 (1.011, 1.215) | <0.001 |
| Age ≥ 75 | 757 | -0.064 (-0.101, -0.028) | <0.001 | 1.094 (1.015, 1.174) | <0.001 |
| Low severity | 871 | -0.076 (-0.109, -0.043) | <0.001 | 1.022 (0.940, 1.103) | <0.001 |
| High severity | 942 | -0.053 (-0.084, -0.021) | 0.001 | 1.170 (1.096, 1.243) | <0.001 |
| Private hospital | 100 | -0.066 (-0.131, -0.001) | 0.045 | 0.609 (0.443, 0.775) | <0.001 |
| Public hospital | 1713 | -0.064 (-0.087, -0.040) | <0.001 | 1.132 (1.075, 1.188) | <0.001 |
| Rural | 547 | -0.063 (-0.099, -0.027) | <0.001 | 1.117 (1.012, 1.222) | <0.001 |
| Urban | 1266 | -0.064 (-0.093, -0.035) | <0.001 | 1.092 (1.028, 1.155) | <0.001 |
| High SES | 1813 | -0.064 (-0.086, -0.041) | <0.001 | 1.099 (1.045, 1.154) | <0.001 |

*ATE for Y2 is a risk difference on the discharge-risk proxy (proportion scale); ATE for Y3 is on the natural-log scale of total cost. p-values as originally computed (not multiplicity-adjusted); subgroups with n below the prespecified minimum of 30 were skipped and are omitted.*

S15 Table reports the complete subgroup breakdown for high insurance-coverage tier (T2) on both the discharge-risk proxy (Y2) and log total cost (Y3), across age band, clinical severity tier, hospital sector, rurality, and socioeconomic status, underlying the condensed comparison in Table 12 (T1, T2) or reported here in full for completeness (T3).

**S15 Table. Subgroup treatment-effect estimates for high insurance-coverage tier (T2) on the discharge-risk proxy (Y2) and log total cost (Y3).**

| **Subgroup** | **n** | **ATE, Y2 (95% CI)** | **p (Y2)** | **ATE, Y3 log-scale (95% CI)** | **p (Y3)** |
| --- | --- | --- | --- | --- | --- |
| Age < 65 | 489 | -0.001 (-0.057, 0.050) | 0.966 | -0.488 (-0.615, -0.348) | <0.001 |
| Age 65–74 | 567 | 0.013 (-0.033, 0.056) | 0.578 | -0.469 (-0.588, -0.322) | <0.001 |
| Age ≥ 75 | 757 | 0.043 (0.005, 0.091) | 0.055 | -0.547 (-0.656, -0.445) | <0.001 |
| Low severity | 871 | 0.005 (-0.034, 0.041) | 0.776 | -0.489 (-0.585, -0.391) | <0.001 |
| High severity | 942 | 0.037 (-0.002, 0.077) | 0.072 | -0.523 (-0.621, -0.416) | <0.001 |
| Private hospital | 100 | 0.045 (-0.058, 0.152) | 0.393 | -0.257 (-0.463, -0.031) | 0.026 |
| Public hospital | 1713 | 0.020 (-0.007, 0.050) | 0.155 | -0.521 (-0.599, -0.445) | <0.001 |
| Rural | 547 | -0.000 (-0.049, 0.042) | 0.988 | -0.512 (-0.657, -0.355) | <0.001 |
| Urban | 1266 | 0.031 (-0.008, 0.060) | 0.068 | -0.504 (-0.583, -0.425) | <0.001 |
| High SES | 1813 | 0.022 (-0.005, 0.049) | 0.120 | -0.507 (-0.572, -0.438) | <0.001 |

*ATE for Y2 is a risk difference on the discharge-risk proxy (proportion scale); ATE for Y3 is on the natural-log scale of total cost. p-values as originally computed (not multiplicity-adjusted); subgroups with n below the prespecified minimum of 30 were skipped and are omitted.*

S16 Table reports the complete subgroup breakdown for treatment/claims intensity (T3, exploratory / mechanical—see Table 11 footnote) on both the discharge-risk proxy (Y2) and log total cost (Y3), across age band, clinical severity tier, hospital sector, rurality, and socioeconomic status, underlying the condensed comparison in Table 12 (T1, T2) or reported here in full for completeness (T3).

**S16 Table. Subgroup treatment-effect estimates for treatment/claims intensity (T3, exploratory / mechanical—see Table 11 footnote) on the discharge-risk proxy (Y2) and log total cost (Y3).**

| **Subgroup** | **n** | **ATE, Y2 (95% CI)** | **p (Y2)** | **ATE, Y3 log-scale (95% CI)** | **p (Y3)** |
| --- | --- | --- | --- | --- | --- |
| Age < 65 | 489 | -0.156 (-0.331, 0.019) | 0.081 | 2.463 (2.139, 2.787) | <0.001 |
| Age 65–74 | 567 | -0.176 (-0.293, -0.059) | 0.003 | 2.520 (2.211, 2.828) | <0.001 |
| Age ≥ 75 | 757 | -0.312 (-0.434, -0.191) | <0.001 | 2.589 (2.317, 2.861) | <0.001 |
| Low severity | 871 | -0.179 (-0.294, -0.065) | 0.002 | 2.385 (2.135, 2.635) | <0.001 |
| High severity | 942 | -0.273 (-0.378, -0.169) | <0.001 | 2.710 (2.483, 2.937) | <0.001 |
| Private hospital | 100 | -0.205 (-0.380, -0.030) | 0.022 | 1.672 (1.290, 2.054) | <0.001 |
| Public hospital | 1713 | -0.222 (-0.306, -0.138) | <0.001 | 2.598 (2.418, 2.778) | <0.001 |
| Rural | 547 | -0.220 (-0.337, -0.103) | <0.001 | 2.700 (2.377, 3.023) | <0.001 |
| Urban | 1266 | -0.222 (-0.323, -0.121) | <0.001 | 2.455 (2.253, 2.657) | <0.001 |
| High SES | 1813 | -0.221 (-0.300, -0.142) | <0.001 | 2.529 (2.357, 2.702) | <0.001 |

*ATE for Y2 is a risk difference on the discharge-risk proxy (proportion scale); ATE for Y3 is on the natural-log scale of total cost. p-values as originally computed (not multiplicity-adjusted); subgroups with n below the prespecified minimum of 30 were skipped and are omitted.*

S17 Table reports the five most influential covariates in the outcome-nuisance model for each of the six exposure–outcome pairs. The interaction term age_x_severity and the severity index itself were consistently the two most important covariates, followed by age, its square, and the state fixed effect, across all six pairs — a pattern consistent with the covariate set adjusting predominantly for demographic and administrative risk profile rather than for the treatment-adjacent quantities excluded from the DAG (S11 Table).

**S17 Table. Top five most influential pre-treatment covariates in the outcome-nuisance model, by exposure–outcome pair.**

| **Exposure** | **Outcome** | **Rank 1** | **Rank 2** | **Rank 3** | **Rank 4** | **Rank 5** |
| --- | --- | --- | --- | --- | --- | --- |
| T1 | Y2 | age_x_severity (0.135) | severity_score (0.131) | age_sq (0.106) | age (0.104) | state_enc (0.103) |
| T1 | Y3 | severity_score (0.144) | age_x_severity (0.142) | state_enc (0.116) | age (0.099) | age_sq (0.099) |
| T2 | Y2 | age_x_severity (0.146) | severity_score (0.127) | state_enc (0.117) | age (0.104) | age_sq (0.103) |
| T2 | Y3 | age_x_severity (0.160) | severity_score (0.130) | state_enc (0.125) | age_sq (0.111) | age (0.110) |
| T3 | Y2 | age_x_severity (0.144) | severity_score (0.130) | age (0.109) | state_enc (0.109) | age_sq (0.108) |
| T3 | Y3 | age_x_severity (0.143) | severity_score (0.125) | state_enc (0.116) | age (0.106) | age_sq (0.103) |

*Values in parentheses are relative feature-importance scores from the cross-fitted outcome-nuisance learner (sum to 1 across all 20 covariates within each exposure–outcome pair). Full importances for all 20 covariates and all six pairs are available from the analysis code repository.*

**S9 Figure. Prespecified Causal Directed Acyclic Graph (DAG) for Parkinson's Hospitalisation Episodes**

**Stage 4**

S18 Table reports the sample sizes underlying the two categorical stratifications used for subgroup reporting in this manuscript (Table 12; Tables 13–15), to make explicit which strata are well powered and which are small.

**S18 Table. Sample sizes underlying the rurality and hospital-sector stratifications.**

| **Stratifying variable** | **Level** | **n** | **% of analytic sample** |
| --- | --- | --- | --- |
| Rurality | Urban (rural_flag = 0) | 1266 | 69.8% |
| Rurality | Rural (rural_flag = 1) | 547 | 30.2% |
| Hospital sector | Public (private_hospital = 0) | 1713 | 94.5% |
| Hospital sector | Private (private_hospital = 1) | 100 | 5.5% |

*n = 1,813 episodes total. The private-hospital stratum (n = 100, 5.5% of the sample) is the smallest subgroup reported anywhere in this manuscript and is flagged as low precision wherever it appears (Table 12; S14–S16 Tables).*

S19 Table reports the pairwise rank correlations among the three candidates socioeconomic-status proxies used in this manuscript. Insurance-fund type and the composite SES rank are strongly correlated (ρ = 0.74), and insurance-fund type and rural residence are moderately correlated (ρ = 0.63), whereas the composite SES rank and rural residence are only weakly correlated (ρ = 0.05). Any determinant whose correlation with the active ranking variable met or exceeded the prespecified threshold of |ρ| = 0.60 was treated as quasi-circular and excluded from the decomposition reported in Table 15 and S20 Table.

**S19 Table. Pairwise correlations among candidate socioeconomic-status ranking variables.**

| **Variable 1** | **Variable 2** | **Spearman ρ** | **Action taken** |
| --- | --- | --- | --- |
| Composite SES rank | Rural residence | 0.051 | Below threshold; both retained as independent robustness checks (Table 13) |
| Composite SES rank | Insurance fund type | 0.739 | Above threshold; not used jointly as ranking + determinant |
| Insurance fund type (primary ranking variable) | Rural residence | 0.629 | Above threshold; rural residence excluded as a decomposition determinant when insurance fund type is the ranking variable (Table 15) |

*Threshold for exclusion as a quasi-circular determinant: |ρ| ≥ 0.60 against the ranking variable used for that decomposition. Rurality and the composite SES rank remain available as independent ranking variables in their own right (Table 13) even though rurality cannot also serve as a determinant within the insurance-fund-type decomposition.*

S20 Table reports the complete Wagstaff–van Doorslaer decomposition underlying the summary in Table 15: the elasticity of each outcome with respect to each determinant, the determinant's own concentration index, its resulting contribution to the outcome's concentration index, and (where the outcome's reliability tier supports it) that contribution's share of the total.

**S20 Table. Full decomposition of the concentration index (insurance-fund type) into determinant-level contributions, by outcome.**

| **Outcome** | **Determinant** | **Elasticity (η)** | **Determinant's C** | **Contribution** | **% of total C** | **Tier** |
| --- | --- | --- | --- | --- | --- | --- |
| Recovery (Y1) | Sex | 0.0078 | −0.0040 | −0.00003 | −0.1% | Reliable |
| Recovery (Y1) | Severity score | −0.1083 | −0.0164 | +0.0018 | +6.2% | Reliable |
| Recovery (Y1) | Age | −0.4665 | −0.0261 | +0.0122 | +42.4% | Reliable |
| Recovery (Y1) | Private hospital | 0.0093 | −0.4865 | −0.0045 | −15.7% | Reliable |
| Recovery (Y1) | Admission season | 0.0106 | −0.0175 | −0.0002 | −0.6% | Reliable |
| Recovery (Y1) | Residual | — | — | +0.0195 | +67.8% | Reliable |
| Recovery (Y1) | Total decomposed (observed C = +0.0287)ᵃ | — | — | +0.0287 | 100% | Reliable |
| Unsuccessful-discharge proxy (Y2) | Sex | 0.0079 | −0.0040 | −0.00003 | n/rᵇ | Unreliable |
| Unsuccessful-discharge proxy (Y2) | Severity score | 0.1656 | −0.0164 | −0.0027 | n/rᵇ | Unreliable |
| Unsuccessful-discharge proxy (Y2) | Age | −0.4028 | −0.0261 | +0.0105 | n/rᵇ | Unreliable |
| Unsuccessful-discharge proxy (Y2) | Private hospital | −0.0263 | −0.4865 | +0.0128 | n/rᵇ | Unreliable |
| Unsuccessful-discharge proxy (Y2) | Admission season | −0.0876 | −0.0175 | +0.0015 | n/rᵇ | Unreliable |
| Unsuccessful-discharge proxy (Y2) | Residual | — | — | −0.0677 | n/rᵇ | Unreliable |
| Unsuccessful-discharge proxy (Y2) | Total decomposed (observed C = −0.0456)ᵃ | — | — | −0.0456 | 100%ᶜ | Unreliable |
| Log total cost (Y3) | Sex | 0.0021 | −0.0040 | −0.00001 | +0.1% | Reliable |
| Log total cost (Y3) | Severity score | −0.0030 | −0.0164 | +0.00005 | −0.8% | Reliable |
| Log total cost (Y3) | Age | 0.0482 | −0.0261 | −0.0013 | +19.7% | Reliable |
| Log total cost (Y3) | Private hospital | 0.0023 | −0.4865 | −0.0011 | +17.8% | Reliable |
| Log total cost (Y3) | Admission season | 0.0025 | −0.0175 | −0.00004 | +0.7% | Reliable |
| Log total cost (Y3) | Residual | — | — | −0.0040 | +62.5% | Reliable |
| Log total cost (Y3) | Total decomposed (observed C = −0.0064)ᵃ | — | — | −0.0064 | 100% | Reliable |
| Out-of-pocket ratio (Y4) | Sex | 0.0114 | −0.0040 | −0.00005 | Indicativeᵈ | Borderline |
| Out-of-pocket ratio (Y4) | Severity score | −0.1212 | −0.0164 | +0.0020 | Indicativeᵈ | Borderline |
| Out-of-pocket ratio (Y4) | Age | −0.1194 | −0.0261 | +0.0031 | Indicativeᵈ | Borderline |
| Out-of-pocket ratio (Y4) | Private hospital | 0.0144 | −0.4865 | −0.0070 | Indicativeᵈ | Borderline |
| Out-of-pocket ratio (Y4) | Admission season | −0.0237 | −0.0175 | +0.0004 | Indicativeᵈ | Borderline |
| Out-of-pocket ratio (Y4) | Residual | — | — | −0.0549 | Indicativeᵈ | Borderline |
| Out-of-pocket ratio (Y4) | Total decomposed (observed C = −0.0565)ᵃ | — | — | −0.0565 | 100% | Borderline |
| Length of stay (Y5) | Sex | 0.0122 | −0.0040 | −0.00005 | +0.1% | Reliable |
| Length of stay (Y5) | Severity score | 0.0874 | −0.0164 | −0.0014 | +3.1% | Reliable |
| Length of stay (Y5) | Age | 0.4619 | −0.0261 | −0.0121 | +26.0% | Reliable |
| Length of stay (Y5) | Private hospital | 0.0029 | −0.4865 | −0.0014 | +3.1% | Reliable |
| Length of stay (Y5) | Admission season | −0.0037 | −0.0175 | +0.0001 | −0.1% | Reliable |
| Length of stay (Y5) | Residual | — | — | −0.0315 | +67.9% | Reliable |
| Length of stay (Y5) | Total decomposed (observed C = −0.0464)ᵃ | — | — | −0.0464 | 100% | Reliable |

*ᵃThe decomposition is computed on the standard (non-Erreygers) concentration index; for Recovery (Y1), the standard-index total of +0.0287 reproduces the Erreygers-corrected index of +0.0874 reported in Table 13 after the Erreygers boundary correction for a bounded outcome, and both are internally consistent (residual audit gap ≈ 0 for every outcome; not shown). ᵇn/r: not reported. For the discharge-risk proxy the residual (−0.068) exceeds the observed concentration index (−0.046) in absolute value, so individual percentage shares would be uninterpretable (a share can exceed 100% or flip sign) and are withheld; the raw contributions are shown for transparency only. ᶜShown as 100% by construction (contributions plus residual sum to the observed index) and should not be read as evidence that the decomposition is reliable. ᵈPercentage-of-total shares are not computed for the borderline-reliability out-of-pocket-ratio outcome; raw contributions are reported as approximate/indicative only, consistent with Table 15. Rural residence was excluded from every row as a determinant because of its collinearity with insurance-fund type (ρ = 0.63; S19 Table). Decomposition performed via a linear probability model with 500 bootstrap replications for all standard errors and confidence intervals in Tables 13–14; consistent with Table 11 and S13 Table, the underlying claims extract carries no patient identifier, so all intervals are computed at the episode level without adjustment for potential within-patient clustering.*

**S10 Figure. Wagstaff–van Doorslaer Concentration Index Decomposition for Length of Stay (Y5) — Absolute Determinant Contributions to Observed Pro-Poor Inequality (CI = −0.0502) via Linear Probability Model**

**S11 Figure. Wagstaff–van Doorslaer Concentration Index Decomposition for Out of Pocket (Y4) — Absolute Determinant Contributions to Observed Pro-Poor Inequality (CI = −0.0820) via Linear Probability Model**

**S12 Figure. Wagstaff–van Doorslaer Concentration Index Decomposition for Recovery (Y1) — Absolute Determinant Contributions to Observed Pro-Poor Inequality (CI = 0.0302) via Linear Probability Model**

**S13 Figure. Wagstaff–van Doorslaer Concentration Index Decomposition for Log Total Cost (Y3) — Absolute Determinant Contributions to Observed Pro-Poor Inequality (CI = −0.0061) via Linear Probability Model**

**S14 Figure. Concentration Curve for Length of Stay (Y5) Ranked by Insurance Fund Type as Primary Socioeconomic Proxy — Cumulative Outcome Share versus Cumulative Population Share with Pro-Rich and Pro-Poor Area**

**S15 Figure. Concentration Curve for Out of Pocket (Y4) Ranked by Insurance Fund Type as Primary Socioeconomic Proxy — Cumulative Outcome Share versus Cumulative Population Share with Pro-Rich and Pro-Poor Area**

**S16 Figure. Concentration Curve for Recovery (Y1) Ranked by Insurance Fund Type as Primary Socioeconomic Proxy — Cumulative Outcome Share versus Cumulative Population Share with Pro-Rich and Pro-Poor Area**

**S17 Figure. Concentration Curve for Readmission Proxy (Y2) Ranked by Insurance Fund Type as Primary Socioeconomic Proxy — Cumulative Outcome Share versus Cumulative Population Share with Pro-Rich and Pro-Poor Area**

**S18 Figure. Concentration Curve for Log Total Cost (Y3) Ranked by Insurance Fund Type as Primary Socioeconomic Proxy — Cumulative Outcome Share versus Cumulative Population Share with Pro-Rich and Pro-Poor Area**

**Stage 5**

S21-S23 Tables report the full (α, λ) weight-sensitivity analysis and the underlying budget-feasibility diagnostics for Stage 5. As in the main text, T2_high_coverage is the primary policy-relevant scenario and T1_prolonged_los is reported for descriptive comparison only; monetary values are converted to PPP-adjusted international dollars (Int$) at the approximate rate of 150,000 IRR/Int$ described in the main text.

S21 Table reports the full 16-cell (α, λ) sensitivity grid for the primary T2_high_coverage scenario under the significance-gated welfare objective, spanning a 10-fold range in the weight placed on health gain (α) and a 10-fold range in the weight placed on out-of-pocket cost reduction (λ). The treated share ranges from 23.8% to 27.9% across the grid, and a classification of emergent high-severity targeting (TRUE) occurs in only 3 of the 16 cells.

**S21 Table.** Full α/λ sensitivity grid for the primary scenario (T2_high_coverage, significance-gated objective).

| **α** | **λ** | **N treated** | **% treated** | **Welfare index (W)** | **LOS budget used (%)** | **Cost budget used (%)** | **corr(alloc, severity)** | **corr p-value** | **Targets high severity** | **Age-equity classification** |
| --- | --- | --- | --- | --- | --- | --- | --- | --- | --- | --- |
| 0.5 | 0.5 | 504 | 27.8 | 122.91 | 28.3 | 100.0 | -0.039 | 0.095 | FALSE | FAVOURS_LOW (inverted) |
| 0.5 | 1 | 506 | 27.9 | 120.33 | 28.5 | 100.0 | -0.057 | 0.015 | FALSE | FAVOURS_LOW (inverted) |
| 0.5 | 2 | 499 | 27.5 | 113.40 | 28.2 | 100.0 | -0.060 | 0.010 | FALSE | FAVOURS_LOW (inverted) |
| 0.5 | 5 | 466 | 25.7 | 97.29 | 26.1 | 93.3 | -0.065 | 0.006 | FALSE | FAVOURS_LOW (inverted) |
| 1 | 0.5 | 498 | 27.5 | 245.70 | 28.0 | 100.0 | -0.003 | 0.907 | FALSE | FAVOURS_LOW (inverted) |
| 1 | 1 | 504 | 27.8 | 245.82 | 28.3 | 100.0 | -0.039 | 0.095 | FALSE | FAVOURS_LOW (inverted) |
| 1 | 2 | 506 | 27.9 | 240.66 | 28.5 | 100.0 | -0.057 | 0.015 | FALSE | FAVOURS_LOW (inverted) |
| 1 | 5 | 497 | 27.4 | 220.67 | 27.8 | 100.0 | -0.065 | 0.006 | FALSE | FAVOURS_LOW (inverted) |
| 2 | 0.5 | 493 | 27.2 | 490.13 | 28.0 | 100.0 | 0.104 | <0.001 | TRUE | FAVOURS_HIGH (emergent) |
| 2 | 1 | 498 | 27.5 | 491.40 | 28.0 | 100.0 | -0.003 | 0.907 | FALSE | FAVOURS_LOW (inverted) |
| 2 | 2 | 504 | 27.8 | 491.64 | 28.3 | 100.0 | -0.039 | 0.095 | FALSE | FAVOURS_LOW (inverted) |
| 2 | 5 | 493 | 27.2 | 466.16 | 27.5 | 100.0 | -0.060 | 0.010 | FALSE | FAVOURS_LOW (inverted) |
| 5 | 0.5 | 432 | 23.8 | 1087.02 | 24.9 | 100.0 | 0.329 | <0.001 | TRUE | FAVOURS_HIGH (emergent) |
| 5 | 1 | 488 | 26.9 | 1216.04 | 27.9 | 100.0 | 0.124 | <0.001 | TRUE | FAVOURS_HIGH (emergent) |
| 5 | 2 | 496 | 27.4 | 1226.55 | 27.9 | 100.0 | 0.015 | 0.520 | FALSE | FAVOURS_LOW (inverted) |
| 5 | 5 | 504 | 27.8 | 1229.09 | 28.3 | 100.0 | -0.039 | 0.095 | FALSE | FAVOURS_LOW (inverted) |

*corr(alloc, severity) is the point-biserial correlation between treatment allocation and the composite (outcome-adjacent) severity score, reported here only as a secondary, exploratory diagnostic; it does not define the primary equity guarantee (Table 17). Age-equity classification uses the pre-treatment age (≥75y) protected variable.*

Allocation size for the primary scenario is more sensitive to λ (the cost-aversion weight) than to α: at fixed α, increasing λ from 0.5 to 5 narrows the treated share (e.g., from 27.8% to 25.7% at α=0.5), while increasing α at fixed λ changes the treated share only marginally. Emergent high-severity targeting (TRUE) is observed only at 3 of 16 grid points, all at high α relative to λ; the remaining cells are classified FAVOURS_LOW (inverted) or, in one case, EQUAL, indicating that any apparent severity targeting is weight-dependent and should not be reported as a general or robust property of the optimisation.

S22 Table summarises S21 Table, and the equivalent 16-cell grids for the exploratory T1 scenario and for the two alternative objective specifications (Y2_ONLY, MULTI_OUTCOME), across all 96 grid points evaluated.

**S22 Table.** Summary of the α/λ sensitivity analysis across objective specifications and treatment scenarios.

| **Objective mode** | **Scenario (treatment)** | **N cells** | **Cells targeting high severity (n)** | **Cells targeting (%)** | **Welfare index coefficient of variation** |
| --- | --- | --- | --- | --- | --- |
| Y2_ONLY | T1_prolonged_los | 16 | 0 | 0.0 | 0.865 |
| Y2_ONLY | T2_high_coverage | 16 | 0 | 0.0 | n/a (all-zero allocation) |
| MULTI_OUTCOME | T1_prolonged_los | 16 | 0 | 0.0 | 0.854 |
| MULTI_OUTCOME | T2_high_coverage | 16 | 0 | 0.0 | 0.856 |
| SIGNIFICANCE_GATED | T1_prolonged_los | 16 | 0 | 0.0 | 0.854 |
| SIGNIFICANCE_GATED | T2_high_coverage | 16 | 3 | 18.8 | 0.851 |

*"Cells targeting high severity" counts grid points classified TRUE for emergent high-severity targeting (secondary, exploratory severity-score diagnostic; see S21 Table).*

For the exploratory T1 scenario, 0% of grid cells favour high-severity patients under any of the three objective specifications, and the underlying patient-level causal-effect proxy for T1 has a coefficient of variation of approximately 0.04 (essentially flat across patients); no combination of α/λ weights can therefore produce emergent severity targeting for this scenario, and any apparent targeting observed elsewhere reflects the hard equity constraint's floor rather than the welfare function itself. For the primary T2 scenario, emergent high-severity targeting reaches 18.8% of grid cells only under the significance-gated specification, and only at specific, cost-averse corners of the grid (S21 Table); it is absent under both alternative specifications. This indicates that severity targeting is not a general or weight-robust property of the optimisation and should not be reported as such.

S23 Table reports the budget-feasibility diagnostics underlying the optimisation, expressed in PPP-adjusted international dollars. Extending either scenario to all 1,813 patients would require 9.6- to 10.4-fold the observed insurer-cost budget, so that, on cost grounds alone and treating patients in ascending order of cost, at most approximately 959 (T1) or 700 (T2) patients could be covered.

**S23 Table.** Budget-feasibility diagnostics for the Stage 5 optimisation (raw, unscaled costs).

| **Scenario (treatment)** | **Observed bed-days (total)** | **Observed insurer cost (Int$)** | **Raw incremental cost if all patients treated (Int$)** | **Multiple of observed budget** | **Approx. max patients treatable on cost grounds alone (of 1,813)** |
| --- | --- | --- | --- | --- | --- |
| T1_prolonged_los | 9,868.0 | 91,543 | 882,044 | 9.64× | ~959 |
| T2_high_coverage | 9,868.0 | 91,543 | 954,485 | 10.43× | ~700 |

*Costs converted from IRR to Int$ at the approximate rate of 150,000 IRR/Int$ (main text). Observed insurer cost and bed-days are system totals over all N=1,813 episodes; the median absolute out-of-pocket amount used to scale the out-of-pocket term in the welfare index is Int$30.9 (4,630,827 IRR).*

Raw, unscaled per-patient incremental costs are used directly in the integer-linear-programming budget constraint, rather than being rescaled to force a fixed target level of budget utilisation; the solver's budget constraint handles the resulting infeasibility of treating all patients by selecting fewer patients under each scenario. As a result, the treated shares reported in Tables 16, 18 and 20, and in S21 Table, reflect the estimated cost structure of each scenario as observed in the claims data, rather than an assumed or predetermined utilisation target.

**Stage 6**

The following tables report robustness checks, subgroup-level detail, equity results by socioeconomic proxy, and a comparison of the exact and heuristic solvers used to construct the welfare-optimisation confidence intervals in Table 23.

Whether the classification of patients as having a prolonged length of stay is sensitive to the percentile threshold used to define it was assessed by comparing the median-based definition used in the primary analysis with three alternative thresholds (S24 Table).

**S24 Table. Reclassification of the prolonged-length-of-stay exposure indicator under alternative percentile thresholds, relative to the median-based definition used in the primary analysis.**

| **Alternative threshold** | **% of cohort reclassified** | **N** |
| --- | --- | --- |
| 60th percentile | 8.05% | 1,813 |
| 75th percentile | 18.48% | 1,813 |
| 90th percentile | 30.89% | 1,813 |

Reclassification increased monotonically with the distance between the alternative threshold and the median, from 8.1% at the 60th percentile to 30.9% at the 90th percentile; re-estimating the average treatment effects under each alternative threshold requires re-running the upstream double-machine-learning and causal-forest models and was therefore treated as an item for future work rather than resolved within this uncertainty-quantification stage. Because the reclassification rate is already non-trivial at the nearest threshold examined, all exploratory results attached to the length-of-stay exposure (Tables 21–23) should be interpreted as conditional on the specific median-based cut-point used.

Subgroup-specific average treatment effects, disaggregated by age band, exploratory clinical-severity band, hospital sector, urbanicity, and socioeconomic-status proxy, are reported for all three exposures and both outcomes in S25 Table.

**S25 Table. Subgroup-specific bootstrap average treatment effects on the discharge-outcome proxy and on total treatment cost.**

| **Exposure (causal role)** | **Outcome** | **Subgroup** | **Equity variable type** | **n** | **ATE** | **95% CI (bias-corrected bootstrap)** | **95% CI excludes 0** |
| --- | --- | --- | --- | --- | --- | --- | --- |
| Prolonged length of stay (exploratory) | Unsuccessful-Discharge Proxy (Y2) | Age < 65 | Primary | 489 | −0.086 | −0.130 to −0.042 | Yes |
| Prolonged length of stay (exploratory) | Unsuccessful-Discharge Proxy (Y2) | Age 65–74 | Primary | 567 | −0.043 | −0.087 to 0.000 | No |
| Prolonged length of stay (exploratory) | Unsuccessful-Discharge Proxy (Y2) | Age ≥ 75 | n/a | 757 | −0.064 | −0.101 to −0.028 | Yes |
| Prolonged length of stay (exploratory) | Unsuccessful-Discharge Proxy (Y2) | Low severityᵍ | Exploratory, post-treatment | 871 | −0.076 | −0.111 to −0.041 | Yes |
| Prolonged length of stay (exploratory) | Unsuccessful-Discharge Proxy (Y2) | High severityᵍ | Exploratory, post-treatment | 942 | −0.053 | −0.084 to −0.021 | Yes |
| Prolonged length of stay (exploratory) | Unsuccessful-Discharge Proxy (Y2) | Private hospital | n/a | 100 | −0.066 | −0.131 to −0.001 | Yes |
| Prolonged length of stay (exploratory) | Unsuccessful-Discharge Proxy (Y2) | Public hospital | n/a | 1,713 | −0.064 | −0.088 to −0.039 | Yes |
| Prolonged length of stay (exploratory) | Unsuccessful-Discharge Proxy (Y2) | Rural | n/a | 547 | −0.063 | −0.099 to −0.027 | Yes |
| Prolonged length of stay (exploratory) | Unsuccessful-Discharge Proxy (Y2) | Urban | n/a | 1,266 | −0.064 | −0.093 to −0.035 | Yes |
| Prolonged length of stay (exploratory) | Unsuccessful-Discharge Proxy (Y2) | High SESʰ | n/a | 1,813 | −0.064 | −0.087 to −0.041 | Yes |
| Prolonged length of stay (exploratory) | Log total treatment cost (Y3) | Age < 65 | Primary | 489 | +1.092 | 0.929 to 1.255 | Yes |
| Prolonged length of stay (exploratory) | Log total treatment cost (Y3) | Age 65–74 | Primary | 567 | +1.113 | 0.984 to 1.242 | Yes |
| Prolonged length of stay (exploratory) | Log total treatment cost (Y3) | Age ≥ 75 | n/a | 757 | +1.094 | 1.015 to 1.174 | Yes |
| Prolonged length of stay (exploratory) | Log total treatment cost (Y3) | Low severityᵍ | Exploratory, post-treatment | 871 | +1.022 | 0.914 to 1.129 | Yes |
| Prolonged length of stay (exploratory) | Log total treatment cost (Y3) | High severityᵍ | Exploratory, post-treatment | 942 | +1.170 | 1.057 to 1.283 | Yes |
| Prolonged length of stay (exploratory) | Log total treatment cost (Y3) | Private hospital | n/a | 100 | +0.609 | 0.422 to 0.796 | Yes |
| Prolonged length of stay (exploratory) | Log total treatment cost (Y3) | Public hospital | n/a | 1,713 | +1.132 | 1.050 to 1.213 | Yes |
| Prolonged length of stay (exploratory) | Log total treatment cost (Y3) | Rural | n/a | 547 | +1.117 | 0.984 to 1.251 | Yes |
| Prolonged length of stay (exploratory) | Log total treatment cost (Y3) | Urban | n/a | 1,266 | +1.092 | 0.996 to 1.188 | Yes |
| Prolonged length of stay (exploratory) | Log total treatment cost (Y3) | High SESʰ | n/a | 1,813 | +1.099 | 1.021 to 1.178 | Yes |
| High insurance coverage (primary) | Unsuccessful-Discharge Proxy (Y2) | Age < 65 | Primary | 489 | −0.001 | −0.063 to 0.061 | No |
| High insurance coverage (primary) | Unsuccessful-Discharge Proxy (Y2) | Age 65–74 | Primary | 567 | +0.013 | −0.044 to 0.070 | No |
| High insurance coverage (primary) | Unsuccessful-Discharge Proxy (Y2) | Age ≥ 75 | n/a | 757 | +0.043 | −0.001 to 0.087 | No |
| High insurance coverage (primary) | Unsuccessful-Discharge Proxy (Y2) | Low severityᵍ | Exploratory, post-treatment | 871 | +0.005 | −0.039 to 0.050 | No |
| High insurance coverage (primary) | Unsuccessful-Discharge Proxy (Y2) | High severityᵍ | Exploratory, post-treatment | 942 | +0.037 | −0.008 to 0.082 | No |
| High insurance coverage (primary) | Unsuccessful-Discharge Proxy (Y2) | Private hospital | n/a | 100 | +0.045 | −0.065 to 0.155 | No |
| High insurance coverage (primary) | Unsuccessful-Discharge Proxy (Y2) | Public hospital | n/a | 1,713 | +0.020 | −0.012 to 0.053 | No |
| High insurance coverage (primary) | Unsuccessful-Discharge Proxy (Y2) | Rural | n/a | 547 | 0.000 | −0.052 to 0.051 | No |
| High insurance coverage (primary) | Unsuccessful-Discharge Proxy (Y2) | Urban | n/a | 1,266 | +0.031 | −0.008 to 0.070 | No |
| High insurance coverage (primary) | Unsuccessful-Discharge Proxy (Y2) | High SESʰ | n/a | 1,813 | +0.022 | −0.009 to 0.052 | No |
| High insurance coverage (primary) | Log total treatment cost (Y3) | Age < 65 | Primary | 489 | −0.488 | −0.712 to −0.265 | Yes |
| High insurance coverage (primary) | Log total treatment cost (Y3) | Age 65–74 | Primary | 567 | −0.469 | −0.643 to −0.294 | Yes |
| High insurance coverage (primary) | Log total treatment cost (Y3) | Age ≥ 75 | n/a | 757 | −0.547 | −0.653 to −0.441 | Yes |
| High insurance coverage (primary) | Log total treatment cost (Y3) | Low severityᵍ | Exploratory, post-treatment | 871 | −0.489 | −0.630 to −0.349 | Yes |
| High insurance coverage (primary) | Log total treatment cost (Y3) | High severityᵍ | Exploratory, post-treatment | 942 | −0.523 | −0.675 to −0.370 | Yes |
| High insurance coverage (primary) | Log total treatment cost (Y3) | Private hospital | n/a | 100 | −0.257 | −0.604 to 0.091 | No |
| High insurance coverage (primary) | Log total treatment cost (Y3) | Public hospital | n/a | 1,713 | −0.521 | −0.629 to −0.413 | Yes |
| High insurance coverage (primary) | Log total treatment cost (Y3) | Rural | n/a | 547 | −0.512 | −0.703 to −0.322 | Yes |
| High insurance coverage (primary) | Log total treatment cost (Y3) | Urban | n/a | 1,266 | −0.504 | −0.629 to −0.380 | Yes |
| High insurance coverage (primary) | Log total treatment cost (Y3) | High SESʰ | n/a | 1,813 | −0.507 | −0.612 to −0.401 | Yes |
| Continuous claims exposure (secondary) | Unsuccessful-Discharge Proxy (Y2) | Age < 65 | Primary | 489 | −0.156 | −0.331 to 0.019 | No |
| Continuous claims exposure (secondary) | Unsuccessful-Discharge Proxy (Y2) | Age 65–74 | Primary | 567 | −0.176 | −0.293 to −0.059 | Yes |
| Continuous claims exposure (secondary) | Unsuccessful-Discharge Proxy (Y2) | Age ≥ 75 | n/a | 757 | −0.312 | −0.434 to −0.191 | Yes |
| Continuous claims exposure (secondary) | Unsuccessful-Discharge Proxy (Y2) | Low severityᵍ | Exploratory, post-treatment | 871 | −0.179 | −0.294 to −0.065 | Yes |
| Continuous claims exposure (secondary) | Unsuccessful-Discharge Proxy (Y2) | High severityᵍ | Exploratory, post-treatment | 942 | −0.273 | −0.378 to −0.169 | Yes |
| Continuous claims exposure (secondary) | Unsuccessful-Discharge Proxy (Y2) | Private hospital | n/a | 100 | −0.205 | −0.480 to 0.070 | No |
| Continuous claims exposure (secondary) | Unsuccessful-Discharge Proxy (Y2) | Public hospital | n/a | 1,713 | −0.222 | −0.306 to −0.138 | Yes |
| Continuous claims exposure (secondary) | Unsuccessful-Discharge Proxy (Y2) | Rural | n/a | 547 | −0.220 | −0.337 to −0.103 | Yes |
| Continuous claims exposure (secondary) | Unsuccessful-Discharge Proxy (Y2) | Urban | n/a | 1,266 | −0.222 | −0.323 to −0.121 | Yes |
| Continuous claims exposure (secondary) | Unsuccessful-Discharge Proxy (Y2) | High SESʰ | n/a | 1,813 | −0.221 | −0.300 to −0.142 | Yes |
| Continuous claims exposure (secondary) | Log total treatment cost (Y3) | Age < 65 | Primary | 489 | +2.463 | 2.063 to 2.864 | Yes |
| Continuous claims exposure (secondary) | Log total treatment cost (Y3) | Age 65–74 | Primary | 567 | +2.520 | 2.201 to 2.838 | Yes |
| Continuous claims exposure (secondary) | Log total treatment cost (Y3) | Age ≥ 75 | n/a | 757 | +2.589 | 2.317 to 2.861 | Yes |
| Continuous claims exposure (secondary) | Log total treatment cost (Y3) | Low severityᵍ | Exploratory, post-treatment | 871 | +2.385 | 2.073 to 2.696 | Yes |
| Continuous claims exposure (secondary) | Log total treatment cost (Y3) | High severityᵍ | Exploratory, post-treatment | 942 | +2.710 | 2.483 to 2.937 | Yes |
| Continuous claims exposure (secondary) | Log total treatment cost (Y3) | Private hospital | n/a | 100 | +1.672 | 0.922 to 2.422 | Yes |
| Continuous claims exposure (secondary) | Log total treatment cost (Y3) | Public hospital | n/a | 1,713 | +2.598 | 2.376 to 2.820 | Yes |
| Continuous claims exposure (secondary) | Log total treatment cost (Y3) | Rural | n/a | 547 | +2.700 | 2.338 to 3.062 | Yes |
| Continuous claims exposure (secondary) | Log total treatment cost (Y3) | Urban | n/a | 1,266 | +2.455 | 2.198 to 2.713 | Yes |
| Continuous claims exposure (secondary) | Log total treatment cost (Y3) | High SESʰ | n/a | 1,813 | +2.529 | 2.323 to 2.736 | Yes |

None of the subgroups shown had fewer than 100 patients, so no cell in this table meets the low-precision threshold (n < 100) that was otherwise flagged in the underlying analysis and displayed as an open marker on the corresponding forest plot. ᵍ Severity band is derived from the same post-treatment severity score described under Table 23 and is shown here as an exploratory stratifier only. ʰ The "High SES" row reproduces the full-cohort estimate (n = 1,813) rather than an independently SES-restricted subsample and should be read as a full-sample cross-check alongside the age-, severity-, sector-, and urbanicity-based subgroups, not as an SES-specific effect estimate.

Whether the exposures and outcomes examined above are distributed equitably across three independent socioeconomic proxies (insurance-fund category, a fixed socioeconomic-status rank, and rural residence) was evaluated using bootstrap concentration indices (S26 Table).

**S26 Table. Bootstrap concentration indices for recovery, the discharge-outcome proxy, treatment cost, out-of-pocket cost share, and length of stay, by socioeconomic proxy.**

| **Socioeconomic proxy** | **Outcome** | **n** | **Concentration index (CIndex)ⁱ** | **95% CI** | **p-value** | **Direction** | **95% CI excludes 0** |
| --- | --- | --- | --- | --- | --- | --- | --- |
| Insurance-fund category | Recovery (Y1) | 1,813 | +0.087 | 0.048 to 0.129 | <0.001 | Pro-rich | Yes |
| Insurance-fund category | Unsuccessful-Discharge Proxy (Y2) | 1,813 | −0.022 | −0.055 to 0.014 | 0.204 | Pro-poor | No |
| Insurance-fund category | Log total treatment cost (Y3) | 1,813 | −0.006 | −0.008 to −0.005 | <0.001 | Pro-poor | Yes |
| Insurance-fund category | Patient out-of-pocket share | 1,813 | −0.056 | −0.077 to −0.035 | <0.001 | Pro-poor | Yes |
| Insurance-fund category | Length of stay (days) | 1,813 | −0.046 | −0.078 to −0.020 | 0.001 | Pro-poor | Yes |
| Fixed socioeconomic-status rank | Recovery (Y1) | 1,813 | +0.053 | 0.009 to 0.095 | 0.020 | Pro-rich | Yes |
| Fixed socioeconomic-status rank | Unsuccessful-Discharge Proxy (Y2) | 1,813 | +0.004 | −0.029 to 0.039 | 0.831 | Pro-rich | No |
| Fixed socioeconomic-status rank | Log total treatment cost (Y3) | 1,813 | −0.004 | −0.006 to −0.003 | <0.001 | Pro-poor | Yes |
| Fixed socioeconomic-status rank | Patient out-of-pocket share | 1,813 | −0.043 | −0.063 to −0.023 | <0.001 | Pro-poor | Yes |
| Fixed socioeconomic-status rank | Length of stay (days) | 1,813 | −0.027 | −0.055 to 0.002 | 0.079 | Pro-poor | No |
| Rural residence | Recovery (Y1) | 1,813 | +0.062 | 0.028 to 0.096 | 0.001 | Pro-rich | Yes |
| Rural residence | Unsuccessful-Discharge Proxy (Y2) | 1,813 | −0.029 | −0.053 to −0.003 | 0.026 | Pro-poor | Yes |
| Rural residence | Log total treatment cost (Y3) | 1,813 | −0.003 | −0.005 to −0.002 | <0.001 | Pro-poor | Yes |
| Rural residence | Patient out-of-pocket share | 1,813 | −0.060 | −0.076 to −0.044 | <0.001 | Pro-poor | Yes |
| Rural residence | Length of stay (days) | 1,813 | −0.036 | −0.056 to −0.014 | 0.001 | Pro-poor | Yes |

*ⁱ A positive concentration index indicates that the outcome is concentrated among socioeconomically better-off patients (pro-rich); a negative value indicates concentration among worse-off patients (pro-poor). The index is bounded between −1 and 1, with 0 indicating no socioeconomic gradient.*

The direction and magnitude of the equity gradients were consistent across all three socioeconomic proxies: recovery was mildly pro-rich, while cost, out-of-pocket burden, and length of stay were consistently pro-poor (i.e., concentrated among worse-off patients), and the discharge-outcome-proxy gradient was small and not statistically distinguishable from zero for two of the three proxies. Because these are unconditional, cross-sectional associations rather than treatment effects, they are reported as descriptive equity benchmarks against which the welfare-optimisation targeting ratios in Table 23 can be interpreted, rather than as evidence of a causal equity effect of either exposure.

The welfare-optimisation credible intervals in Table 23 were generated using a greedy heuristic allocation solver in each bootstrap replicate, for tractability; the resulting gap between this heuristic and the exact ILP solution used for the base-case point estimate is quantified in S27 Table.

**S27 Table. Comparison of the exact integer-linear-programming solution and the greedy heuristic used to construct the welfare-optimisation bootstrap confidence intervals**

| **Exposure (causal role)** | **Allocation method** | **N treated (%)** | **Welfare, W** | **Jaccard overlap with ILP optimum** | **ΔW vs. ILP optimum** | **Δ% treated vs. ILP optimum (pp)** |
| --- | --- | --- | --- | --- | --- | --- |
| Prolonged length of stay (exploratory) | Exact ILP (base case, optimal) | 495 (27.3%) | 487.34 | — (reference) | — | — |
| Prolonged length of stay (exploratory) | Greedy heuristic (bootstrap) | 404 (22.3%) | 430.34 | 0.739 | +57.00 | +5.02 |
| High insurance coverage (primary) | Exact ILP (base case, optimal) | 649 (35.8%) | 290.69 | — (reference) | — | — |
| High insurance coverage (primary) | Greedy heuristic (bootstrap) | 504 (27.8%) | 245.82 | 0.606 | +44.87 | +8.00 |

*Cohort totals underlying this comparison: N = 1,813 episodes; total bed-days = 9,868; total insurance expenditureʲ = 13,731,432,629 IRR (≈ 91,543 Int$). ʲ Converted using a purchasing-power-parity (PPP) factor of 150,000 IRR per international dollar, the approximate midpoint of the 2024–2025 range (146,000–150,000 IRR/Int$ for 2024; 150,000–155,000 IRR/Int$ for 2025) pending final International Comparison Program updates; costs are assumed to be recorded in Iranian rial, consistent with the dataset's institutional context.*

The greedy heuristic reproduces the deterministic greedy allocation exactly in both scenarios (Jaccard overlap = 1.000, ΔW = 0 relative to that benchmark, not shown as a separate row), confirming that the bootstrap procedure itself is implemented correctly; however, the heuristic recovers only 60.6–73.9% of the patients selected by the exact optimum and understates achievable welfare by 44.9–57.0 points (roughly 9–15% of the base-case welfare value). Because the base-case point estimates reported in Table 23 use the exact ILP solution while the accompanying 95% credible intervals are built from the heuristic, the credible intervals in Table 23 should be read as a conservative (upper-bound-shifted) characterisation of bootstrap variability around the exact optimum, rather than as intervals computed directly around a re-optimised ILP solution in every replicate.

**S19 Figure. Bootstrap Distribution of T-prolonged Coefficients (Layer 1) – All Estimates Equal**

**S20 Figure. Concentration Indices for Health Outcomes and Resource Use by Insurance Fund Enrollment (Layer 2)**

**S21 Figure. Treatment Rates and 95% Confidence Intervals for Primary and Exploratory Subgroups in the Age-Matched Cohort (≥75 Years, Step 5 v4)**

**S22 Figure. Comprehensive uncertainty analysis of causal treatment effects and optimized intervention allocation strategies**

**S23 Figure. Heterogeneity of treatment-associated effects of prolonged length of stay across patient subgroups**
**S24 Figure. Heterogeneity in Treatment Effects of High Insurance Coverage: Bootstrap BCa 95% CIs Across Age, Severity, Hospital Type, Location, and SES**

**S25 Figure. E-Value Sensitivity Analysis for Unmeasured Confounding Across Primary, Secondary, and Exploratory Outcome Proxies (Layer 3)**

**Sensitivity to Within-Patient Dependence**

**S28 Table. Breaking-point intraclass correlation (ICC) for within-patient dependence, for all 66 fitted average-treatment-effect models, by assumed average within-patient cluster size.**

*"Robust" = no ICC value up to the grid maximum of 0.50 overturns significance at that cluster size. "n/a — NS" = already non-significant at the naive (unclustered) standard error, so no breaking ICC applies. Numeric entries give the exact breaking ICC.*

| **Exposure (causal role)** | **Outcome** | **Scope** | **n** | **ATE** | **Naive SE** | **Sig. (naive)** | ***m*=1.1** | ***m*=1.25** | ***m*=1.5** | ***m*=2.0** | ***m*=3.0** |
| --- | --- | --- | --- | --- | --- | --- | --- | --- | --- | --- | --- |
| Prolonged LOS (exploratory) | Unsuccessful-Discharge Proxy (Y2) | Main | 1,812 | −0.064 | 0.012 | Yes | Robust | Robust | Robust | Robust | Robust |
| Prolonged LOS (exploratory) | Log total cost (Y3) | Main | 1,812 | +1.099 | 0.028 | Yes | Robust | Robust | Robust | Robust | Robust |
| High coverage (primary) | Unsuccessful-Discharge Proxy (Y2) | Main | 1,813 | +0.022 | 0.014 | No | n/a — NS | n/a — NS | n/a — NS | n/a — NS | n/a — NS |
| High coverage (primary) | Log total cost (Y3) | Main | 1,813 | −0.507 | 0.035 | Yes | Robust | Robust | Robust | Robust | Robust |
| Continuous exposure (secondary) | Unsuccessful-Discharge Proxy (Y2) | Main | 1,722 | −0.221 | 0.040 | Yes | Robust | Robust | Robust | Robust | Robust |
| Continuous exposure (secondary) | Log total cost (Y3) | Main | 1,722 | +2.529 | 0.088 | Yes | Robust | Robust | Robust | Robust | Robust |
| Prolonged LOS (exploratory) | Unsuccessful-Discharge Proxy (Y2) | Age < 65 | 489 | −0.086 | 0.022 | Yes | Robust | Robust | Robust | Robust | Robust |
| Prolonged LOS (exploratory) | Unsuccessful-Discharge Proxy (Y2) | Age 65–74 | 567 | −0.043 | 0.022 | No | n/a — NS | n/a — NS | n/a — NS | n/a — NS | n/a — NS |
| Prolonged LOS (exploratory) | Unsuccessful-Discharge Proxy (Y2) | Age ≥ 75 | 757 | −0.064 | 0.019 | Yes | Robust | Robust | Robust | Robust | Robust |
| Prolonged LOS (exploratory) | Unsuccessful-Discharge Proxy (Y2) | Low severityᵃ | 871 | −0.076 | 0.018 | Yes | Robust | Robust | Robust | Robust | Robust |
| Prolonged LOS (exploratory) | Unsuccessful-Discharge Proxy (Y2) | High severityᵃ | 942 | −0.053 | 0.016 | Yes | Robust | Robust | Robust | Robust | Robust |
| Prolonged LOS (exploratory) | Unsuccessful-Discharge Proxy (Y2) | Private hospitalᵇ | 100 | −0.066 | 0.033 | Yes | 0.425 | 0.170 | 0.085 | 0.045 | 0.025 |
| Prolonged LOS (exploratory) | Unsuccessful-Discharge Proxy (Y2) | Public hospital | 1,713 | −0.064 | 0.013 | Yes | Robust | Robust | Robust | Robust | Robust |
| Prolonged LOS (exploratory) | Unsuccessful-Discharge Proxy (Y2) | Rural | 547 | −0.063 | 0.018 | Yes | Robust | Robust | Robust | Robust | Robust |
| Prolonged LOS (exploratory) | Unsuccessful-Discharge Proxy (Y2) | Urban | 1,266 | −0.064 | 0.015 | Yes | Robust | Robust | Robust | Robust | Robust |
| Prolonged LOS (exploratory) | Unsuccessful-Discharge Proxy (Y2) | High SESᶜ | 1,813 | −0.064 | 0.012 | Yes | Robust | Robust | Robust | Robust | Robust |
| Prolonged LOS (exploratory) | Log total cost (Y3) | Age < 65 | 489 | +1.092 | 0.083 | Yes | Robust | Robust | Robust | Robust | Robust |
| Prolonged LOS (exploratory) | Log total cost (Y3) | Age 65–74 | 567 | +1.113 | 0.066 | Yes | Robust | Robust | Robust | Robust | Robust |
| Prolonged LOS (exploratory) | Log total cost (Y3) | Age ≥ 75 | 757 | +1.094 | 0.041 | Yes | Robust | Robust | Robust | Robust | Robust |
| Prolonged LOS (exploratory) | Log total cost (Y3) | Low severityᵃ | 871 | +1.022 | 0.055 | Yes | Robust | Robust | Robust | Robust | Robust |
| Prolonged LOS (exploratory) | Log total cost (Y3) | High severityᵃ | 942 | +1.170 | 0.058 | Yes | Robust | Robust | Robust | Robust | Robust |
| Prolonged LOS (exploratory) | Log total cost (Y3) | Private hospitalᵇ | 100 | +0.609 | 0.096 | Yes | Robust | Robust | Robust | Robust | Robust |
| Prolonged LOS (exploratory) | Log total cost (Y3) | Public hospital | 1,713 | +1.132 | 0.042 | Yes | Robust | Robust | Robust | Robust | Robust |
| Prolonged LOS (exploratory) | Log total cost (Y3) | Rural | 547 | +1.117 | 0.068 | Yes | Robust | Robust | Robust | Robust | Robust |
| Prolonged LOS (exploratory) | Log total cost (Y3) | Urban | 1,266 | +1.092 | 0.049 | Yes | Robust | Robust | Robust | Robust | Robust |
| Prolonged LOS (exploratory) | Log total cost (Y3) | High SESᶜ | 1,813 | +1.099 | 0.040 | Yes | Robust | Robust | Robust | Robust | Robust |
| High coverage (primary) | Unsuccessful-Discharge Proxy (Y2) | Age < 65 | 489 | −0.001 | 0.032 | No | n/a — NS | n/a — NS | n/a — NS | n/a — NS | n/a — NS |
| High coverage (primary) | Unsuccessful-Discharge Proxy (Y2) | Age 65–74 | 567 | +0.013 | 0.029 | No | n/a — NS | n/a — NS | n/a — NS | n/a — NS | n/a — NS |
| High coverage (primary) | Unsuccessful-Discharge Proxy (Y2) | Age ≥ 75 | 757 | +0.043 | 0.023 | No | n/a — NS | n/a — NS | n/a — NS | n/a — NS | n/a — NS |
| High coverage (primary) | Unsuccessful-Discharge Proxy (Y2) | Low severityᵃ | 871 | +0.005 | 0.023 | No | n/a — NS | n/a — NS | n/a — NS | n/a — NS | n/a — NS |
| High coverage (primary) | Unsuccessful-Discharge Proxy (Y2) | High severityᵃ | 942 | +0.037 | 0.023 | No | n/a — NS | n/a — NS | n/a — NS | n/a — NS | n/a — NS |
| High coverage (primary) | Unsuccessful-Discharge Proxy (Y2) | Private hospitalᵇ | 100 | +0.045 | 0.056 | No | n/a — NS | n/a — NS | n/a — NS | n/a — NS | n/a — NS |
| High coverage (primary) | Unsuccessful-Discharge Proxy (Y2) | Public hospital | 1,713 | +0.020 | 0.017 | No | n/a — NS | n/a — NS | n/a — NS | n/a — NS | n/a — NS |
| High coverage (primary) | Unsuccessful-Discharge Proxy (Y2) | Rural | 547 | 0.000 | 0.026 | No | n/a — NS | n/a — NS | n/a — NS | n/a — NS | n/a — NS |
| High coverage (primary) | Unsuccessful-Discharge Proxy (Y2) | Urban | 1,266 | +0.031 | 0.020 | No | n/a — NS | n/a — NS | n/a — NS | n/a — NS | n/a — NS |
| High coverage (primary) | Unsuccessful-Discharge Proxy (Y2) | High SESᶜ | 1,813 | +0.022 | 0.016 | No | n/a — NS | n/a — NS | n/a — NS | n/a — NS | n/a — NS |
| High coverage (primary) | Log total cost (Y3) | Age < 65 | 489 | −0.488 | 0.114 | Yes | Robust | Robust | Robust | Robust | Robust |
| High coverage (primary) | Log total cost (Y3) | Age 65–74 | 567 | −0.469 | 0.089 | Yes | Robust | Robust | Robust | Robust | Robust |
| High coverage (primary) | Log total cost (Y3) | Age ≥ 75 | 757 | −0.547 | 0.054 | Yes | Robust | Robust | Robust | Robust | Robust |
| High coverage (primary) | Log total cost (Y3) | Low severityᵃ | 871 | −0.489 | 0.072 | Yes | Robust | Robust | Robust | Robust | Robust |
| High coverage (primary) | Log total cost (Y3) | High severityᵃ | 942 | −0.523 | 0.078 | Yes | Robust | Robust | Robust | Robust | Robust |
| High coverage (primary) | Log total cost (Y3) | Private hospitalᵇ | 100 | −0.257 | 0.177 | No | n/a — NS | n/a — NS | n/a — NS | n/a — NS | n/a — NS |
| High coverage (primary) | Log total cost (Y3) | Public hospital | 1,713 | −0.521 | 0.055 | Yes | Robust | Robust | Robust | Robust | Robust |
| High coverage (primary) | Log total cost (Y3) | Rural | 547 | −0.512 | 0.097 | Yes | Robust | Robust | Robust | Robust | Robust |
| High coverage (primary) | Log total cost (Y3) | Urban | 1,266 | −0.504 | 0.064 | Yes | Robust | Robust | Robust | Robust | Robust |
| High coverage (primary) | Log total cost (Y3) | High SESᶜ | 1,813 | −0.507 | 0.054 | Yes | Robust | Robust | Robust | Robust | Robust |
| Continuous exposure (secondary) | Unsuccessful-Discharge Proxy (Y2) | Age < 65 | 489 | −0.156 | 0.089 | No | n/a — NS | n/a — NS | n/a — NS | n/a — NS | n/a — NS |
| Continuous exposure (secondary) | Unsuccessful-Discharge Proxy (Y2) | Age 65–74 | 567 | −0.176 | 0.060 | Yes | Robust | Robust | Robust | Robust | Robust |
| Continuous exposure (secondary) | Unsuccessful-Discharge Proxy (Y2) | Age ≥ 75 | 757 | −0.312 | 0.062 | Yes | Robust | Robust | Robust | Robust | Robust |
| Continuous exposure (secondary) | Unsuccessful-Discharge Proxy (Y2) | Low severityᵃ | 871 | −0.179 | 0.058 | Yes | Robust | Robust | Robust | Robust | Robust |
| Continuous exposure (secondary) | Unsuccessful-Discharge Proxy (Y2) | High severityᵃ | 942 | −0.273 | 0.053 | Yes | Robust | Robust | Robust | Robust | Robust |
| Continuous exposure (secondary) | Unsuccessful-Discharge Proxy (Y2) | Private hospitalᵇ | 100 | −0.205 | 0.140 | No | n/a — NS | n/a — NS | n/a — NS | n/a — NS | n/a — NS |
| Continuous exposure (secondary) | Unsuccessful-Discharge Proxy (Y2) | Public hospital | 1,713 | −0.222 | 0.043 | Yes | Robust | Robust | Robust | Robust | Robust |
| Continuous exposure (secondary) | Unsuccessful-Discharge Proxy (Y2) | Rural | 547 | −0.220 | 0.060 | Yes | Robust | Robust | Robust | Robust | Robust |
| Continuous exposure (secondary) | Unsuccessful-Discharge Proxy (Y2) | Urban | 1,266 | −0.222 | 0.052 | Yes | Robust | Robust | Robust | Robust | Robust |
| Continuous exposure (secondary) | Unsuccessful-Discharge Proxy (Y2) | High SESᶜ | 1,813 | −0.221 | 0.040 | Yes | Robust | Robust | Robust | Robust | Robust |
| Continuous exposure (secondary) | Log total cost (Y3) | Age < 65 | 489 | +2.463 | 0.204 | Yes | Robust | Robust | Robust | Robust | Robust |
| Continuous exposure (secondary) | Log total cost (Y3) | Age 65–74 | 567 | +2.520 | 0.162 | Yes | Robust | Robust | Robust | Robust | Robust |
| Continuous exposure (secondary) | Log total cost (Y3) | Age ≥ 75 | 757 | +2.589 | 0.139 | Yes | Robust | Robust | Robust | Robust | Robust |
| Continuous exposure (secondary) | Log total cost (Y3) | Low severityᵃ | 871 | +2.385 | 0.159 | Yes | Robust | Robust | Robust | Robust | Robust |
| Continuous exposure (secondary) | Log total cost (Y3) | High severityᵃ | 942 | +2.710 | 0.116 | Yes | Robust | Robust | Robust | Robust | Robust |
| Continuous exposure (secondary) | Log total cost (Y3) | Private hospitalᵇ | 100 | +1.672 | 0.383 | Yes | Robust | Robust | Robust | Robust | Robust |
| Continuous exposure (secondary) | Log total cost (Y3) | Public hospital | 1,713 | +2.598 | 0.113 | Yes | Robust | Robust | Robust | Robust | Robust |
| Continuous exposure (secondary) | Log total cost (Y3) | Rural | 547 | +2.700 | 0.185 | Yes | Robust | Robust | Robust | Robust | Robust |
| Continuous exposure (secondary) | Log total cost (Y3) | Urban | 1,266 | +2.455 | 0.131 | Yes | Robust | Robust | Robust | Robust | Robust |
| Continuous exposure (secondary) | Log total cost (Y3) | High SESᶜ | 1,813 | +2.529 | 0.105 | Yes | Robust | Robust | Robust | Robust | Robust |

*ᵃ Severity band is derived from the post-treatment severity score described under Table 23 and is shown here as an exploratory stratifier only. ᵇ The private-hospital subgroup (n = 100) contains the only model in this table with a finite breaking ICC below the grid maximum; at an assumed average cluster size of m = 1.1 episodes per patient, a within-patient correlation of only ICC ≈ 0.425 would be sufficient to overturn its significance, falling to ICC ≈ 0.025 at m = 3.0. This subgroup already carries the widest confidence interval among the length-of-stay subgroups (S25 Table) and should be interpreted with corresponding caution regardless of the clustering assumption used. ᶜ The "High SES" row reproduces the full-cohort estimate (n = 1,813) rather than an independently SES-restricted subsample, consistent with the same caveat noted for this row in S25 Table.*

**References**

1.	Belsley DA, Kuh E, Welsch RE. Regression diagnostics: identifying influential data and sources of collinearity. New York: Wiley; 1980.

2.	Chen T, Guestrin C. XGBoost: A Scalable Tree Boosting System. Proceedings of the 22nd ACM SIGKDD International Conference on Knowledge Discovery and Data Mining; San Francisco, California, USA: Association for Computing Machinery; 2016. p. 785–94.

3.	Jørgensen, B., & Paes De Souza, M. C. (1994). Fitting Tweedie’s compound poisson model to insurance claims data. Scandinavian Actuarial Journal, 1994(1), 69–93. <https://doi.org/10.1080/03461238.1994.10413930>.

4.	Bergstra, J., Bardenet, R., Bengio, Y. and Kégl, B. (2011) Algorithms for Hyper-Parameter Optimization. Proceedings of the 24th International Conference on Neural Information Processing Systems, Granada, 12-15 December 2011, 2546-2554. <https://proceedings.neurips.cc/paper_files/paper/2011/file/86e8f7ab32cfd12577bc2619bc635690-Paper.pdf>.

5.	Bergstra, J. and Bengio, Y. (2012) Random Search for Hyper-Parameter Optimization. Journal of Machine Learning Research, 13, 281-305.

6.	Brier, G.W. (1950) Verification of Forecasts Expressed in Terms of Probability. Monthly Weather Review, 78, 1-3.

7.	Niculescu-Mizil A, Caruana R. Predicting good probabilities with supervised learning. Proceedings of the 22nd international conference on Machine learning; Bonn, Germany: Association for Computing Machinery; 2005. p. 625–32.

8.	Lundberg, S.M. and Lee, S.-I. (2017) A Unified Approach to Interpreting Model Predictions. Proceedings of the 31st International Conference on Neural Information Processing Systems, Long Beach, 4-9 December 2017, 4766-4777.

9.	Strobl, C., Boulesteix, AL., Zeileis, A. et al. Bias in random forest variable importance measures: Illustrations, sources and a solution. BMC Bioinformatics 8, 25 (2007). <https://doi.org/10.1186/1471-2105-8-25>.

10.	Victor Chernozhukov, Denis Chetverikov, Mert Demirer, Esther Duflo, Christian Hansen, Whitney Newey, James Robins, Double/debiased machine learning for treatment and structural parameters, The Econometrics Journal, Volume 21, Issue 1, 1 February 2018, Pages C1–C68, <https://doi.org/10.1111/ectj.12097>.

11.	Robins, J.M. and Rotnitzky, A. (1994) Estimation of Regression Coefficients When Some Regressors Are Not Always Observed. Journal of the American Statistical Association, 89, 846-866. <http://dx.doi.org/10.1080/01621459.1994.10476818>.

12.	Newey W, McFadden D. Large sample estimation and hypothesis testing. In: Engle RF, McFadden D, editors. Handbook of Econometrics. 4: Elsevier; 1986. p. 2111–245.

13.	White, H. (1980) A Heteroskedasticity-Consistent Covariance Matrix and a Direct Test for Heteroskedasticity. Econometrica, 48, 817-838.

<https://doi.org/10.2307/1912934>.

14.	Richard K. Crump, V. Joseph Hotz, Guido W. Imbens, Oscar A. Mitnik, Dealing with limited overlap in estimation of average treatment effects, Biometrika, Volume 96, Issue 1, March 2009, Pages 187–199, <https://doi.org/10.1093/biomet/asn055>.

15.	Wagstaff A, van Doorslaer E. Catastrophe and impoverishment in paying for health care: with applications to Vietnam 1993-1998. Health Econ. 2003 Nov;12(11):921-34. doi: 10.1002/hec.776. PMID: 14601155.

16.	Cohen SB, editor The concentration and persistence in the level of health expenditures over time: Estimates for the U.S. population, 2006-20072010.

17.	Li F, Thomas LE, Li F. Addressing Extreme Propensity Scores via the Overlap Weights. Am J Epidemiol. 2019 Jan 1;188(1):250-257. doi: 10.1093/aje/kwy201. Erratum in: Am J Epidemiol. 2021 Jan 4;190(1):189-190. doi: 10.1093/aje/kwaa229. PMID: 30189042.

18.	Staiger, D. and Stock, J.H. (1997) Instrumental Variables Regression with Weak Instruments. Econometrica, 65, 557-586. <https://doi.org/10.2307/2171753>.

19.	Wager, S., & Athey, S. (2018). Estimation and Inference of Heterogeneous Treatment Effects using Random Forests. Journal of the American Statistical Association, 113(523), 1228–1242. <https://doi.org/10.1080/01621459.2017.1319839>.

20.	Drummond MF, Sculpher MJ, Claxton K, Stoddart GL, Torrance GW. Methods for the economic evaluation of health care programmes. 4th ed. Oxford: Oxford University Press; 2015.

21.	Künzel SR, Sekhon JS, Bickel PJ, Yu B. Metalearners for estimating heterogeneous treatment effects using machine learning. Proc Natl Acad Sci U S A. 2019 Mar 5;116(10):4156-4165. doi: 10.1073/pnas.1804597116. Epub 2019 Feb 15. PMID: 30770453; PMCID: PMC6410831.

22.	Athey S, Imbens G. Recursive partitioning for heterogeneous causal effects. Proc Natl Acad Sci U S A. 2016 Jul 5;113(27):7353-60. doi: 10.1073/pnas.1510489113. PMID: 27382149; PMCID: PMC4941430.

23.	Erreygers G. Correcting the concentration index. J Health Econ. 2009 Mar;28(2):504-15. doi: 10.1016/j.jhealeco.2008.02.003. Epub 2008 Feb 16. PMID: 18367273.

24.	Kjellsson G, Gerdtham UG. On correcting the concentration index for binary variables. J Health Econ. 2013 May;32(3):659-70. doi: 10.1016/j.jhealeco.2012.10.012. Epub 2012 Nov 8. PMID: 23522656.

25.	van Doorslaer E, Wagstaff A, van der Burg H, Christiansen T, De Graeve D, Duchesne I, Gerdtham UG, Gerfin M, Geurts J, Gross L, Häkkinen U, John J, Klavus J, Leu RE, Nolan B, O'Donnell O, Propper C, Puffer F, Schellhorn M, Sundberg G, Winkelhake O. Equity in the delivery of health care in Europe and the US. J Health Econ. 2000 Sep;19(5):553-83. doi: 10.1016/s0167-6296(00)00050-3. PMID: 11184794.

26.	O’Donnell, O., van Doorslaer, E., Wagstaff, A., and Lindelow, M. (2008). Analyzing Health Equity Using Household Survey Data: A Guide to Techniques and Their Implementation. World Bank Publications. <https://doi.org/10.1596/978-0-8213-6933-3>.

27.	Forrest, J., & Lougee-Heimer, R. (2005). CBC User Guide. In Emerging Theory, Methods, and Applications (pp. 257–277). INFORMS. <https://doi.org/10.1287/educ.1053.0020>.

28.	Culyer AJ, Wagstaff A. Equity and equality in health and health care. J Health Econ. 1993 Dec;12(4):431-57. doi: 10.1016/0167-6296(93)90004-x. PMID: 10131755.

29.	Williams S, Prior SJ. Health Service Improvement for People with Parkinson’s Disease: A Scoping Review. Journal of Ageing and Longevity. 2024; 4(3):234-251. <https://doi.org/10.3390/jal4030017>.

30.	Efron B, Hastie T. Computer Age Statistical Inference: Algorithms, Evidence, and Data Science. Cambridge: Cambridge University Press; 2016. (Institute of Mathematical Statistics Monographs).

31.	Efron, B. and DiCiccio, T. (1996) Bootstrap Confidence Intervals. Statistical Science, 11, 189-228. <http://dx.doi.org/10.1214/ss/1032280214>.

32.	Rosenbaum, P.R. (2002) Observational Studies. 2nd Edition, Springer-Verlag, New York.
